# Steroid Metabolism in Thermophilic Actinobacterium *Saccharopolyspora hirsuta* VKM Ac-666^T^

**DOI:** 10.3390/microorganisms9122554

**Published:** 2021-12-10

**Authors:** Tatyana Lobastova, Victoria Fokina, Sergey Tarlachkov, Andrey Shutov, Eugeny Bragin, Alexey Kazantsev, Marina Donova

**Affiliations:** 1 G.K. Skryabin Institute of Biochemistry and Physiology of Microorganisms, Federal Research Center “Pushchino Scientific Center for Biological Research of the Russian Academy of Sciences”, Pr. Nauki 5, 142290 Pushchino, Russia; lobastova_t@rambler.ru (T.L.); sergey@tarlachkov.ru (S.T.); w___w@rambler.ru (A.S.); bragory@yandex.ru (E.B.); mv_donova@rambler.ru (M.D.); 2 Chemistry Department, Lomonosov Moscow State University, Leninskie Gory 1/3, 119991 Moscow, Russia; mak@org.chem.msu.ru

**Keywords:** thermophilic actinobacteria, steroids, sterol catabolism, cholate, *Saccharopolyspora hirsuta*, bioconversion

## Abstract

The application of thermophilic microorganisms opens new prospects in steroid biotechnology, but little is known to date on steroid catabolism by thermophilic strains. The thermophilic strain *Saccharopolyspora hirsuta* VKM Ac-666^T^ has been shown to convert various steroids and to fully degrade cholesterol. Cholest-4-en-3-one, cholesta-1,4-dien-3-one, 26-hydroxycholest-4-en-3-one, 3-oxo-cholest-4-en-26-oic acid, 3-oxo-cholesta-1,4-dien-26-oic acid, 26-hydroxycholesterol, 3β-hydroxy-cholest-5-en-26-oic acid were identified as intermediates in cholesterol oxidation. The structures were confirmed by ^1^H and ^13^C-NMR analyses. Aliphatic side chain hydroxylation at C26 and the A-ring modification at C3, which are putatively catalyzed by cytochrome P450 monooxygenase CYP125 and cholesterol oxidase, respectively, occur simultaneously in the strain and are followed by cascade reactions of aliphatic sidechain degradation and steroid core destruction via the known 9(10)-*seco*-pathway. The genes putatively related to the sterol and bile acid degradation pathways form three major clusters in the *S. hirsuta* genome. The sets of the genes include the orthologs of those involved in steroid catabolism in *Mycobacterium tuberculosis* H37Rv and *Rhodococcus jostii* RHA1 and related actinobacteria. Bioinformatics analysis of 52 publicly available genomes of thermophilic bacteria revealed only seven candidate strains that possess the key genes related to the 9(10)-*seco* pathway of steroid degradation, thus demonstrating that the ability to degrade steroids is not widespread among thermophilic bacteria.

## 1. Introduction

Steroids are abundant biomolecules in various environments and growth substrates for diverse bacteria. Sterols (e.g., cholesterol, ergosterol, and phytosterols) are steroid 3β-alcohols with an alkyl side chain consisting of 8–10 carbon atoms. Structurally, bile acids differ from sterols by *cis*-A/B-ring juncture, α-orientation of hydroxyl at C3, a saturated steroid core, and a C5 acyl side chain. Due to the unique lipophilic/amphiphilic properties, steroidal compounds play vital functions in all living organisms. Annually large amounts of sterols, bile acids, and other steroids enter into the environment via the decay of biomass or excretion by humans and animals and as industrial wastes of steroid production plants.

Modern bioinformatics studies of publicly available genomes/metagenomes have highlighted the global distribution of actinobacteria capable of sterol and cholate degradation from various ecological niches (soil, aquatic environments, waste, etc.) [1,2]. Currently, the so-called 9(10)-*seco*-steroid pathway is the only one known for sterol and cholate aerobic degradation by actinobacteria [3,4,5,6]. This pathway has been intensively studied in the pathogenic species *M. tuberculosis* [7] and *Rhodococcus* strains [8,9] and the non-pathogenic species *Mycolicibacterium smegmatis* mc^2^155 [10], *Gordonia cholesterolivorans* [11], and *Nocardioides simplex* [12]. Aerobic cholate degradation has been mainly studied for *Rhodococcus* strains (e.g., *R. jostii* RHA1) [13] and *Pseudomonas stutzeri* Chol1 [14], *Pseudomonas putida* DOC21 [15], and *Comamonas testosteroni* TA441 [16]. In general, the molecular mechanisms of steroid catabolism have been studied mainly for mesophilic actinobacteria, while little is known about the features of thermophilic actinobacteria capable of steroid oxidation.

Thermophilic microorganisms and their enzymes are widely used in the production of foods and detergents and the pulp and paper, textile, and mining industries [17]. An impressive example is provided by Taq polymerase (named after *Thermus aquaticus*), which is indispensable for PCR techniques in medicine and biology [18]. Application of thermophilic strains for steroid bioconversion is of great importance since it may provide economically feasible biotechnologies by decreasing the production costs for bioreactor cooling, especially in countries with hot climate. Besides, higher steroid solubility at elevated temperatures is favorable for steroid bioconversion performance. However, data on steroid bioconversion by thermophilic bacteria are scarce and pertain mainly to distinct reactions, such as progesterone conversion by *Bacillus thermoglucosidasius* (=*Parageobacillus thermoglucosidasius*) [19] and *Geobacillus kaustophilus* [20] and reduction of the 3-keto group as well as the Δ^4^-double bond in various steroid ketones by the extremely thermophilic bacterium *Calderiella acidophila* [21].

The moderately thermophilic *Saccharopolyspora hirsuta* VKM Ac-666^T^ [22] is capable of transforming various steroids, such as lithocholic acid (LCA) [23], dehydroepiandrosterone, androstenedione, and 3β,7(α/β)-dihydroxy-5-ene-d-homo-lactones [24]. Recently, the Ac-666^T^ genome has been sequenced and preliminary annotated [25].

In this work, aerobic conversion of cholesterol and LCA by *S. hirsuta* VKM Ac-666^T^ was studied and the main intermediates were identified. The set of the genes putatively involved in sterol and cholate catabolism pathways was revealed, and their organization and clustering were examined. The presence of genes coding for key steroid degradation enzymes was estimated in the genomes of thermophilic bacteria of different taxa, and potent microbial steroid degraders that might function at elevated temperatures were predicted.

## 2. Materials and Methods

### 2.1. Materials

Cholesterol (Serva, Heidelberg, Germany), lithocholic acid (LCA) from Acros Organics (Merelbeke, Belgium), cholestenone from Maybridge (Altrincham, UK), randomly methylated β-cyclodextrin (MCD) from Wacker-Chemie GmbH (Munich, Germany), malt extract for microbiology and corn steep solids from Sigma-Aldrich (St. Louis, MO, USA), and soluble starch and yeast extract from Difco (Franklin Lakes, NJ, USA) were used. Other materials and solvents were of analytical grade and were purchased from commercial suppliers.

### 2.2. Microorganism

The strain *Saccharopolyspora hirsuta* VKM Ac-666^T^ was obtained from the All-Russian Collection of Microorganisms (VKM).

### 2.3. Microorganism Cultivation and Cholesterol Conversion

The GSMY medium [26], which contained (g/L): glucose, 7; soluble starch, 10; malt extract, 5; yeast extract, 4.5; and CaCO_3_, 0.05 (pH 7.0–7.2), was used because its positive effect on the accumulation of intermediate products of bioconversion was shown in preliminary experiments. The strain was grown in shake flasks (750 mL) containing 50 mL of the GSMY medium aerobically (200 rpm) at 45 °C for 48 h. The resulting seed culture (5 mL) was added into shake flasks containing 50 mL of the same medium. Each steroid (cholesterol or LCA) was added as a solution in MCD to a final concentration of 0.5 g/L 24 h after inoculation. Molar ratios (steroid:MCD) were 1:5 or 1:3 for cholesterol and LCA, respectively. Bioconversion was carried out aerobically (200 rpm) at 45 °C for 144 h. For growth estimation, the strain was incubated on GSMY agar slants at 20, 30, 37, 45, 50, 55, and 60 °C for 24 h.

For biomass estimation, the samples of the broth (24 h) were centrifuged, the residue was washed twice with distilled water, and then it was dried at 105 °C to constant weight.

The experiments were performed in triplicate.

### 2.4. Steroid Metabolite Isolation and Identification

After 48 and 144 h of cholesterol conversion, steroids were extracted from the supernatant (~500 mL; 8000× *g*, 30 min) with ethyl acetate (250 mL) three times and the pooled organic extract was concentrated on a rotary evaporator. Crude residues (25–30 mg) were applied on preparative thin layer chromatography (TLC) plates (ALUGRAM SIL G-200 UV254, Macherey-Nagel, Düren, Germany) and developed in benzene:acetone (4:1, *v*/*v*). Individual compounds were eluted with ethyl acetate and evaporated to dryness. Chromatographic purity of the compounds was controlled by TLC and HPLC. Lithocholic acid bioconversion metabolites formed in small amounts and were not isolated and identified because their accumulation was insufficient.

### 2.5. Thin Layer Chromatography (TLC)

Samples of cultivation broth (1 mL) were taken every 24 h and extracted with 2 mL of ethyl acetate. The extracts were applied on TLC plates (ALUGRAM SIL G/UV254, Germany) and developed in benzene:acetone (4:1, *v*/*v*) and CHCl_3_:acetone:CH_3_COOH (50:50:0.5, *v*/*v*/*v*) for cholesterol and LCA bioconversion derivatives, respectively. Steroids with the 3-oxo-4-ene moiety were visualized under UV light (254 nm) using a hemiscope CN-15MC UV Darkroom (Vilber Lourmat, Collégien, France). To visualize cholesterol and its derivatives with the 3β-ol-5-ene configuration, the TLC plates were treated with 4% (*w*/*v*) phosphomolybdic acid hydrate solution in ethyl alcohol, followed by heating at 60–65 °C. LCA and its derivatives were assayed after staining the TLC plates with a MnCl_2_ solution [27] and heating at 105 °C for 5–10 min and visualized under UV light (365 nm).

### 2.6. High-Performance Liquid Chromatography (HPLC)

HPLC analyses were performed using reversed-phase HPLC on an Agilent Infinity 1200 system (Agilent Technologies, Germany SA) with a Symmetry column (250 × 4.6 mm, 5 μm) with a Symmetry C18 precolumn (5 μm, 3.9 × 20 mm) (Waters, Milford, MA, USA) at 50 °C and a flow rate of 1 mL/min. Steroid assays were performed using two mobile phases (acetonitrile:water:acetic acid (60:40:0.01, *v*/*v*/*v*) and acetonitrile:2-propanol:water (50:45:5, *v*/*v*/*v*)) with UV-detection at 200 nm (for compounds with the 3β-ol-5-ene configuration) and 240 nm (for compounds with the 3-oxo-4-ene configuration).

### 2.7. Mass-Spectrometry (MS), ^1^H- and ^13^C-Nuclear Magnetic Resonance Spectroscopy (^1^H- and ^13^C-NMR Spectroscopy)

MS spectra of compounds **II**, **III**, and **IV** were recorded on a tandem mass spectrometer LCQ Advantage MAX (Thermo Finnigan, Waltham, MA, USA) in the positive ion [M + H]^+^ mode at an evaporator temperature of 350 °C and capillary temperature of 170 °C. MS/MS spectra were obtained using normalized collision energy (Normolized Collision Energy^TM^) ranging from 20% to 40%. Data were collected and processed using the Xcalibur software. HRMS experiments for compounds **V**, **VI**, **VII**, and **VIII** were performed with an Orbitrap Elite mass spectrometer (Thermo Fisher Scientific GmbH, Bremen, Germany) with an ESI source.

^1^H- and ^13^C-NMR spectra were recorded at 400 and 100.6 MHz, respectively, with a Bruker Avance 400 spectrometer. Chemical shifts were measured relative to the solvent signal. Only characteristic signals are given in ^1^H-NMR of steroids.

### 2.8. Genome Analysis

Annotation of the genome was carried out using NCBI PGAP [28], RAST (http://rast.nmpdr.org/, accessed on 10 September 2019) [29,30] and KAAS (https://www.genome.jp/tools/kaas/, accessed on 10 September 2019) [31]. Orthologous and paralogous relations between genes of the *S. hirsuta* VKM Ac-666^T^, *Mycobacterium tuberculosis* H37Rv and *Rhodococcus jostii* RHA1 genomes were found using OrthoFinder 2.5.1 [32,33] with inflation parameter 1.5. A BLAST search [34] against non-redundant protein sequences (NCBI database) was used as an additional tool to confirm the predetermined enzyme function. Reciprocal BLAST was used in several cases to search for the genes that correspond to the known steroid catabolism genes one-to-one.

### 2.9. Phylogenetic Analysis

A phylogenetic dendrogram showing the relationships of KstD homologs was constructed by the maximum likelihood algorithm in MEGA7 [35]; the sequences were aligned with MUSCLE. Default parameters were used in all cases.

### 2.10. BLAST Search for Steroid Catabolism Genes

Search for the key genes of the steroid catabolic 9,10-*seco*-pathway (*kstD*, *kshA,* and *kshB)* was carried out against several dozen available genomes of thermophilic strains, using the BLAST+ program [36]. The protein sequences of KstD (NP_218054.1), KshA (NP_218043.1), and KshB (NP_218088.1) of *M. tuberculosis* H37Rv were used as reference ones. A list of bacteria to be screened (Supplementary Table S1) was compiled on the basis of the literature data [37] on thermophilic and thermotolerant actinobacteria with known complete genome sequences or annotated contigs and available sources on other known thermophilic bacteria of diverse phylogenetic positions.

The genomes of *Geobacillus kaustophilus* and *Parageobacillus thermoglucosidasius* strains capable of performing some modifications of steroid compounds were screened for the steroid catabolism genes (Supplementary Table S2) using the BLAST+ program [36].

## 3. Results

### 3.1. Cholesterol and Lithocholic Acid Bioconversion

**Figure 1:**
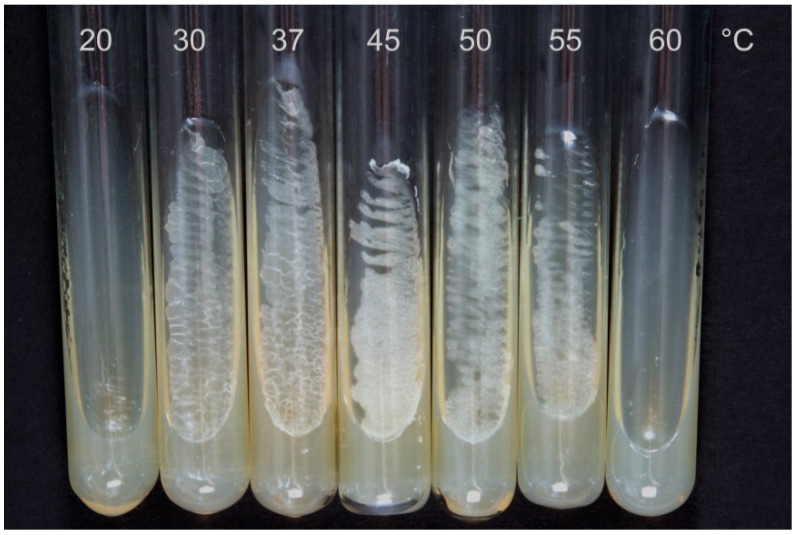
*S. hirsuta* growth at 20–60 °C for 24 h.

The *S. hirsuta* strain grew poorly at 20 °C, showed moderate growth at 30–37 °C, and grew well at 45–50 °C, but slower growth was observed at 55 °C (Figure 1).

The culture of *S. hirsuta* forms clumps during the growth making it difficult to assess growth using OD. The Table 1 shows dried biomass depending on the cultivation temperature. Further experiments were performed at a temperature of 45 °C.

As shown in Figure 2, *S. hirsuta* fully transformed cholesterol within 144 h. Intermediates were isolated, and their structures were characterized by HPLC, mass spectrometry, and ^1^H- and ^13^C-NMR-spectroscopy (Table 2, Supplementary Figures S1–S30). The intermediates were identified as 3-oxo-4-ene-compounds: cholest-4-en-3-one (**II**), cholesta-1,4-dien-3-one (**III**), 26-hydroxycholest-4-en-3-one (**IV**), 3-oxo-cholest-4-en-26-oic acid (**V**), 3-oxo-cholesta-1,4-dien-26-oic acid (**VI**), and steroids with a 3β-hydroxy-5-ene moiety: 26-hydroxycholest-5-en-3β-ol (**VII**) and 3β-hydroxycholest-5-en-26-oic acid (**VIII**).

No other steroids without a lateral chain (C_19_-steroids) or a partially oxidized side chain (C_22_- or C_24_-steroids) were detected among the intermediates. Based on the structures and the time courses of the steroids detected, the following scheme was proposed for cholesterol bioconversion with *S. hirsuta* VKM Ac-666^T^ (Figure 3).

Among the lithocholic acid bioconversion intermediates, the compounds with both the unmodified A-ring structure and the 3-keto-4-ene moiety were found (Supplementary Figure S31A,B).

### 3.2. General Clustering of Steroid Catabolic Gene Homologs

When analyzing the genome of *S. hirsuta* (DDBJ/ENA/GenBank accession no. VWPH00000000), the genes putatively involved in steroid catabolism were mainly grouped into three clusters: cluster 1 (*F1721_32550-F1721_33735*), cluster 2 (*F1721_00675-F1721_00760*) and cluster 3 (*F1721_28735-F1721_28770),* and a number of genes were revealed outside the clusters (Figure 4, Supplementary Tables S3 and S4).

Cluster 1 (Figure 4, Supplementary Tables S3 and S4) contains candidate genes related to a sterol side chain degradation pathway, A/B-ring oxidation, and the Mce4 system (operon *mceABCDEF* and the genes coding for two permease subunits YrbEa and YrbEb). In total, four *mce* loci (*F1721_29585-F1721_29620*, *F1721_32550-F1721_32585*, *F1721_10830-F1721_10865*, *F1721_13950-F1721_13915*) were found in *S. hirsuta*. The *choD*, *choE,* and *fadD3* genes, presumably encoding cholesterol oxidases and HIP-CoA synthetase, respectively, were found out of the clusters in Ac-666^T^ (Figure 4, Supplementary Tables S3 and S4).

In Ac-666^T^, clusters 2 and 3 (Figure 4, Supplementary Tables S3 and S4) contain candidate genes related to the cholate degradation pathway, namely, orthologs of the *kshA* and *kshB* subunit genes; two orthologs of *kstDs*: *kstD2* and *kstD1*; the A/B-ring opening operon *hsaEGF* and orthologs of *hsaD3* and *hsaB3*; the *ksdI* steroid delta-isomerase gene; *kstR3* for a predicted transcriptional regulator; and orthologs of the *casACEHI* genes, which determine degradation of the cholate side chain.

Figure 5 shows the scheme proposed for cholesterol bioconversion with the participation of the candidate genes of *S. hirsuta* VKM Ac-666^T^.

### 3.3. BLAST Search for the Key Enzymes of Steroid Catabolism in 52 Thermophilic/Thermotolerant Strains

The key steroid catabolism enzymes KstD, KshA, and KshB of *M. tuberculosis* H37Rv were used as reference enzymes in a BLAST search carried out against several dozen publicly available genomes of thermophilic bacteria of different phylogenetic positions (Supplementary Table S1).

Among the 52 thermophilic/thermotolerant species tested, seven actinobacterial strains were found to possess proteins of 41.6% to 64.2% similar to the *M. tuberculosis* H37Rv enzymes: *Thermomonospora curvata* DSM 43183, *Amycolatopsis granulosa* DSM 45669, *Amycolatopsis methanolica* strain 239T, *Amycolatopsis thermalba* strain 50.9b, *Thermocatellispora tengchongensis* DSM 45615, *Amycolatopsis ruanii* strain 49.3e, and *Microbispora siamensis* NBRC 104113 (Supplementary Table S5).

The genomes of two strains capable of performing some modifications of steroid compounds (i.e., 6-hydroxylation, reduction of the 17/20-keto group or 4(5)-double bond, and C17–C20 C_3_-side chain cleavage), *Geobacillus kaustophilus* and *Parageobacillus thermoglucosidasius,* were screened for the steroid catabolism enzymes ChoD, ChoL, Ltp3-4, Hsd4A, FadE26-30, ChsH1-2, FadD17, FadD19, EchA19, HsaA-E, KstD, KshAB, IpdAB, FadD3, and EchA20 (Supplementary Table S2). Most of the proteins were absent in these strains (Supplementary Table S6). On the other hand, enzymes with 47% and 45% similarity to the reference FadA5 were revealed in *G. kaustophilus* and *P. thermoglucosidasius*, respectively; and enzymes with 48% and 41% identity to HsaF and HsaE, respectively, were identified in *P. thermoglucosidasius* (Supplementary Table S6).

## 4. Discussion

Several thermophilic bacterial species have been reported to carry out distinct structural modifications of steroids [19,20,21,47], while sterol degradation by thermophilic microorganisms has not been studied so far. As shown in this research, thermophilic *S. hirsuta* transformed cholesterol (Figure 1). The cholesterol degradation pathway was predicted (Figure 4) based on the time courses of the intermediates (Figure 1) and bioinformatics analysis (Figure 4). The set and the order of the genes putatively involved in steroid catabolism in *S. hirsuta* are similar to the clusters described for *M. tuberculosis* H37Rv and *R. jostii* RHA1 [5] (Figure 4).

### 4.1. Cholesterol Oxidases (ChOs)

In many actinobacteria, the sterol degradation pathway is known to begin with the modification of 3β-hydroxy-5-ene into the 3-keto-4-ene structure by cholesterol oxidases (ChOs) or 3β-hydroxysteroid dehydrogenases (3β-HSDs) [48,49], while cytochrome P450-mediated hydroxylation at C26(27) has been reported to be the initial reaction of sterol degradation in *Rhodococcus* strains [50,51]. As evidenced from the time course of cholesterol conversion by *S. hirsuta*, the initial reactions of cholesterol degradation, i.e., modification of the 3β-ol-5-ene-moiety and sterol side chain C26(27)-hydroxylation, occurred independently (Figure 1).

ChOs are most likely involved in 3β-ol-5-ene-moiety modification in *S. hirsuta* since no candidate genes coding for 3β-HSDs were found in Ac-666^T^ [25]. Two candidate *cho* genes, *choD F1721_14655* and *choE* *F1721_09795,* were revealed in Ac-666^T^. Similar to other *cho* in actinobacteria [51], both genes are out of the steroid catabolism clusters.

### 4.2. Cyp 125

The cleavage of the cholesterol/cholestenone side chain by actinobacteria begins with hydroxylation of the terminal methyl group catalyzed by steroid 26(27)-monooxygenase to form the corresponding 26(27)-alcohols [51]. As shown for *R. jostii* RHA1, the same enzyme accounts for further oxidation to the corresponding C26-carboxylic acids [50]. Cytochrome P450 monooxygenases encoded by *cyp125* have been isolated and characterized from *M. tuberculosis* [52] and *R. jostii* RHA1 [50]. Cyp125 from *M. tuberculosis* CDC1551 has been shown to play a role in the oxidation of 26-hydroxycholest-4-en-3-one (**IV**) to cholest-4-en-3-one-26-oic acid (**V**) [53]. *Cyp125*, *cyp142*, and *cyp124* have been reported to encode the enzymes that perform terminal C(26)27-hydroxylation [51].

**Figure 2:**
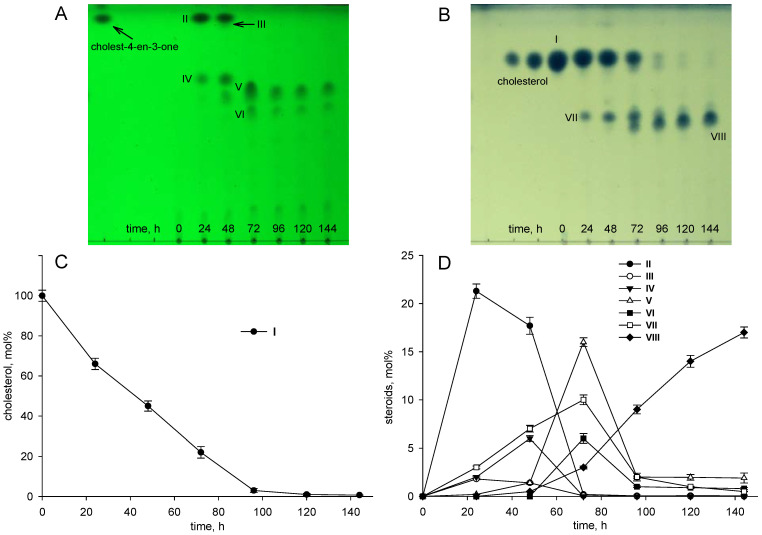
Cholesterol bioconversion by *S. hirsuta* VKM Ac-666^T^. Thin-layer chromatography (TLC) chromatogram of 3-keto-4-ene steroids (**A**), visualization under ultraviolet (UV) light (254 nm), cholest-4-en-3-one as a reference compound; TLC chromatogram of 3β-hydroxycholest-5-ene steroids (**B**), visualization after phosphomolybdic acid staining, cholesterol as a reference compound; time course of cholesterol consumption (**C**); time course of the intermediates/metabolites of cholesterol bioconversion (**D**). The data are the averages of triplicates. **I**, cholesterol (cholest-5-ene-3β-ol); **II**, cholest-4-en-3-one; **III**, cholesta-1,4-dien-3-one; **IV**, 26-hydroxycholest-4-en-3-one; **V**, 3-oxo-cholest-4-en-26-oic acid; **VI**, 3-oxo-cholesta-1,4-dien-26-oic acid; **VII**, 26-hydroxycholesterol (cholest-5-ene-3β,26-diol); **VIII**, 3β-hydroxy-cholest-5-en-26-oic acid.

**Figure 3:**
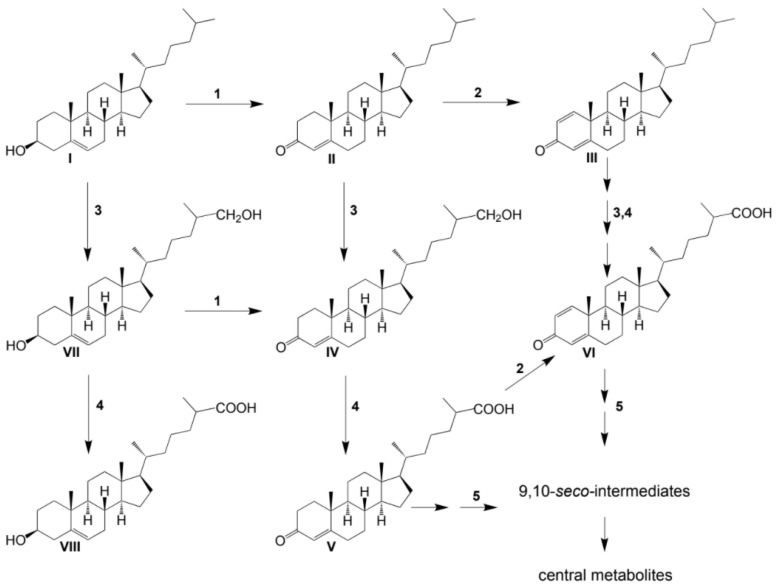
Scheme of cholesterol bioconversion by *S. hirsuta* VKM Ac-666^T^. Compounds: **I**, cholesterol; **II**, cholest-4-en-3-one; **III**, cholesta-1,4-dien-3-one; **IV**, 26-hydroxycholest-4-en-3-one; **V**, 3-oxo-cholest-4-en-26-oic acid; **VI**, 3-oxo-cholesta-1,4-dien-26-oic acid; **VII**, 26-hydroxycholesterol; **VIII**, 3β-hydroxycholest-5-en-26-oic acid. Biochemical reactions: **1**, 3β-hydroxyl group dehydrogenation and ∆^5^→∆^4^-isomerization; **2**, 3-oxo-4-ene-steroid 1(2)-dehydrogenation; **3**, C26(27)-hydroxylation; **4**, C26-alcohol hydroxylation; **5**, oxidative side-chain degradation.

**Figure 4:**
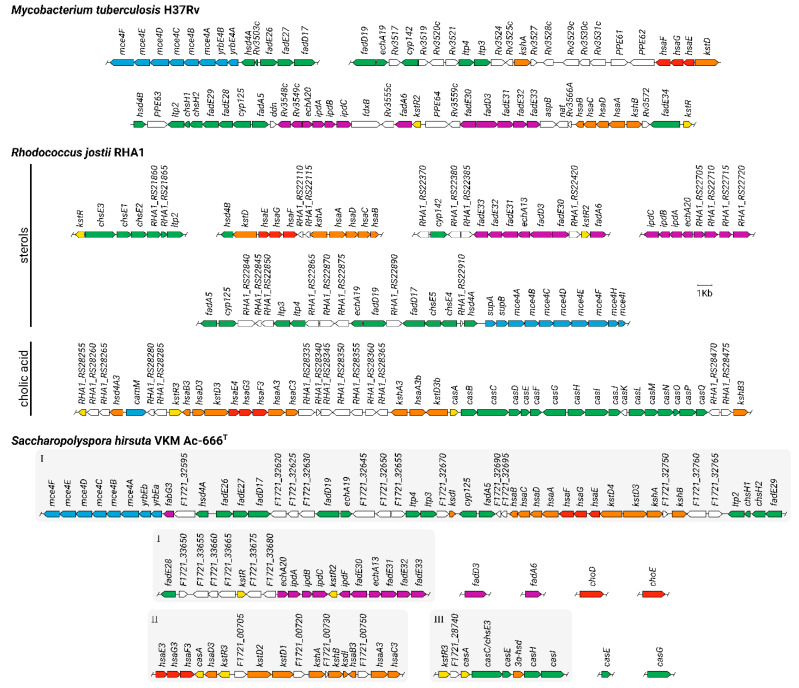
Organization of the *S. hirsuta* VKM Ac-666^T^ genes putatively involved in cholesterol and cholic acid catabolism. For comparison, the organization of the corresponding genes of *Mycobacterium tuberculosis* H37Rv and *Rhodococcus jostii* RHA1 [5] is shown. Genes related to cholesterol or bile acid side chain degradation are shown in green; genes related to A/B-rings degradation are shown red (cholesterol catabolism) and orange (cholic acid catabolism); genes coding for C/D-ring degradation are shown purple; blue color indicates genes coding for transport systems; regulatory elements are indicated yellow. I, II, and III are the *S. hirsuta* gene clusters discussed in the text.

**Figure 5:**
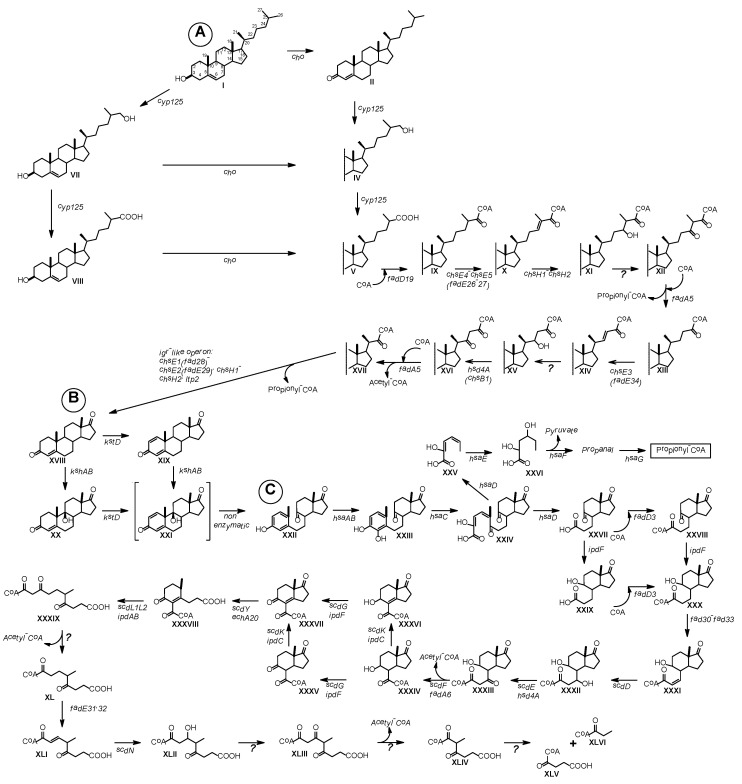
Biochemical scheme proposed for cholesterol catabolism in *S. hirsuta* VKM Ac-666^T^. Genes encoding respective proteins are denoted. (**A**) Modification of 3β-ol-5-ene to 3-keto-4-ene moiety in the A-ring of the steroid core and degradation of the sterol side chain to C_19_-steroids. (**B**) Steroid core modifications. (**C**) Steroid core degradation via the 9(10)-*seco* pathway. **I**, cholesterol; **II**, cholest-4-en-3-one; **IV**, 26-hydroxy-cholest-4-en-3-one; **V**, 3-oxo-cholest-4-en-26-oic acid; **VII**, cholest-5-ene-3β,26-diol; **VIII**, 3β-hydroxy-cholest-5-en-26-oic acid; **IX**, 3-oxo-cholest-4-en-26-oyl-CoA; X, 3-oxo-cholesta-4,24-dien-26-oyl-CoA; **XI**, 24-hydroxy-3-oxo-cholest-4-en-26-oyl-CoA; **XII**, 3,24-dioxo-cholest-4-en-26-oyl-CoA; **XIII**, 3-Oxo-chol-4-en-24-oyl-CoA; **XIV**, 3-oxo-chola-4,22-dien-24-oyl-CoA; **XV**, 22-hydroxy-3-oxo-chol-4-en-24-oyl-CoA; **XVI**, 3,22-dioxo-chol-4-en-24-oyl-CoA; **XVII**, 3-oxo-4-pregnene-20-carboxyl-CoA; **XVIII**, androst-4-ene-3,17-dione (AD); **XIX**, androsta-1,4-diene-3,17-dione (ADD); **XX**, 9α-hydroxy-AD; **XXI**, unstable 9α-hydroxy-ADD; **XXII**, 3β-hydroxy-9,10-seco-androsta-1,3,5(10)-triene-9,17-dione (3βHSA); **XXIII**, 3,4-dihydroxy-9,10-secoandrosta-1,3,5(10)-triene-9,17-dione (3,4-DHSA); **XXIV**, 4,5-9,10-diseco-3-hydroxy-5,9,17-trioxoandrosta-1(10),2-diene-4-oic acid (4,9-DSHA); **XXV**, 2-hydroxyhexa-2,4-dienoic acid (2-HHD); **XXVI**, 4-hydroxy-2-oxohexanoic acid; **XXVII**, 9,17-dioxo-1,2,3,4,10,19-hexanorandrostan-5-oic acid (DOHNAA) or 3aα-H-4α-(3′-propanoate)-7aβ-methylhexahydro-1,5-indadione (HIP); **XXVIII**, 9,17-dioxo-1,2,3,4,10,19-hexanorandrostan-5-oyl-CoA (HIP-CoA); **XXIX**, 9-hydroxy-17-oxo-1,2,3,4,10,19-hexanorandrostan-5-oic acid or 3aα-H-4α(3′-propanoate)-5α-hydroxy-7aβ-methylhexahydro-1-indanone (5-OH-HIP); **XXX**, 9-hydroxy-17-oxo-1,2,3,4,10,19-hexanorandrostan-5-oyl-CoA (5-OH-HIP-CoA); **XXXI**, 9-hydroxy-17-oxo-1,2,3,4,10,19-hexanorandrost-6-ene-5-oyl-CoA (5-OH-HIPE-CoA); **XXXII**, 7,9-Dihydroxy-17-oxo-1,2,3,4,10,19-hexanorandrostan-5-oyl-CoA; **XXXIII**, 9-hydroxy-7,17-dioxo-1,2,3,4,10,19-hexanorandrostan-5-oyl-CoA; **XXXIV**,-9-hydroxy-17-oxo-1,2,3,4,5,6,10,19-octa-norandrostan-7-oyl-CoA or 3aα-H-4α(carboxylCoA)-5α-hydroxy-7aβ-methylhexahydro-1-indanone (5-OH-HIC-CoA); XXXV, 9,17-dioxo- 1,2,3,4,5,6,10,19-octa-norandrostan-7-oyl-CoA; **XXXVI**, 9-hydroxy-17-oxo-1,2,3,4,5,6,10,19-octa-norandrost-8(14)-en-7-oyl-CoA; **XXXVII**, 9,17-dioxo-1,2,3,4,5,6,10,19-octa-norandrost-8(14)-en-7-oyl-CoA or 7a-methyl-1,5-dioxo-2,3,5,6,7,7a-hexahydro-1H-indene-4-carboxylic acid (HIEC-CoA); **XXXVIII**, 9-oxo-1,2,3,4,5,6,10,19-octanor-13,17-secoandrost-8(14)-ene-7,17-dioic acid-CoA-ester or (R)-2-(2-carboxyethyl)-3-methyl-6-oxocyclohex-1-ene-1- carboxyl-CoA (COCHEA-CoA); **XXXIX**, 6-methyl-3,7-dioxo-decane-1,10-dioic acid-CoA ester; **XL**, 4-methyl-5-oxo-octane-1,8-dioic acid-CoA ester; **XLI**, 4-methyl-5-oxo-oct-2-ene-1,8-dioic acid-CoA ester (MOODA-CoA); **XLII**, 3-hydroxy-4-methyl-5-oxo-octane-1,8-dioic acid-CoA ester; **XLIII**, 4-methyl-3,5-dioxo-octane-1,8-dioic acid-CoA ester; **XLIV**, 2-methyl-3-oxo-hexane-1,6-dioic acid-CoA ester; **XLV**, succinyl-CoA; **XLVI**, propionyl-CoA. Adopted from: [8,16,38,39,40,41,42,43,44,45,46].

The candidate *cyp125 (F1721_32680)* was identified in *S. hirsuta* (Supplementary Tables S1 and S2). Cyp125 could be responsible for the formation of 26-hydroxycholestenone (**IV**), 3-oxocholest-4-ene-26-oic acid (**V**), 3-oxocholesta-1,4-diene-26-oic acid (**VI**), 26-hydroxycholesterol (**VII**), and 3β-hydroxycholest-5-ene-26-oic acid (**VIII**) from the corresponding precursors in Ac-666^T^ (Figure 2, Figure 3 and Figure 4). Probably, this strain possesses a steroid 26(27)-monooxygenase capable of oxidizing the sterol side chain regardless of the 3β-hydroxy-5-ene- or 3-oxo-4-ene-structure of the A-ring. No orthologs of *cyp124* or *cyp142* were found in *S. hirsuta*.

### 4.3. Side Chain Degradation

As is well established for many actinobacteria, the aliphatic side chain of sterols is degraded by a cascade of reactions similar to the β-oxidation of fatty acids. The *chsE4* (*fadE26*) and *chsE5* (*fadE27*) genes of *M. tuberculosis* H37Rv have been shown to encode acyl-CoA dehydrogenases [42]. ChsE3 of *M. tuberculosis* catalyzes oxidation of 3-oxochol-4-ene-24-oil-CoA in the second round of β-oxidation of the cholesterol side chain [42]. The orthologous genes *chsE1* (*F1721_33645*), *chsE2* (*F1721_32785*)*, chsE3* (*F1721_28750*)*, chsE4* (*F1721_32605*), and *chsE5* (*F1721_32610*) were found in *S. hirsuta* (Supplementary Tables S3 and S4).

The phylogenetic analysis of acyl-CoA synthetases revealed four different types of acyl-CoA synthetases from *R. jostii* RHA1 and *M. tuberculosis* H37Rv, which are specific to the chain length of steroids [54]. FadD19 from *M. tuberculosis* H37Rv activates cholesterol metabolites with the C8-side chain, whilst FadD17 from H37Rv acts in the case of the C5- or longer side chains; and CasG from *R. jostii* RHA1, in the case of the cholate C5-side chain. Metabolites with the C3-side chain are activated by the steroid-22-oyl-CoA synthetase CasI during cholate oxidation by *R. jostii* RHA1 [54]. Orthologs of *fadD19* (*F1721_32635*), *fadD17* (*F1721_32615*), *casG* (*F1721_02405*), and *casI* (*F1721_28770*), which encode acyl-coenzyme A synthases, were revealed in *S. hirsuta* (Supplementary Tables S3 and S4). Probably, the presence of the homologous genes encoding various acyl-coenzyme A synthases in Ac-666^T^ contributes to the adaptation of the thermophilic microorganism in nature.

As shown for *R. rhodochrous* RG32, decomposition of the sterol C24-branched side chain is mediated by aldol lyases encoded by *ltp3* and *ltp4* [55]. The candidate genes *ltp3* (*F1721_32665*) and *ltp4* (*F1721_32660*) putatively involved in degrading sterols with branched side chains were identified in *S. hirsuta* (Supplementary Tables S3 and S4).

Enoyl-coenzyme A is a hydratase encoded by *echA19* that acts on 3-oxo-chol-4,22-diene-24-oyl-CoA [56]. The product of the *hsd4A* gene from *M. neoaurum* ATCC 25795 is a dual-function enzyme with both 17β-hydroxysteroid dehydrogenase and β-hydroxyacyl-CoA dehydrogenase activities [57]. Recently, it has been shown that the ChsB1 from *M. tuberculosis* (Rv3502c) is stereospecific and catalyzes the dehydrogenation of 22*S*-hydroxy-3-oxo-cholest-4-en-24-oyl-CoA rather than its 22*R* stereoisomer [46]. The candidate genes *echA19* (*F1721_32640*) and *hsd4A* (*F1721_32600*) were revealed in *S. hirsuta* (Supplementary Tables S3 and S4).

The role of thiolase FadA5 in the last cycle of cholesterol side chain β-oxidation has been demonstrated for *M. tuberculosis* H37Rv [58]. Orthologous *fadA5* (*F1721_32685*) is present in *S. hirsuta* (Supplementary Tables S3 and S4).

In *M. tuberculosis* strains, the operon containing genes for a putative lipid transfer protein (*ltp2*/*Rv3540c*), two MaoC-like hydratases (*chsH1*/*Rv3541c*, *chsH2*/*Rv3542c*), two acyl-CoA dehydrogenases (*fadE29*/*chsE2*/*Rv3543c*, *fadE28*/*chsE1*/*Rv3544c*), and cytochrome P450 (*cyp125*/*Rv3545c*) has been reported to be essential for virulence [39]. Recently, the function of Ltp2 in complex with a hydratase ChsH2_DUF35_ was identified as an aldolase in *T. curvata* DSM 43183 [59]. The orthologous genes *ltp2* (*F1721_32770*), *chsH1* (*F1721_32775*), and *chsH2* (*F1721_32780*) were found in *S. hirsuta* (Supplementary Tables S3 and S4).

### 4.4. Steroid Nucleus Degradation

The key reactions in steroid core degradation are 1(2)-dehydrogenation and 9α-hydroxylation [4]. 1(2)-Dehydrogenation is catalyzed by 3-ketosteroid Δ^1^-dehydrogenases (KstDs) [60]. The presence of several KstDs with distinct activities has been reported for actinobacterial species [60,61,62,63,64,65]. Three putative KstDs were identified in Ac-666^T^ (Supplementary Tables S3 and S4). The candidate gene *kstD3* is in cluster 1 (Figure 4). The two other candidate *kstDs, kstD2* and *kstD1,* are located side by side in cluster 2 (Figure 4). As reported earlier, *S. hirsuta* efficiently transforms androst-4-ene-3,17-dione (AD), 3β-hydroxy-5-en-17-one (DHEA), and 3β,7(α/β)-dihydroxy-5-ene-D-homo-lactones into the corresponding 1(2)-dehydrogenated derivatives, thus evidencing high KstD activity [24].

In the present study, detection of the intermediates with a 3-keto-1,4-diene structure, such as cholesta-1,4-dien-3-one (**III**) and 3-oxo-cholesta-1,4-diene-26-oic acid (**VI**), evidenced that 1(2)-dehydrogenation can take place at the early stages of sterol catabolism in *S. hirsuta* (Figure 2). As shown for *M. neoaurum* DSM 1381, KstD1, KstD2, and KstD3 catalyze 1(2)-dehydrogenation of various steroid substrates at different stages of sterol degradation [65]. The presence of several KstDs probably provides 1(2)-dehydrogenation of various steroids in *S. hirsuta*.

**Figure 6:**
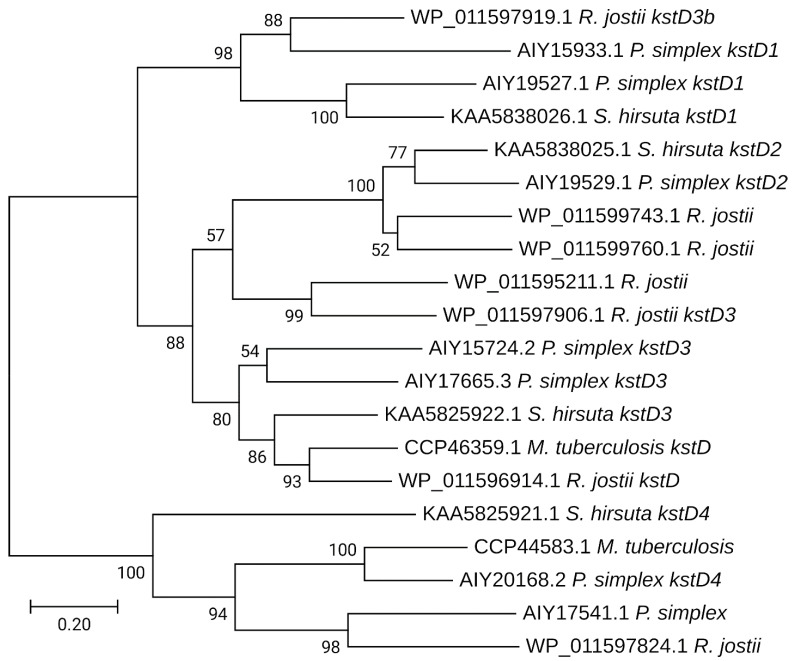
Dendrogram showing the phylogeny of KstD homologs. The tree was drawn to scale, with branch lengths measured in substitutions per site. Bootstrap values (based on 1000 replications) are indicated at the branch points.

The phylogenetic dendrogram with the KstD homologs demonstrates that KstD2 from *S. hirsuta* is in close identity with KstD2 from *N. simplex* (= *Pimelobacter simplex*) (AIY19529.1) (Figure 6). KstD from *M. tuberculosis* is in the same clade with KstD3 from *S. hirsuta*, while KstD1 from *S. hirsuta* is more similar to the corresponding enzymes of *N. simplex* (Figure 6).

9α-Hydroxylation is carried out by 3-ketosteroid 9α-hydroxylase KshAB, which consists of an oxygenase component (KshA) and a reductase component (KshB) [66]. Five different paralogous genes have been reported to encode the KshA subunits in *Mycolicibacterium fortuitum* VKM Ac-1817D (=*Mycobacterium* sp. VKM Ac-1817D) [61], thus providing for 9α-hydroxylation of steroid metabolites at various stages of sitosterol catabolism [67]. Several KshAs with different substrate specificities have similarly been found in *R. rhodochrous* DSM 43269: KshA1 was shown to participate only in the cholic acid catabolism, while KshA5 could hydroxylate several substrates [68]. Two *kshA* orthologs (*F1721_32745* and *F1721_00725*) and two *kshB* orthologs (*F1721_32755* and *F1721_00735*) were revealed in *S. hirsuta* (Figure 4, Supplementary Tables S3 and S4). Most likely, these two KshABs might differ on their substrate specificity in Ac-666^T^.

It should be noted that no C_19_-steroid intermediates, such as androstenedione, androstadienedione, testosterone, or 1(2)-dehydrotestosterone, were detected during the cholesterol transformation by *S. hirsuta*. This could be explained either by their rapid degradation to concentrations below the detection level, or by disruption of the A/B-rings in intermediates with a preserved side chain. For instance, 9,10-*seco-*steroid intermediates with partially degraded side chains form during bile acid transformation with *Rhodococcus* strains, evidencing that side chain degradation and B-ring opening occur simultaneously [69,70].

### 4.5. Steroid Core Degradation

The next step of steroid core destruction is hydroxylation of 3-hydroxy-9,10-*seco*-androst-1,3,5(10)-triene-9,17-dione at C4 by flavin-dependent monooxygenase (HsaAB), resulting in a 3,4-dihydroxy-derivative [71]. The characterization of HsaAB was performed for the monooxygenase from *M. tuberculosis* [72]. The operon *hsaBCDAFGE F1721_32700-F1721_32730* presumably involved in degrading the A-ring fragments was identified in *S. hirsuta* (Supplementary Tables S3 and S4). The candidate genes *hsaA3* (*F1721_00755*), *hsaB3* (*F1721_00745*), *hsaC3* (*F1721_00760*), and *hsaD3* (*F1721_00695*), which are orthologous to the *R. jostii* RHA1 *hsaA3B3C3D3* genes, were found in Ac-666^T^ (Figure 4, Supplementary Tables S3 and S4). The candidate genes *hsaF* and *hsaG* encode HsaF and HsaG, which putatively participate in the final stages of A-ring degradation (Supplementary Tables S3 and S4).

Degradation of the C/D-rings begins with the action of FadD3, whose physiological role has been studied in *M. tuberculosis* [41]. Unlike in *M. tuberculosis* H37Rv and *R. jostii* RHA1, in which *fadD3* encoding HIP-CoA synthetase lies in the corresponding cluster, the ortholog of *fadD3* is out of the clusters in *S. hirsuta* (Figure 4).

IpdE1(FadE30) and IpdE2 (FadE33) of *M. tuberculosis* have been shown to form a complex that catalyze the dehydrogenation of 5-OH-HIP-CoA to 5-OH-HIPE-CoA [44]. Crotonase Ech20 is responsible for the hydrolytic C-ring cleavage to yield HIEC-CoA. IpdAB hydrolytically cleaves the C-ring in the substrate COCHEA-CoA [43]. The candidate genes *ipdAB* (*F1721_33690-F1721_33695*), *ipdC* (*F1721_33700*)*, fadE30* (*F1721_33715*), *fadE33* (*F1721_33735*) and *echA20* (*F1721_33685*) (cluster 1) are presumably involved in C/D-ring degradation in *S. hirsuta* (Figure 4, Supplementary Tables S3 and S4).

The product of the opening of both of the C/D-rings is transformed by putative thiolase FadA6 to yield acetyl-CoA and 4-methyl-5-oxo-octanedioyl-CoA [5]. The last intermediate undergoes β-oxidation by acyl-CoA dehydrogenase FadE32 or the Fad31-FadE32 complex in *Mycobacterium* [43]. Finally, the β-oxidation products acetyl-CoA and 2-methyl-β-ketoadipyl-CoA are released, followed by the formation of propionyl-CoA and succinyl-CoA [5]. The orthologs of *fadE31* (*F1721_33725*) and *fadE32* (*F1721_33730*) were detected in *S. hirsuta* (Figure 4, Supplementary Tables S3 and S4).

### 4.6. Search for the Key Genes of Steroid Catabolism in the Genomes of Thermophilic/Thermotolerant Bacteria

In order to find out whether steroid degraders are widespread among thermophilic bacteria, a BLAST search for the *kstD* and *kshAB* key genes of the steroid catabolic 9,10-*seco*-pathway was performed using 52 publicly available genomes of thermophilic/thermotolerant strains (Supplementary Table S1). Only seven actinobacterial strains were identified as putative steroid degraders (Supplementary Table S5). The other thermophilic/thermotolerant strains do not contain enzymes similar to KstD and KshAB of *M. tuberculosis* H37Rv by more than 35% and, most likely, do not degrade steroids.

The thermophilic *G. kaustophilus* and *P. thermoglucosidasius* strains have been reported to provide separate reactions of steroid modification [19,20]. The BLAST search for more than 20 steroid catabolism enzymes (Supplementary Table S2) in these bacteria discovered the putative proteins that are 47% and 45% similar to the reference FadA5, respectively, and the *P. thermoglucosidasius* enzymes that are similar to HsaF and HsaE of *M. tuberculosis* H37Rv by 48% and 41%, respectively (Supplementary Table S6). FadA5 is known additionally to be involved in fatty acid β-oxidation; thus, the corresponding proteins of *G. kaustophilus* and *P. thermoglucosidasius* may not be intended for steroid catabolism. HsaEF participate in oxidation of the hydroxydiene derivative of hexanoic acid, meaning that similar enzymes do not necessarily participate in the catabolism of steroid compounds.

## 5. Conclusions

The thermophilic strain *Saccharopolyspora hirsuta* VKM Ac-666^T^ is capable of transforming various steroids [23,24]. As confirmed in this study, the strain efficiently transforms cholesterol and 26-alcohols with both 3β-ol-5-ene and 3-keto-4-ene A-ring structures being key intermediates. The genes related to sterol metabolism and cholic acid catabolism were for the first time identified in the genome of this thermophilic strain. The organization of the steroid catabolism genes is generally similar to that in other actinobacteria, with some differences related to individual genes and their grouping. Future transcriptomic and proteomic studies are of significance for a clearer understanding of the peculiarities of steroid catabolism in thermophilic actinobacteria.

The presence of key enzymes responsible for steroid core disruption was identified only in seven of 52 thermophilic bacteria of various phylogenetic positions, thus suggesting that steroid-degrading activity is not common in the thermophilic species.

The results contribute to the knowledge on the diversity of microbial steroid degraders and the features of steroid catabolism by thermophilic actinobacteria and could be useful for application in pharmaceutical and environmental steroid biotechnology.

## Figures and Tables

**Table 1:** Effect of temperature on the growth of *S. hirsuta* VKM Ac-666^T^.

	Cultivation Temperature, °C						
	20	30	37	45	50	55	60
Dried biomass *, g/L	0.12 ± 0.01	0.36 ± 0.07	1.21 ± 0.12	1.86 ± 0.21	1.73 ± 0.19	0.28 ± 0.08	0

* The duration of the growth—24 h.

**Table 2:** Steroid intermediates detected during cholesterol bioconversion by *S. hirsuta* VKM Ac-666^T^.

Number	Name and Chemical Structure (mol wt)	High-Performance Liquid Chromatography (HPLC), Mass-Spectrometry (MS), ^1^H- and ^13^C- Nuclear Magnetic Resonance Spectroscopy (^1^H- and ^13^C-NMR Spectroscopy) Data
**II**	**Cholest-4-en-3-one (384)** 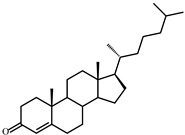	Rt (mobile phase acetonitrile:2-propanol:water 50:45:5 vol/vol/vol, λ 240 nm) 8.9 min; MS (intensity,%) [M + H]^+^: 385(100), 279(5), 226(9), 149(3), 109(4)
**III**	**Cholesta-1,4-dien-3-one (382)** 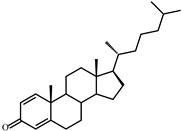	Rt (mobile phase acetonitrile:2-propanol:water 50:45:5 vol/vol/vol, λ 240 nm) 7.2 min; MS (intensity,%) [M + H]^+^ (collision energy 33 eV): 383(90), 279(5), 365(55), 325(133), 271(15), 247(100), 175(40), 163(48), 135(11), 121(8)
**IV**	**26-Hydroxycholest-4-en-3-one (400)** 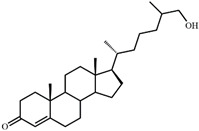	Rt (mobile phase acetonitrile:2-propanol:water 50:45:5 vol/vol/vol, λ 240 nm) 3.9 min; Rt (mobile phase acetonitrile:water:acetic acid (60:40:0.01 vol/vol/vol, λ 240 nm) 81.8 min; MS (intensity,%) [M + H]^+^ (collision energy 33 eV): 401(100), 369(33). ^1^H-NMR (CDCl_3_) δ: 5.73 (s, 1H, H-4), 1.18 (s, 3H, 19-CH_3_), 0.92 (d, *J* = 6.7 Hz, 3H, 21-CH_3_), 0.87 (d, *J* = 6.6 Hz, 3H, 26(27)-CH_3_), 0.86 (d, *J* = 6.6 Hz, 3H, 26(27)-CH_3_), 0.71 (s, 3H, 18-CH_3_)
**V**	**3-Oxo-cholest-4-en-26-oic acid (414)** 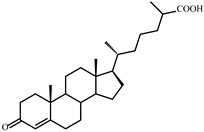	Rt (mobile phase acetonitrile:2-propanol:water 50:45:5 vol/vol/vol, λ 240 nm) 3.7 min; Rt (mobile phase acetonitrile:water:acetic acid (60:40:0.01 vol/vol/vol, λ 240 nm) 50.9 min; HRMS-ESI (*m*/*z*): [M-H]^+^ calcd for C_27_H_41_O_3_ 413,3056; found 413,3059. ^1^H-NMR (CDCl_3_) δ: 5.73 (br. s., 1H, 4-H), 1.18 (s, 3H, 19-CH_3_), 1.17 (d, *J* = 7.0 Hz, 3H, 27-CH_3_), 0.91 (d, *J* = 6.5 Hz, 3H, 21-CH_3_), 0.70 (s, 3H, 18-CH_3_). ^13^C-NMR (CDCl_3_) δ: 199.9 (C-3), 182.3 (C-26), 171.9 (C-5), 123.7 (C-4), 56.0, 55.8, 53.8, 42.4, 39.6, 39.2, 38.6, 35.63, 35.56, 33.92, 33.86, 32.9, 32.0, 28.1, 24.1, 23.6, 21.0, 18.5, 17.3, 16.7, 11.9
**VI**	**3-Oxo-cholesta-1,4-dien-26-oic (412)** 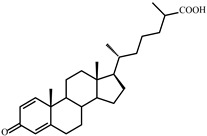	Rt (mobile phase acetonitrile:2-propanol:water 50:45:5 vol/vol/vol, λ 240 nm) 3.3 min; Rt (mobile phase acetonitrile:water:acetic acid (60:40:0.01 vol/vol/vol, λ 240 nm) 32.2 min; HRMS-ESI (*m*/*z*): [M-H]^+^ calcd for C_27_H_39_O_3_ 411,2899; found 411, 2903. ^1^H-NMR (CDCl_3_) δ: 7.06 (d, *J* = 10.1 Hz, 1H, 1-H), 6.24 (dd, *J* = 1.9, 10.1 Hz, 1H, 2-H), 6.08 (br. s., 1H. 4-H), 1.23 (s, 3H, 19-CH_3_), 1,17 (d, *J* = 7.0 Hz, 3H, 27-CH_3_), 0.91 (d, *J* = 6.5 Hz, 3H, 21-CH_3_), 0.73 (s, 3H, 18-CH_3_). ^13^C-NMR (CDCl_3_) δ: 186.6 (C-3), 182.3 (C-26), 169.8 (C-5), 156.3 (C-1), 127.4 (C-2), 123.7 (C-4), 56.0, 55.4, 52.3, 43.7, 42.6, 39.4, 39.2, 35.6, 35.5, 35.4, 33.9, 33.7, 32.9, 28.1, 24.4, 23.6, 22.8, 18.6, 18.5, 16.7, 12.0
**VII**	**26-Hydroxycholesterol** **(cholest-5-ene-3β,26-diol) (402)** 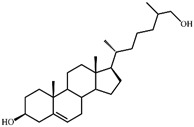	Rt (mobile phase acetonitrile:2-propanol:water 50:45:5 vol/vol/vol, λ 200 nm) 3.9 min; Rt (mobile phase acetonitrile:water:acetic acid (60:40:0.01 vol/vol/vol, λ 200 nm) 78.9 min; HRMS-ESI (*m*/*z*): [M-H]^+^ calcd for C_27_H_45_O_2_ 401,3420; found 401,3415. ^1^H-NMR (CDCl_3_) δ: 5.36 (br. s., 1H, 6-H), 3.53 (m, 1H, 3α-H), 3.51 (dd, *J* = 6.0, 10.6 Hz, 1H, CH_2_OH), 3.43 (dd, *J* = 6.4, 10.6 Hz, 1H, CH_2_OH), 1.01 (s, 3H, 19-CH_3_), 0.92 (d, *J* = 6.5 Hz, 3H, 21-CH_3_), 0.91 (d, *J* = 6.7 Hz, 3H, 27-CH_3_), 0.68 (s, 3H, 18-CH_3_). ^13^C-NMR (CDCl_3_) δ: 140.8 (C-5), 121.7 (C-6), 71.8 (C-3), 68.5 (C-26), 56.8, 56.1, 50.1, 42.32, 42.28, 39.8, 37.2, 36.5, 36.1, 35.8, 35.7, 33.5, 31.9, 31.7, 28.2, 24.3, 23.4, 21.1, 19.4, 18.7, 16.5, 11.9
**VIII**	**3β-Hydroxy-cholest-5-en-26-oic acid (416)** 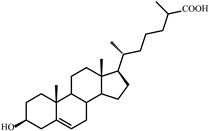	Rt (mobile phase acetonitrile:2-propanol:water 50:45:5 vol/vol/vol, λ 200 nm) 3.6 min; Rt (mobile phase acetonitrile:water:acetic acid (60:40:0.01 vol/vol/vol, λ 200 nm) 45.9 min; HRMS-ESI (*m*/*z*): [M-H]^+^ calcd for C_27_H_43_O_3_ 415,3212; found 415,3217. ^1^H-NMR (CD_3_OD) δ: 5.33 (d, *J* = 5.2 Hz, 1H, 6-H), 3.39 (m, 1H, 3α-H), 1.12 (d, *J* = 7.0 Hz, 3H, 27-CH_3_), 1.01 (s, 3H, 19-CH_3_), 0.93 (d, *J* = 6.5 Hz, 3H, 21-CH_3_), 0.71 (s, 3H, 18-CH_3_). ^13^C-NMR (CD_3_OD) δ: 180.8 (C-26), 142.2 (C-5), 122.5 (C-6), 72.4 (C-3), 58.2, 57.5, 51.7, 43.5, 43.0, 41.2, 40.7, 38.6, 37.7, 37.0, 35.4, 33.3, 33.0, 32.3, 29.3, 25.3, 24.8, 22.2, 19.9, 19.2, 17.6, 12.4

## Data Availability

Not applicable.

## Supplementary Materials

The following are available online at https://www.mdpi.com/article/10.3390/microorganisms9122554/s1, Supplementary Figures S1–S31: HPLC-, MS- and NMR- supplementary data, Supplementary Table S1: List of thermophilic and thermotolerant bacteria to be screened for the key genes of the steroid catabolic 9,10-*seco*-pathway, Supplementary Table S2: List of enzymes of steroid catabolism for BLAST+ search, Supplementary Table S3: Candidate genes for steroid or bile acid degradation in *S. hirsuta* VKM Ac-666^T^, Supplementary Table S4: List of homologous genes between *S. hirsuta* VKM Ac-666^T^, *R. jostii* RHA1, and *M. tuberculosis* H37Rv, Supplementary Table S5: List of actinobacterial strains with the similar proteins to the reference KshA, KshB, KstD of *M. tuberculosis* H37Rv, Supplementary Table S6: Result of BLAST search for 29 protein sequences of *M. tuberculosis* steroid catabolism enzymes in the *Geobacillus kaustophilus* HTA426 and *Parageobacillus thermoglucosidasius* DSM 2542 genomes.
