# Supplementary material for: Steroid Metabolism in Thermophilic Actinobacterium Saccharopolyspora hirsuta VKM Ac-666T
Source: Microorganisms. 2021 Dec 10;9(12):2554. doi: 10.3390/microorganisms9122554 (PMC8708139; doi:10.3390/microorganisms9122554)
Supplement: Supplementary file 1 [file microorganisms-09-02554-s001.zip › Supplementary Figures S1-31.pdf]

Supporting materials

article title: Steroid Metabolism in Thermophilic Actinobacteria *Saccharopolyspora hirsuta* VKM Ac-666[T]

journal name: Microorganisms

author names: Tatyana Lobastova, Victoria Fokina, Sergey Tarlachkov, Andrey Shutov, Eugeny Bragin, Alexey Kazantsev, Marina Donova

***Correspondence:** 2vvfokina@gmail.com

Table of contents

No.	Figure Capture	Page
S1	Figure S1 HPLC-profile (mobile phase acetonitrile:2-propanol:water (50:45:5 v/v/v/)) of cholesterol (standard), λ (200 nm)	5
S2	Figure S2 HPLC-profile (mobile phase acetonitrile:2-propanol:water (50:45:5 v/v/v/)) of cholest-4-en-3-one (cholestenone) (standard), λ (240 nm)	6
S3	Figure S3 HPLC-profile (mobile phase acetonitrile:2-propanol:water (50:45:5 v/v/v/)) of the individual compound isolated from the transformation media at the cholesterol conversion by *S.hirsuta*: cholest-4-en-3-one (II), λ (240 nm)	7
S4	Figure S4 HPLC-profile (mobile phase acetonitrile:2-propanol:water (50:45:5 v/v/v/)) of the individual compound isolated from the transformation media at the cholesterol conversion by *S.hirsuta*: cholesta-1,4-dien-3-one (III), λ (240 nm)	8

S5	Figure S5 HPLC-profile (mobile phase acetonitrile:2-propanol:water (50:45:5 v/v/v/)) of the individual compound isolated from the transformation media at the cholesterol conversion by *S.hirsuta*: 26-hydroxycholest-4-en-3-one (IV), λ (240 nm)	9
S6	Figure S6 HPLC-profile (mobile phase acetonitrile:2-propanol:water (50:45:5 v/v/v/)) of the individual compound isolated from the transformation media at the cholesterol conversion by *S.hirsuta*: 3-oxo-cholest-4-en-26-oic acid (V), λ (240 nm)	10
S7	Figure S7 HPLC-profile (mobile phase acetonitrile:2-propanol:water (50:45:5 v/v/v/)) of the individual compound isolated from the transformation media at the cholesterol conversion by *S.hirsuta*: 3-oxo-cholesta-1,4-dien-26-oic acid (VI), λ (240 nm)	11
S8	Figure S8 HPLC-profile (mobile phase acetonitrile:2-propanol:water (50:45:5 v/v/v/)) of the individual compound isolated from the transformation media at the cholesterol conversion by *S.hirsuta*: 26-hydroxycholesterol (VII), λ (200 nm)	12
S9	Figure S9 HPLC-profile (mobile phase acetonitrile:2-propanol:water (50:45:5 v/v/v/)) of the individual compound isolated from the transformation media at the cholesterol conversion by *S.hirsuta*: 3β-hydroxycholest-5-en-26-oic acid (VIII), λ (200 nm)	13
S10	Figure S10 HPLC-profile (mobile phase acetonitrile:water:acetic acid (60:40:0.01 v/v/v/)) of the individual compound isolated from the transformation media at the cholesterol conversion by *S.hirsuta*: 26-hydroxycholest-4-en-3-one (IV), λ (240 nm)	14

S11	Figure S11 HPLC-profile (mobile phase acetonitrile:water:acetic acid (60:40:0.01 v/v/v/)) of the individual compound isolated from the transformation media at the cholesterol conversion by *S.hirsuta*: 3-oxo-cholest-4-en-26-oic acid (V), λ (240 nm)	15
S12	Figure S12 HPLC-profile (mobile phase acetonitrile:water:acetic acid (60:40:0.01 v/v/v/)) of the individual compound isolated from the transformation media at the cholesterol conversion by *S.hirsuta*: 3-oxo-cholesta-1,4-dien-26-oic acid (VI), λ (240 nm)	16
S13	Figure S13 HPLC-profile (mobile phase acetonitrile:water:acetic acid (60:40:0.01 v/v/v/)) of the individual compound isolated from the transformation media at the cholesterol conversion by *S.hirsuta*: 26-hydroxycholesterol (VII), λ (200 nm)	17
S14	Figure S14 HPLC-profile (mobile phase acetonitrile:water:acetic acid (60:40:0.01 v/v/v/)) of the individual compound isolated from the transformation media at the cholesterol conversion by *S.hirsuta*: 3β-hydroxycholest-5-en-26-oic acid (VIII), λ (200 nm)	18
S15	Figure S15 Mass spectrum $[M+H]^+$ of cholest-4-en-3-one (384) (II)	19
S16	Figure S16 Mass spectrum $[M+H]^+$ of cholesta-1,4-dien-3-one (382) (III), (collision energy 33 eV)	19
S17	Figure S17 Mass spectrum $[M+H]^+$ of 26-hydroxy-cholest-4-en-3-one (400) (IV)	20
S18	Figure S18 Mass spectrum $[M-H]^+$ of 3-oxo-cholest-4-en-26-oic acid (414) (V)	21
S19	Figure S19 Mass spectrum $[M-H]^+$ of 3-oxo-cholesta-1,4-dien-26-oic acid (412) (VI)	22
S20	Figure S20 Mass spectrum $[M-H]^+$ of 26-hydroxycholesterol (402) (VII)	23

S21	Figure S21 Mass spectrum $[M-H]^+$ of 3β-hydroxycholest-5-en-26-oic acid (416) (VIII)	24
S22	Figure S22 ^{1}H-NMR spectrum of 26-hydroxycholest-4-en-3-one (IV)	25
S23	Figure S23 ^{1}H-NMR spectrum of 3-oxo-cholest-4-en-26-oic acid (V)	26
S24	Figure S24 ^{13}C-NMR spectrum of 3-oxo-cholest-4-en-26-oic acid (V)	27
S25	Figure S25 ^{1}H-NMR spectrum of 3-oxo-cholesta-1,4-dien-26-oic acid (VI)	28
S26	Figure S26 ^{13}C-NMR spectrum of 3-oxo-cholesta-1,4-dien-26-oic acid (VI)	29
S27	Figure S27 ^{1}H-NMR spectrum of 26-hydroxycholesterol (VII)	30
S28	Figure S28 ^{13}C-NMR spectrum of 26-hydroxycholesterol (VII)	31
S29	Figure S29 ^{1}H-NMR spectrum of 3β-hydroxycholest-5-en-26-oic acid (VIII)	32
S30	Figure S30 ^{13}C-NMR spectrum of 3β-hydroxycholest-5-en-26-oic acid (VIII)	33
S31	Figure S31 Bioconversion of lithocholic acid by *S.hirsuta* VKM Ac-666^T under the conditions used	34

Figure S1. HPLC-profile (mobile phase acetonitrile:2-propanol:water (50:45:5 v/v/v/))

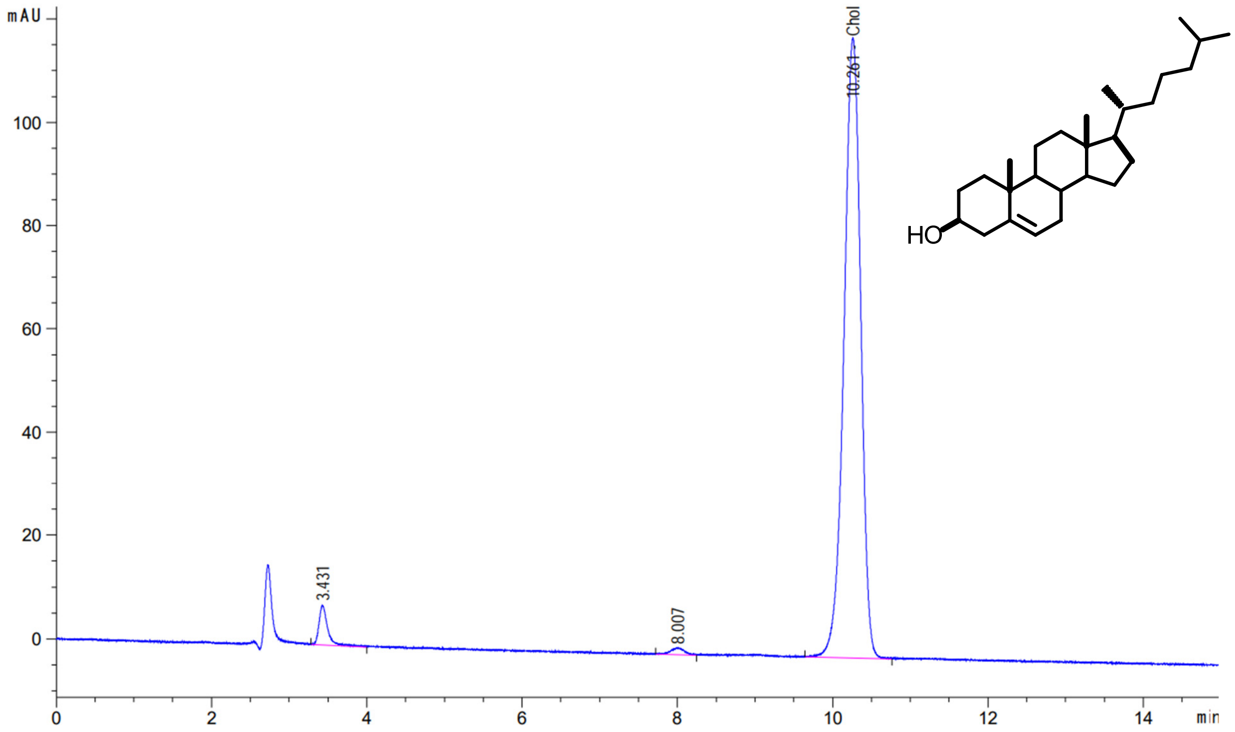

Cholesterol (standard), λ(200 nm)

Figure S2. HPLC-profile (mobile phase acetonitrile:2-propanol:water (50:45:5 v/v/v/))

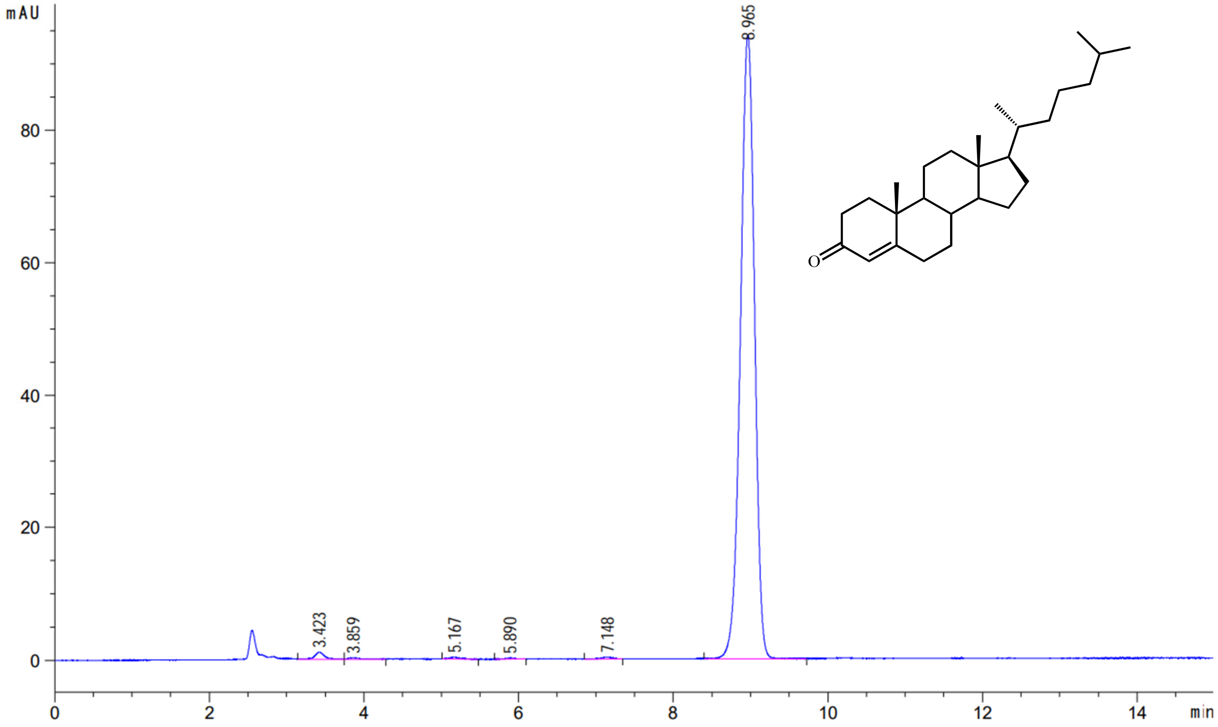

Cholest-4-en-3-one (cholestenone) (standard), λ (240 nm)

Figure S3. HPLC-profile (mobile phase acetonitrile:2-propanol:water (50:45:5 v/v/v/))

of the individual compound isolated from the transformation media at the cholesterol conversion by *S.hirsuta*

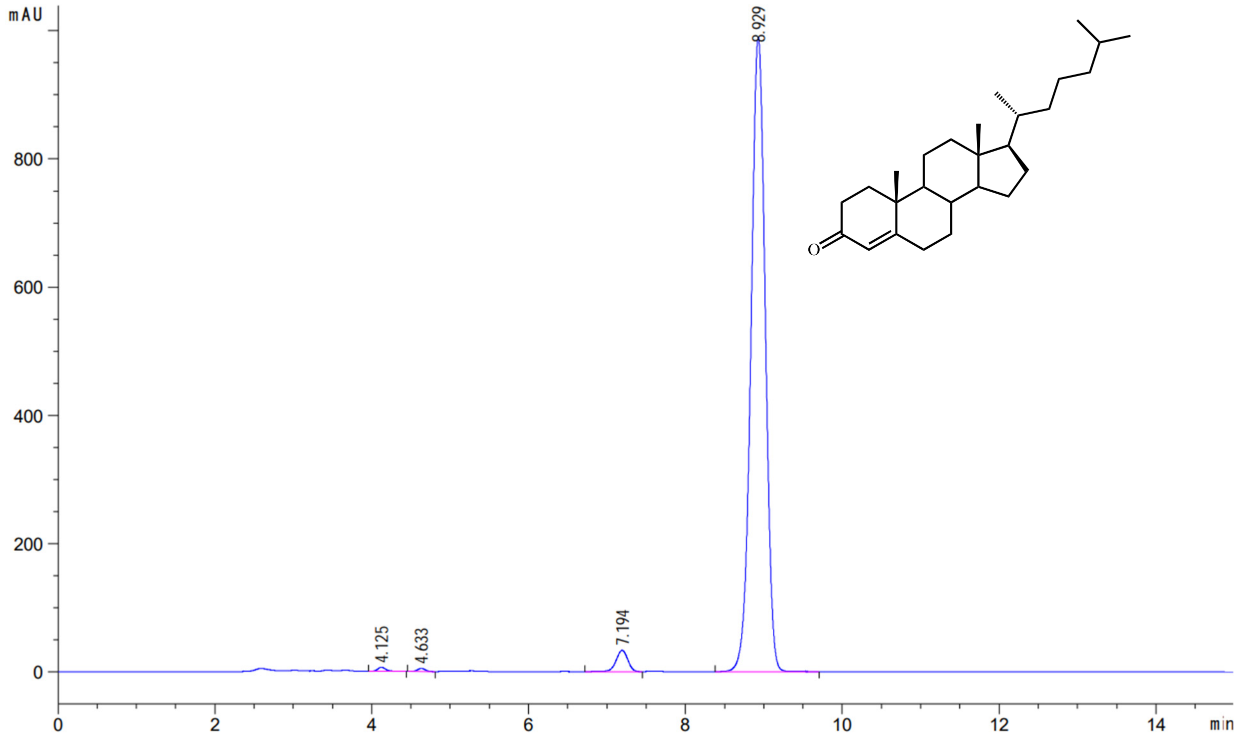

Cholest-4-en-3-one (**II**), λ (240 nm)

Figure S4. HPLC-profile (mobile phase acetonitrile:2-propanol:water (50:45:5 v/v/v/))

of the individual compound isolated from the transformation media at the cholesterol conversion by *S.hirsuta*

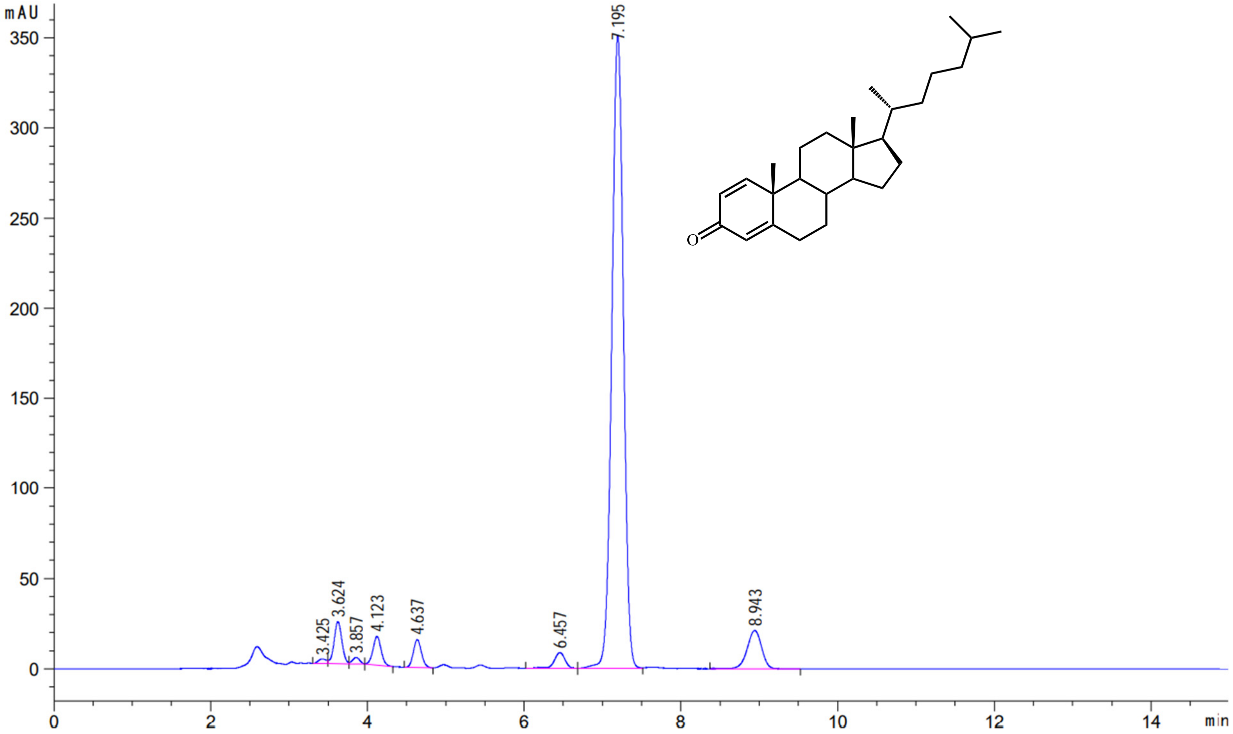

Cholesta-1,4-dien-3-one (**III**), λ (240 nm)

Figure S5. HPLC-profile (mobile phase acetonitrile:2-propanol:water (50:45:5 v/v/v/))

of the individual compound isolated from the transformation media at the cholesterol conversion by *S.hirsuta*

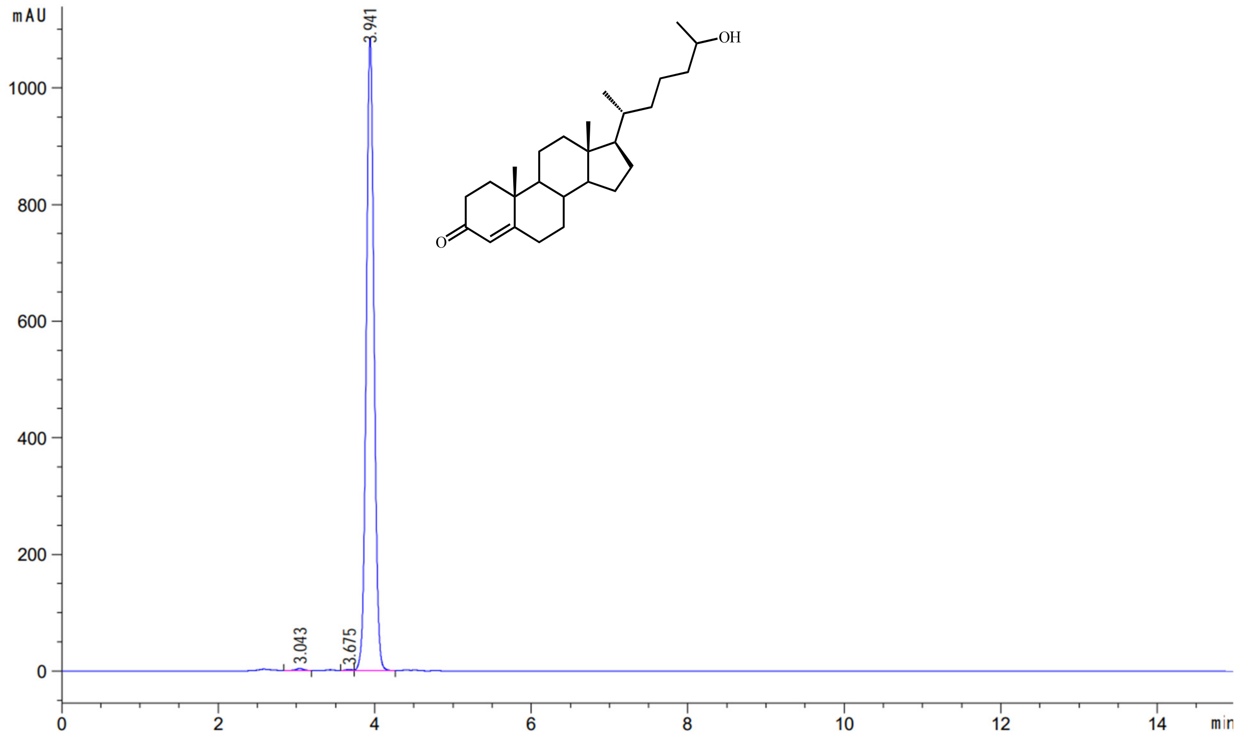

26-Hydroxycholest-4-en-3-one (**IV**), λ (240 nm)

Figure S6. HPLC-profile (mobile phase acetonitrile:2-propanol:water (50:45:5 v/v/v/))

of the individual compound isolated from the transformation media at the cholesterol conversion by *S.hirsuta*

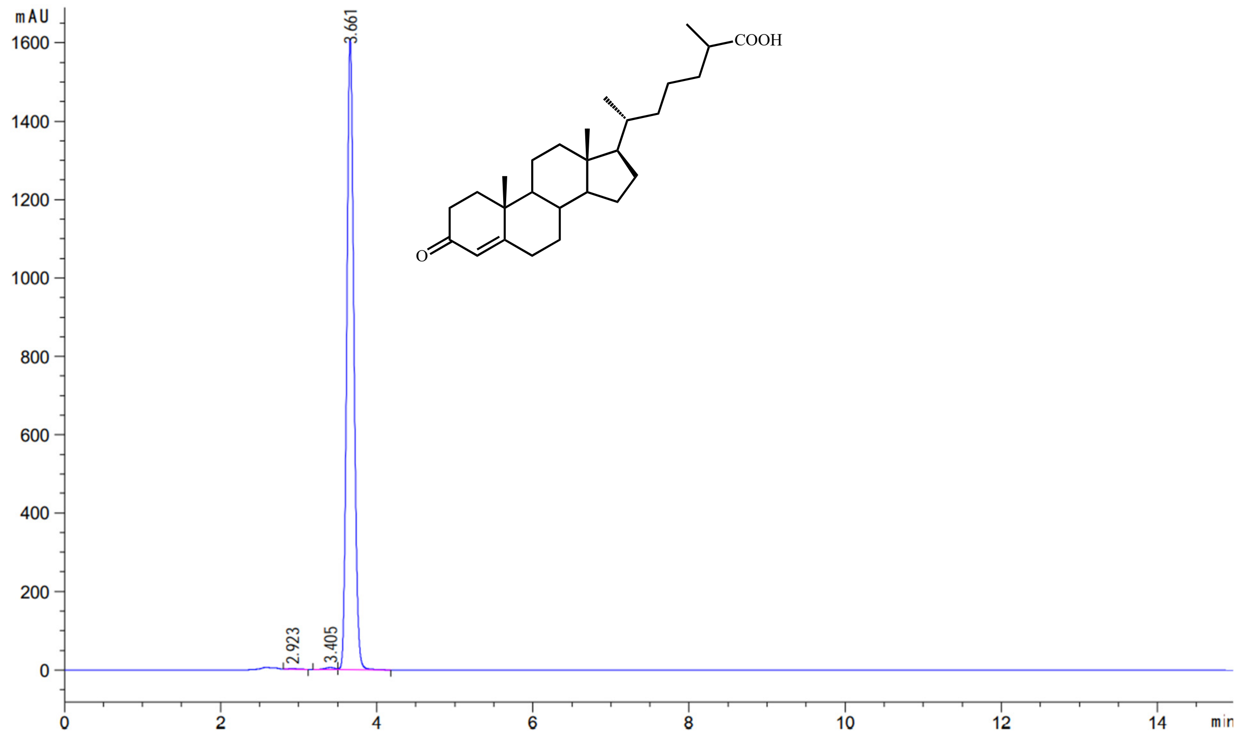

3-Oxo-cholest-4-en-26-oic acid (**V**), λ (240 nm)

Figure S7. HPLC-profile (mobile phase acetonitrile:2-propanol:water (50:45:5 v/v/v/))

of the individual compound isolated from the transformation media at the cholesterol conversion by *S.hirsuta*

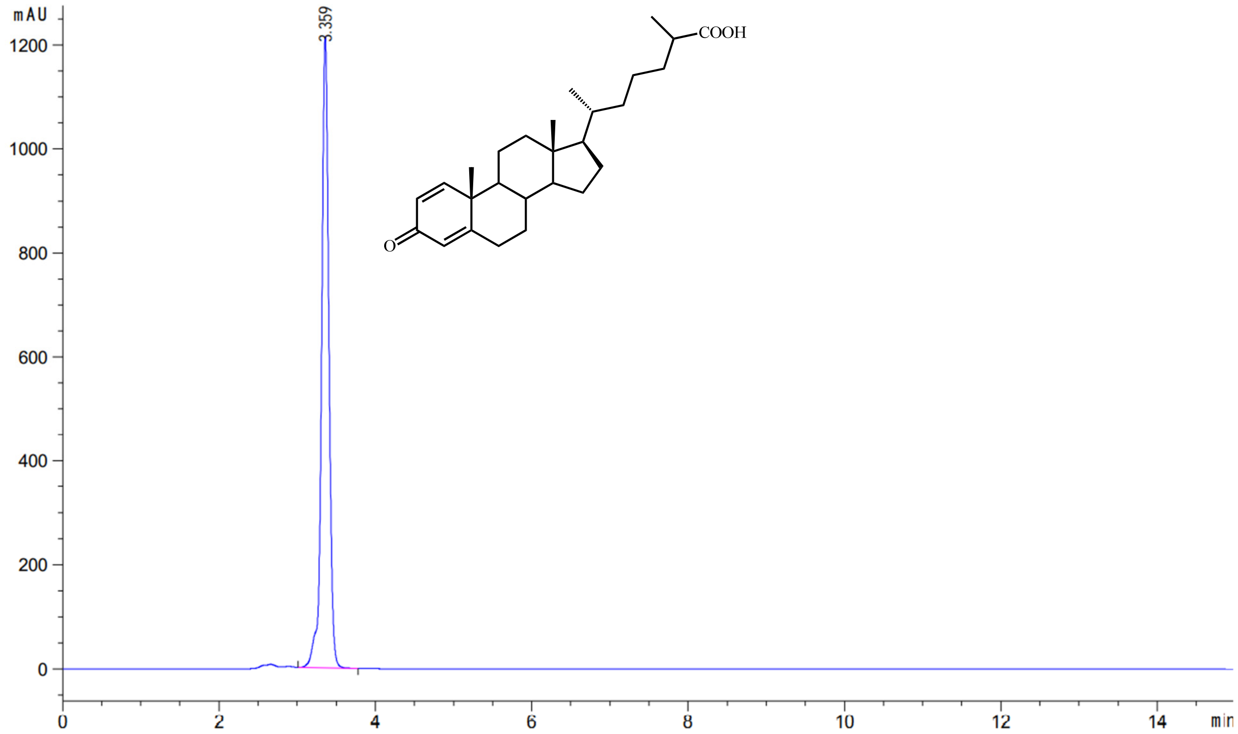

3-Oxo-cholesta-1,4-dien-26-oic acid (**VI**), λ (240 nm)

Figure S8. HPLC-profile (mobile phase acetonitrile:2-propanol:water (50:45:5 v/v/v/))

of the individual compound isolated from the transformation media at the cholesterol conversion by *S.hirsuta*

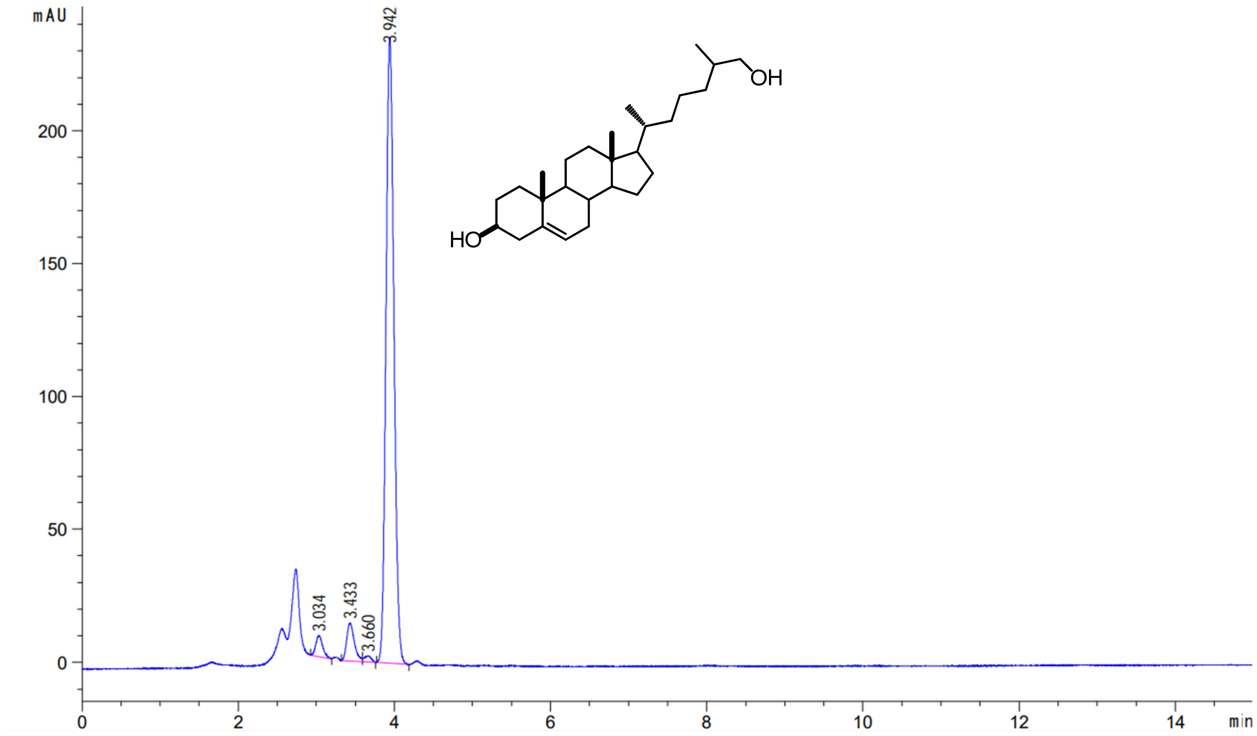

26-Hydroxycholesterol (**VII**), λ (200 nm)

Figure S9. HPLC-profile (mobile phase acetonitrile:2-propanol:water (50:45:5 v/v/v/)) of the individual compound isolated from the transformation media at the cholesterol conversion by *S.hirsuta*

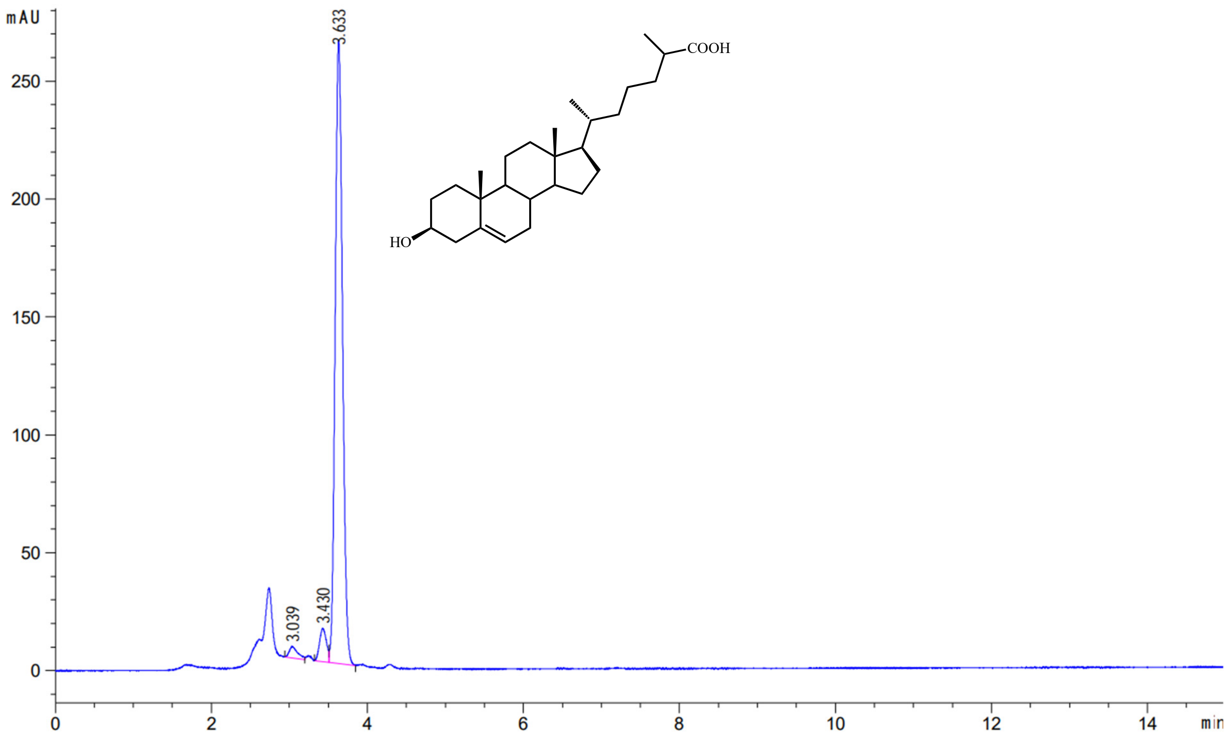

3β-Hydroxycholest-5-en-26-oic acid (**VIII**), λ (200 nm)

Figure S10. HPLC-profile (mobile phase acetonitrile:water:acetic acid (60:40:0.01 v/v/v/))

of the individual compound isolated from the transformation media at the cholesterol conversion by *S.hirsuta*

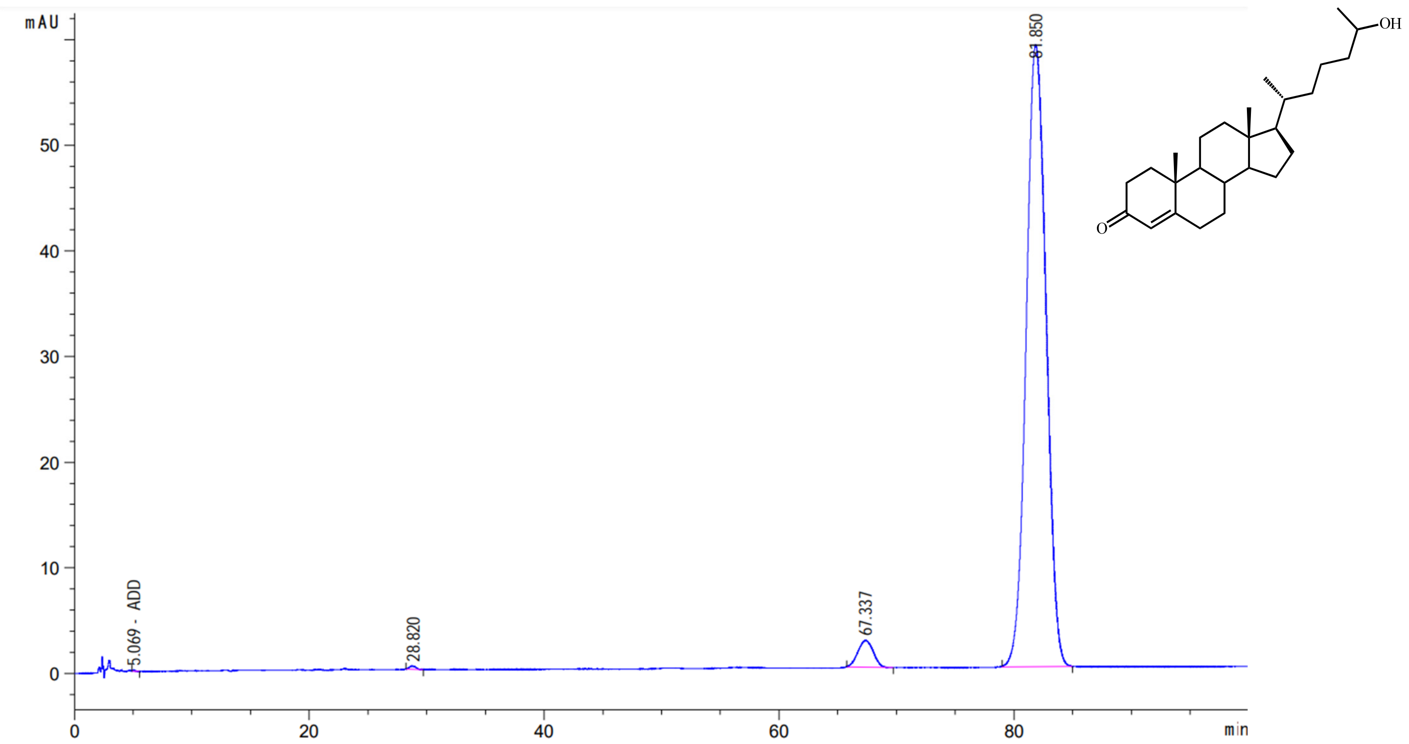

26-Hydroxycholest-4-en-3-one (**IV**), λ (240 nm)

Figure S11. HPLC-profile (mobile phase acetonitrile:water:acetic acid (60:40:0.01 v/v/v/))

of the individual compound isolated from the transformation media at the cholesterol conversion by *S.hirsuta*

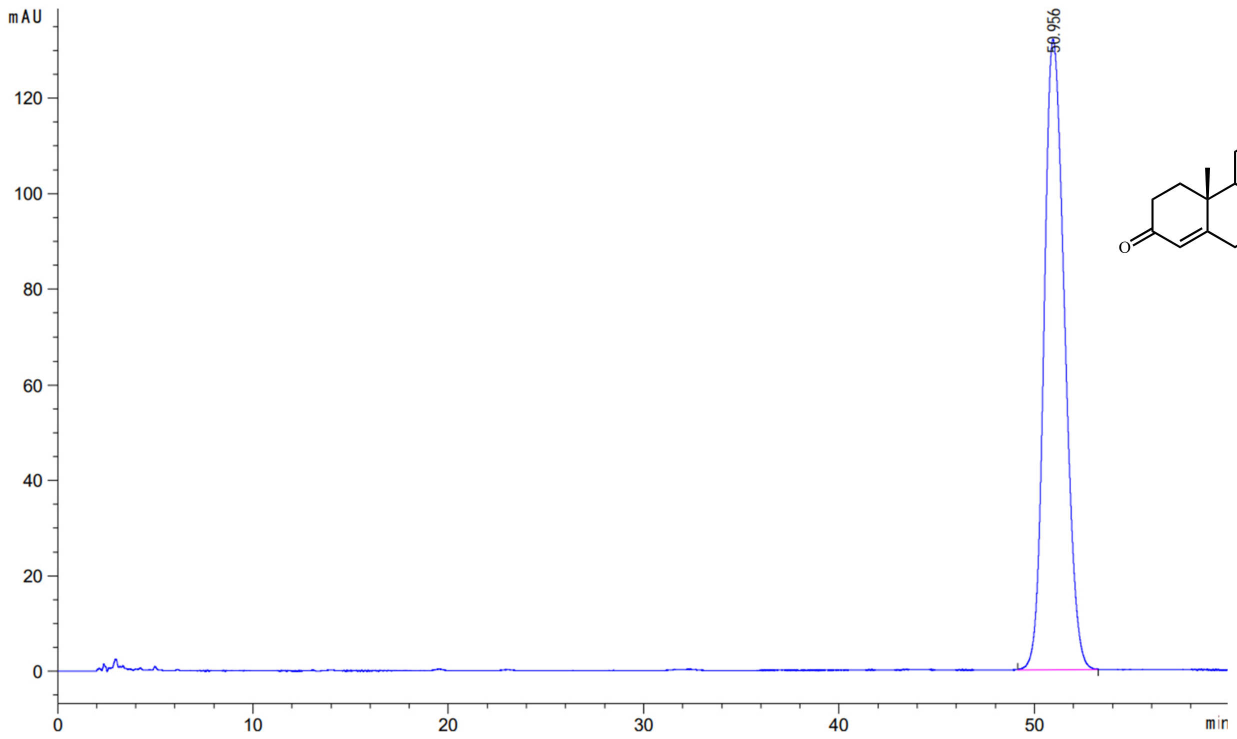

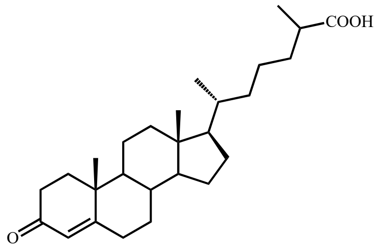

3-Oxo-cholest-4-en-26-oic acid (**V**), λ (240 nm)

Figure S12. HPLC-profile (mobile phase acetonitrile:water:acetic acid (60:40:0.01 v/v/v/))

of the individual compound isolated from the transformation media at the cholesterol conversion by *S.hirsuta*

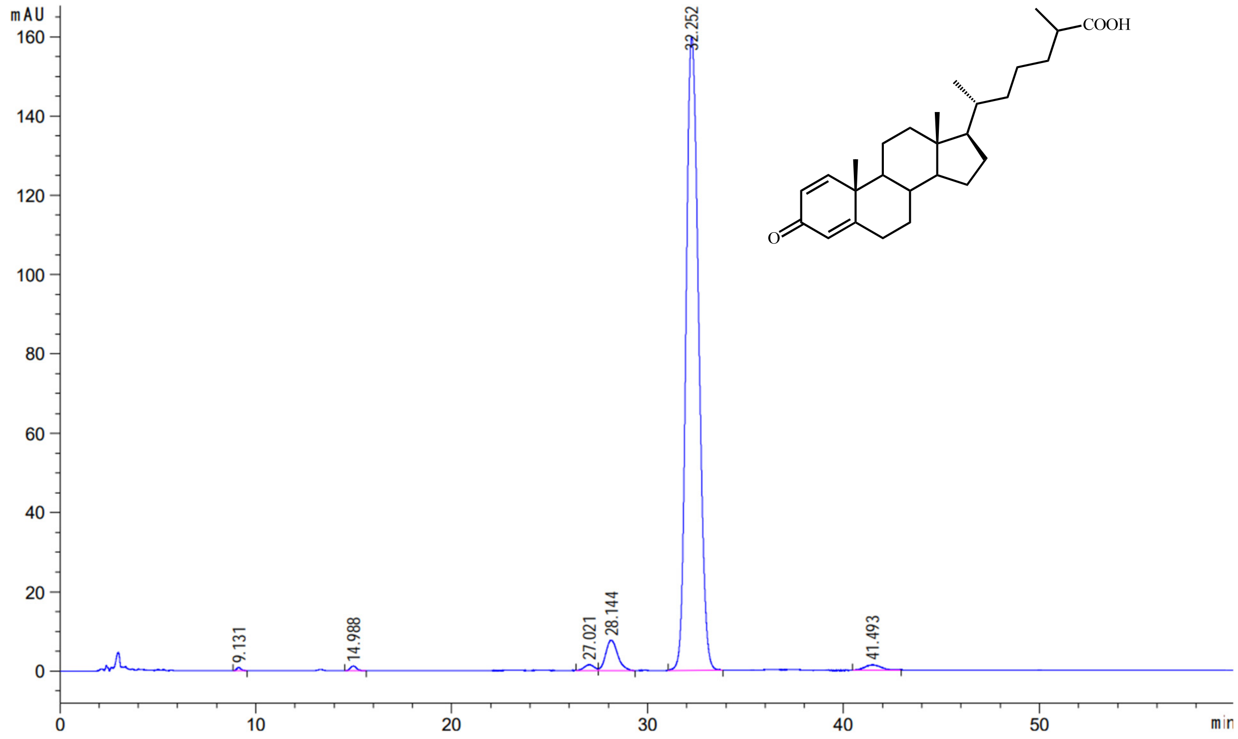

3-Oxo-cholesta-1,4-dien-26-oic acid (**VI**), λ (240 nm)

Figure S13. HPLC-profile (mobile phase acetonitrile:water:acetic acid (60:40:0.01 v/v/v/))

of the individual compound isolated from the transformation media at the cholesterol conversion by *S.hirsuta*

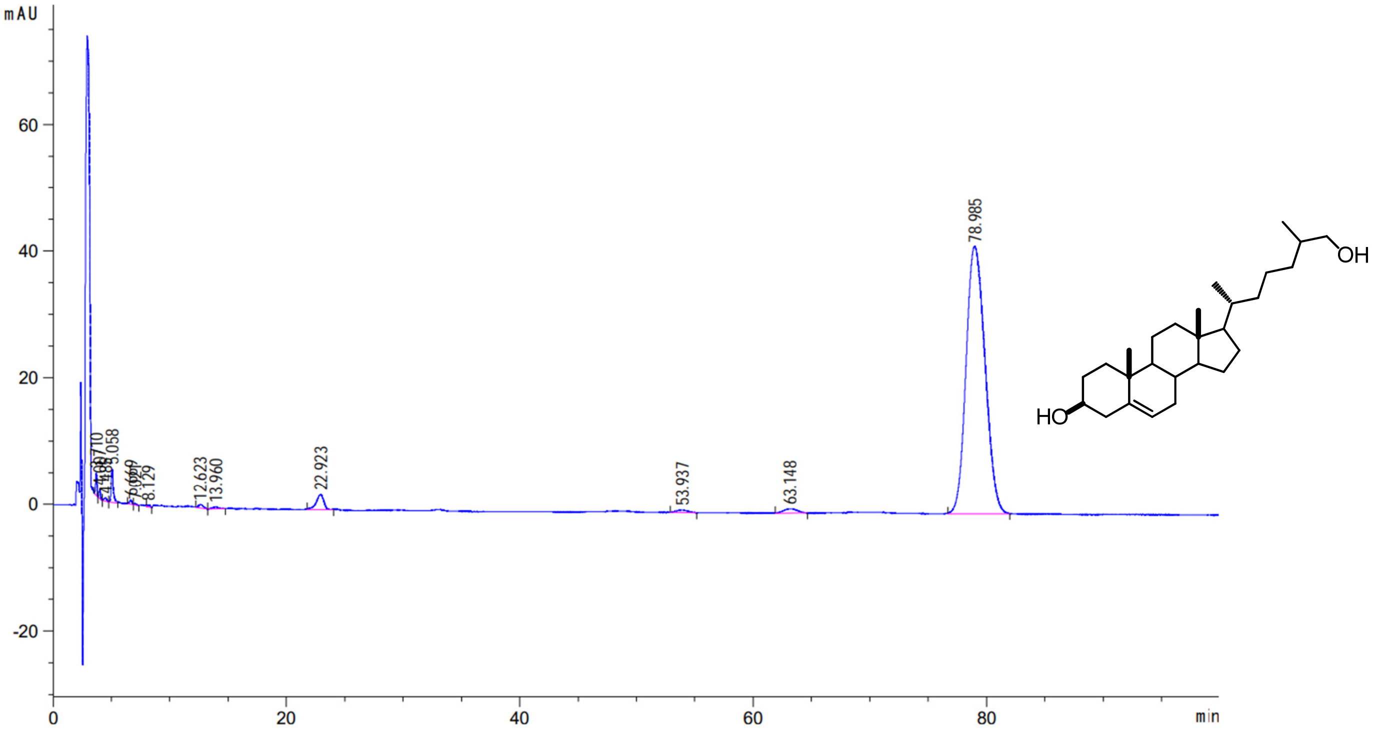

26-Hydroxycholesterol (**VII**), λ (200 nm)

Figure S14. HPLC-profile (mobile phase acetonitrile:water:acetic acid (60:40:0.01 v/v/v/))

of the individual compound isolated from the transformation media at the cholesterol conversion by *S.hirsuta*

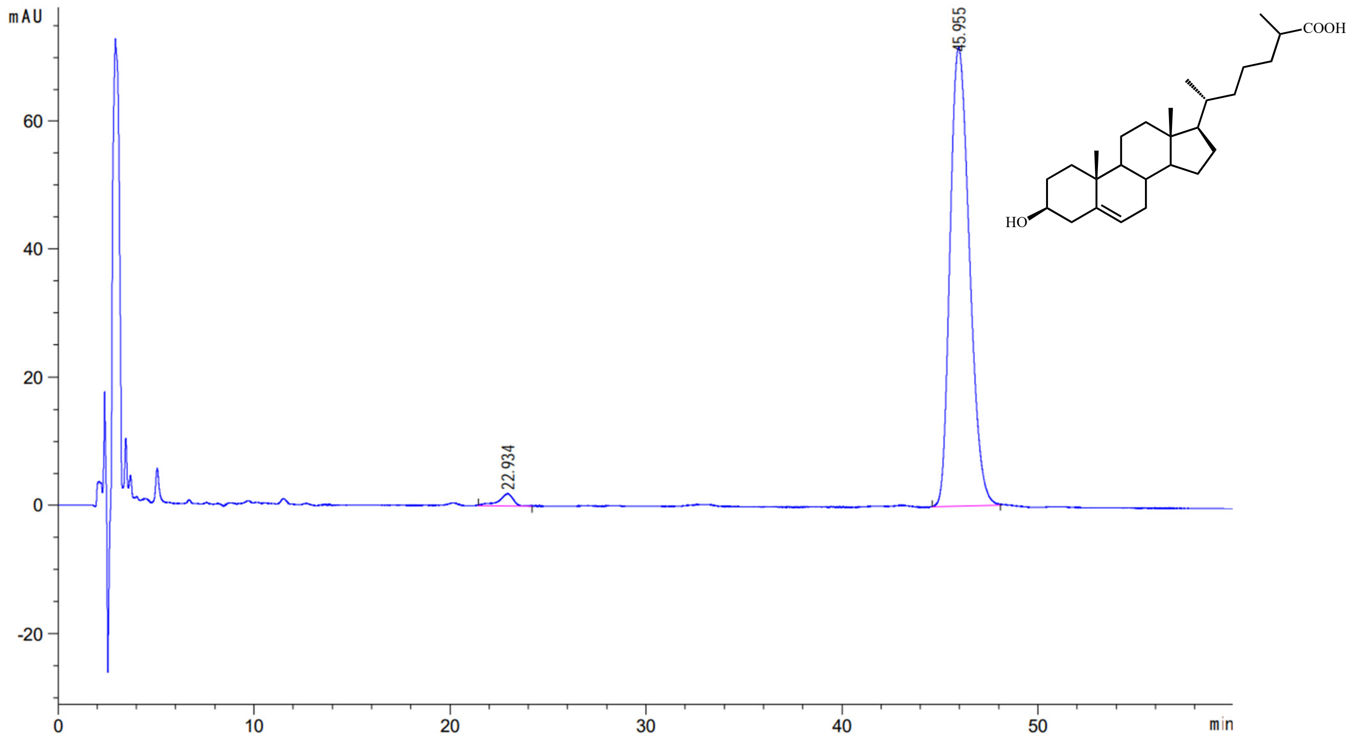

3β-Hydroxycholest-5-en-26-oic acid (**VIII**), λ (200 nm)

Figure S15. Mass spectrum [M+H]+

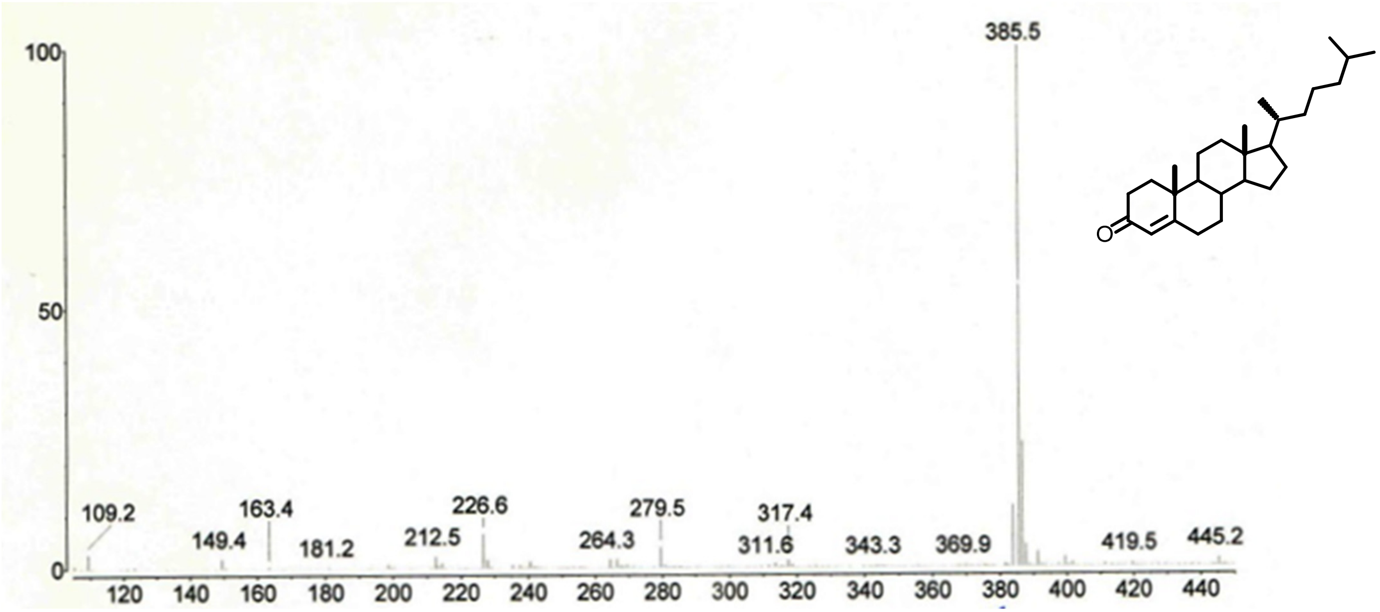

Cholest-4-en-3-one (**384**) (**II**)

Figure S16. Mass spectrum [M+H]+

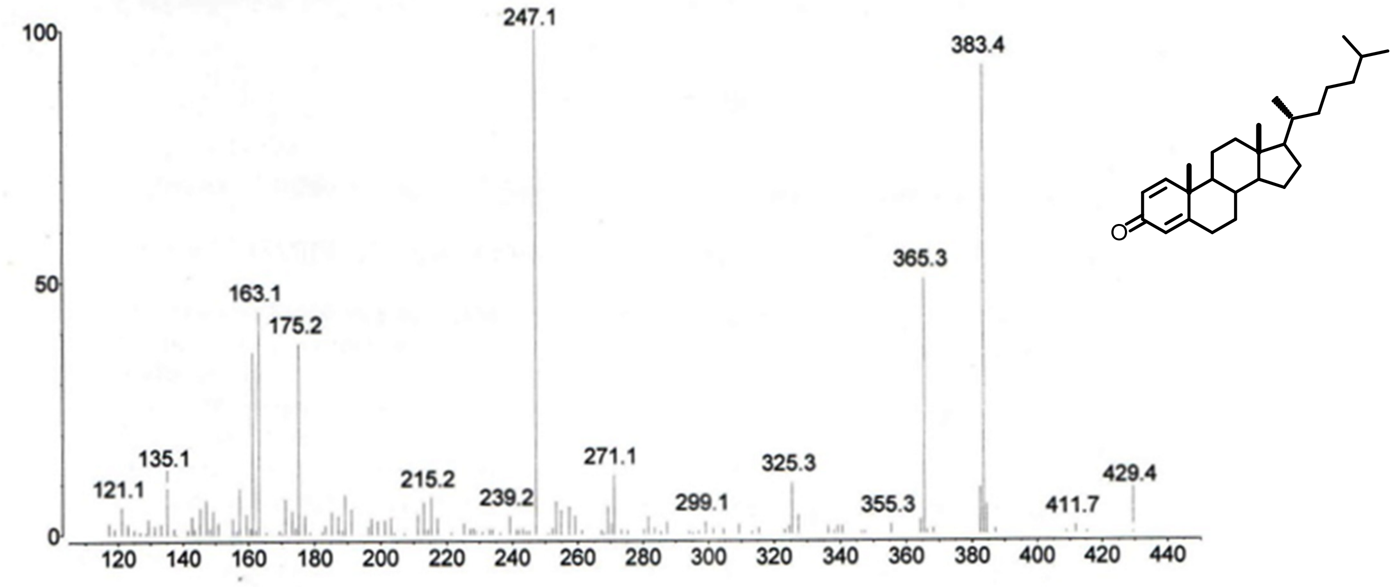

Cholesta-1,4-dien-3-one (**382**) (**III**), (collision energy 33 eV)

Figure S17. Mass spectrum [M+H]$^+$

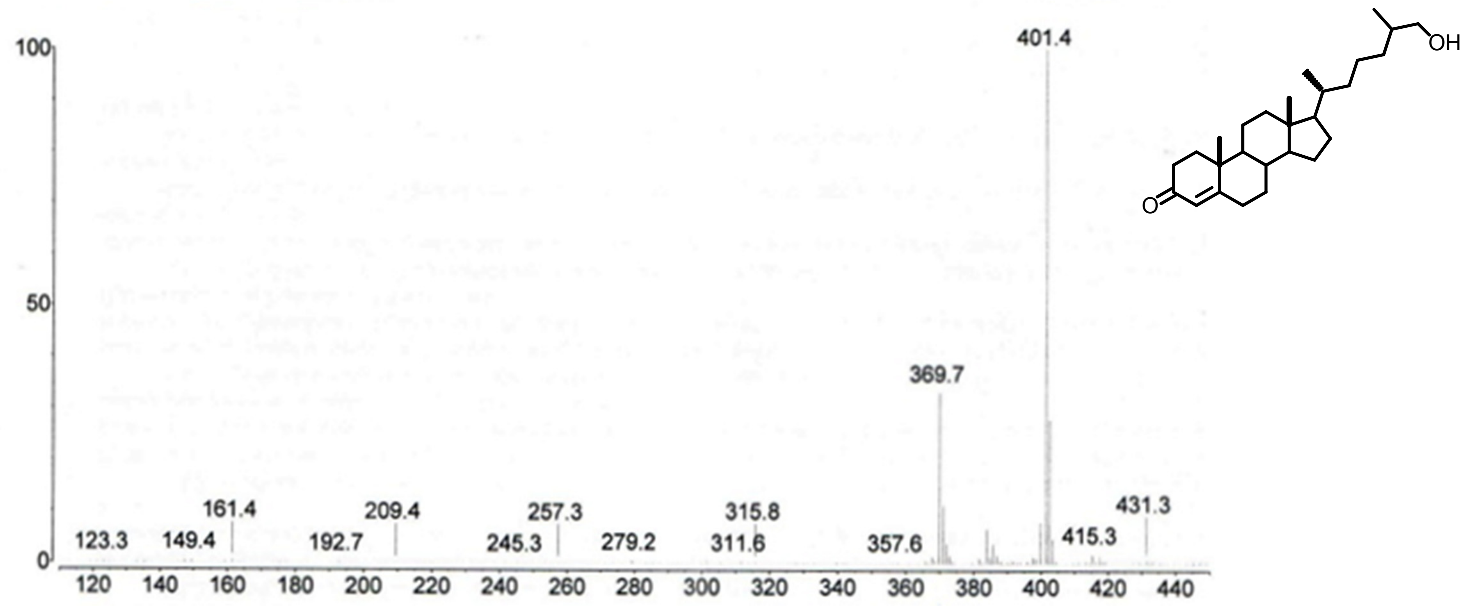

26-Hydroxy-cholest-4-en-3-one (**400**) (**IV**)

Figure S18. Mass spectrum [M-H]$^+$

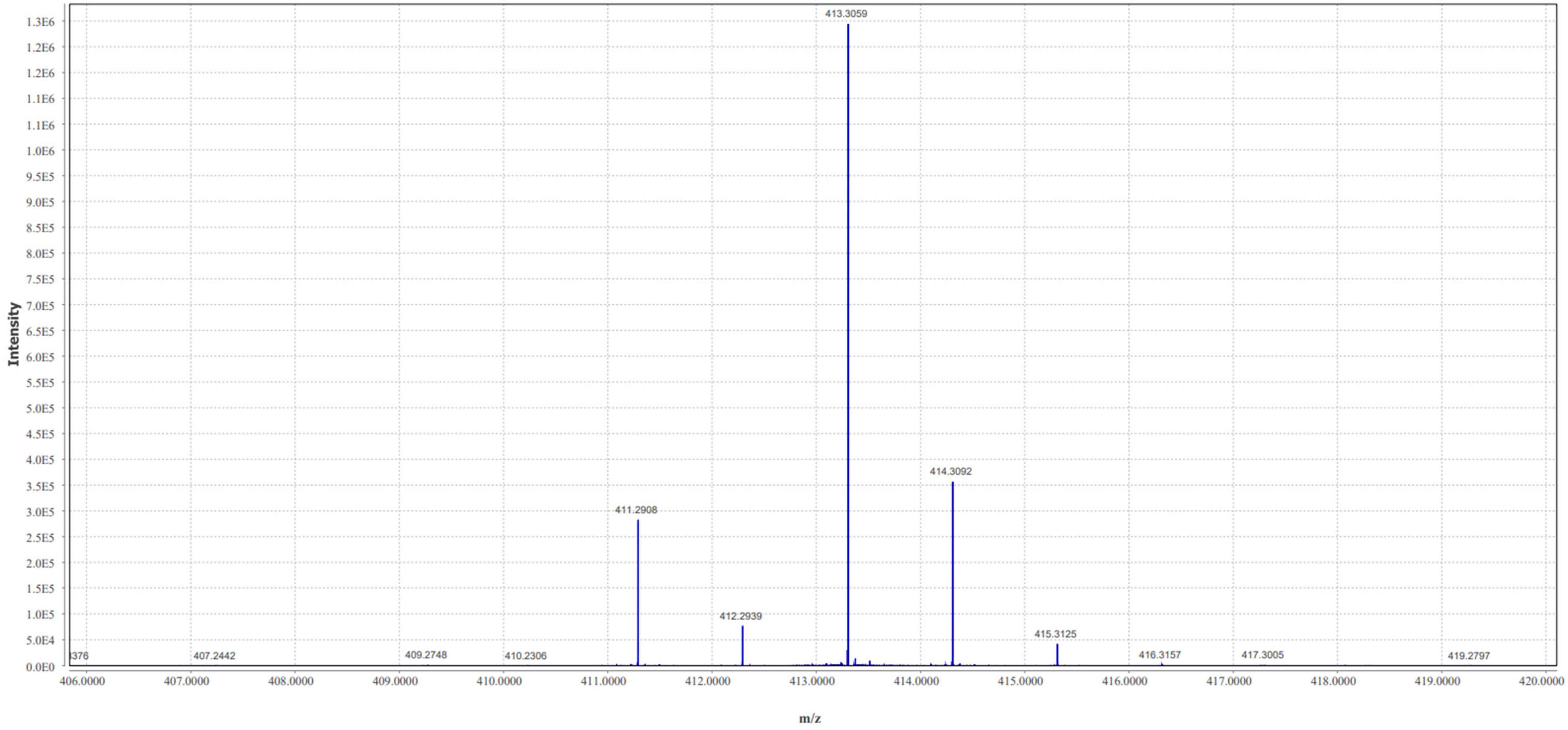

HRMS-ESI (*m/z*): [M-H]$^+$ calcd for $C_{27}H_{41}O_3$ 413,3056; found 413,3059

3-Oxo-cholest-4-en-26-oic acid (**414) (V**)

Figure S19. Mass spectrum [M-H]$^+$

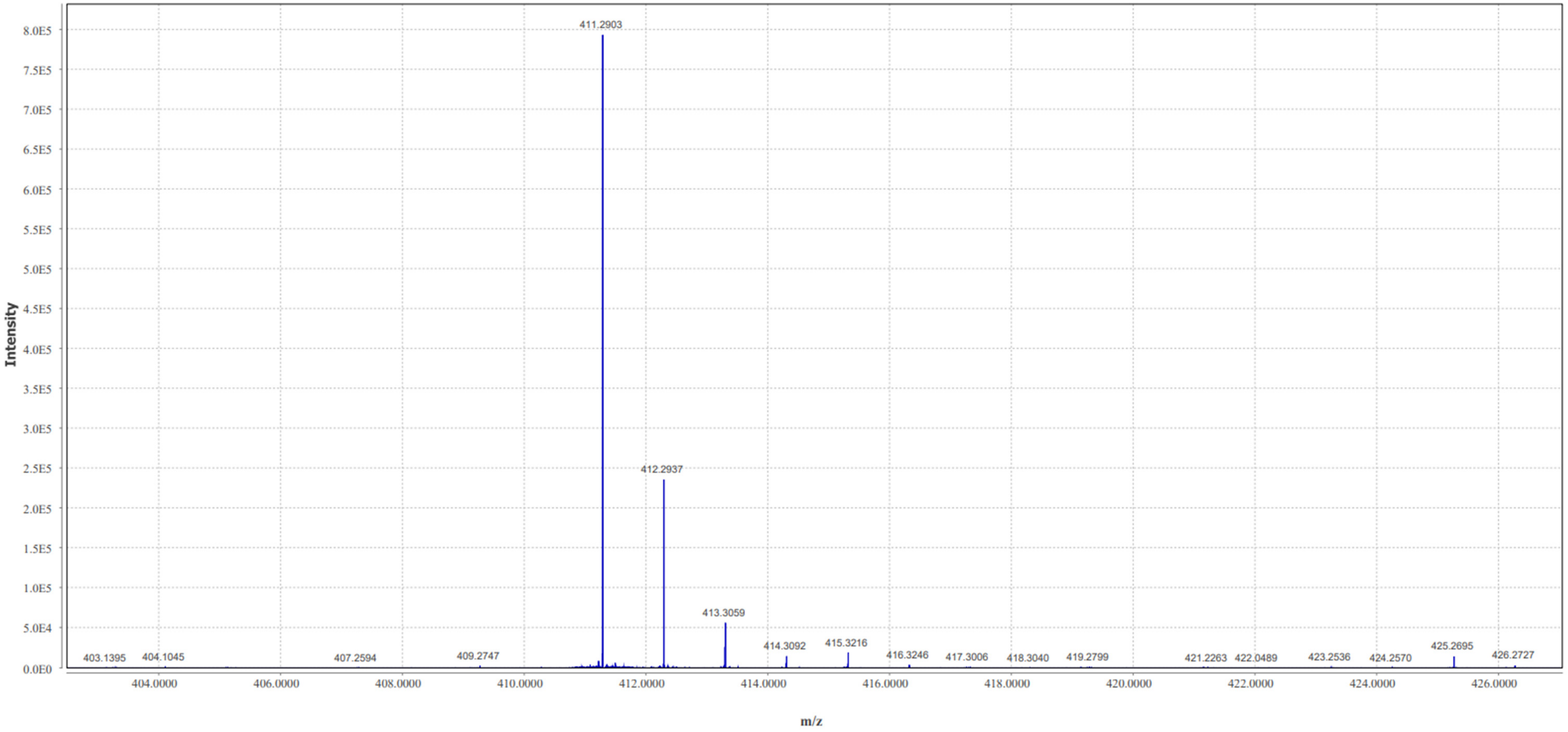

HRMS-ESI (*m/z*): [M-H]$^+$ calcd for $C_{27}H_{39}O_3$ 411,2899; found 411, 2903

3-Oxo-cholesta-1,4-dien-26-oic acid (**412**) (**VI**)

Figure S20. **Mass spectrum [M-H]$^+$**

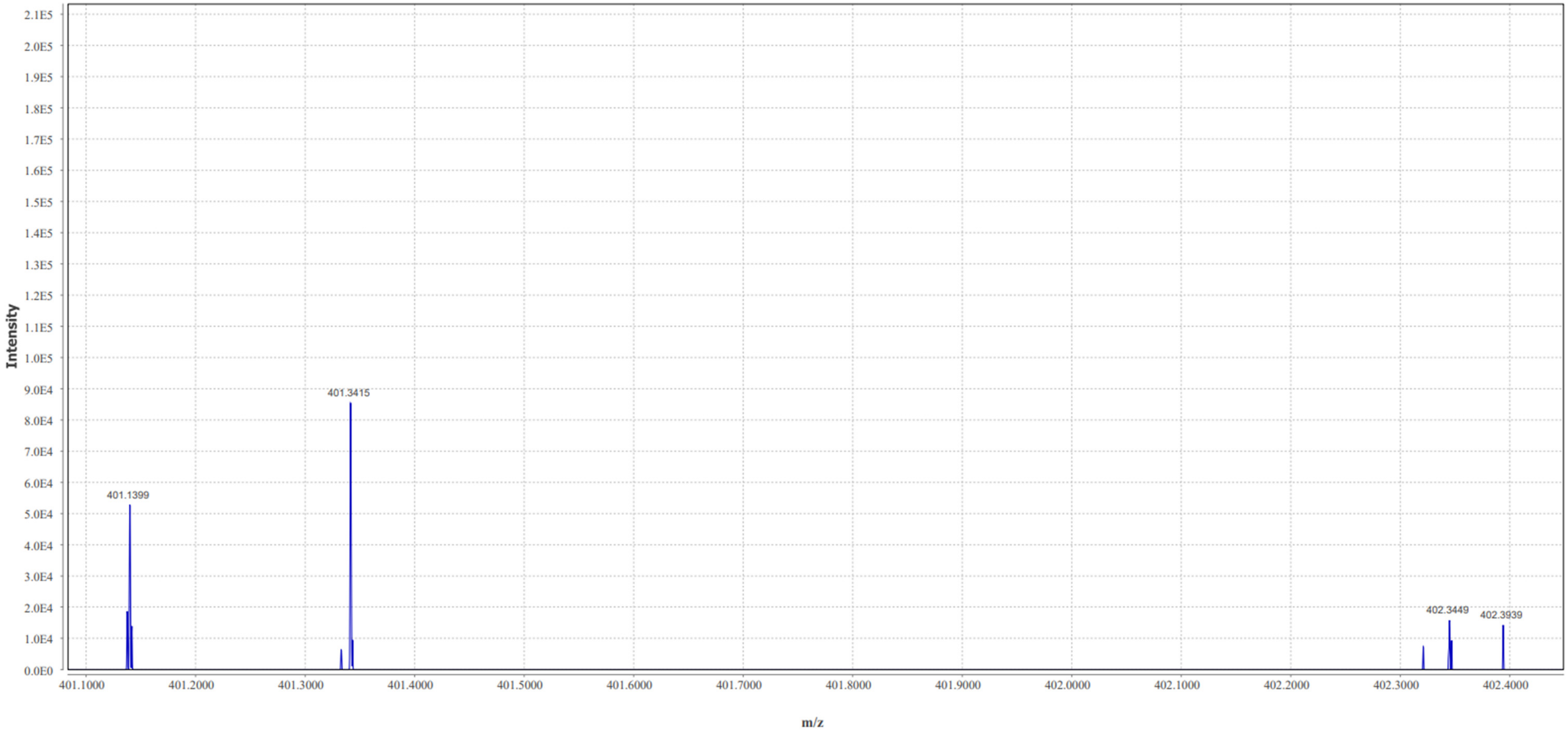

HRMS-ESI (m/z): [M-H]$^+$ calcd for $C_{27}H_{45}O_2$ 401,3420; found 401,3415

26-Hydroxycholesterol (**402**) (**VII**)

Figure S21. Mass spectrum $[M-H]^+$

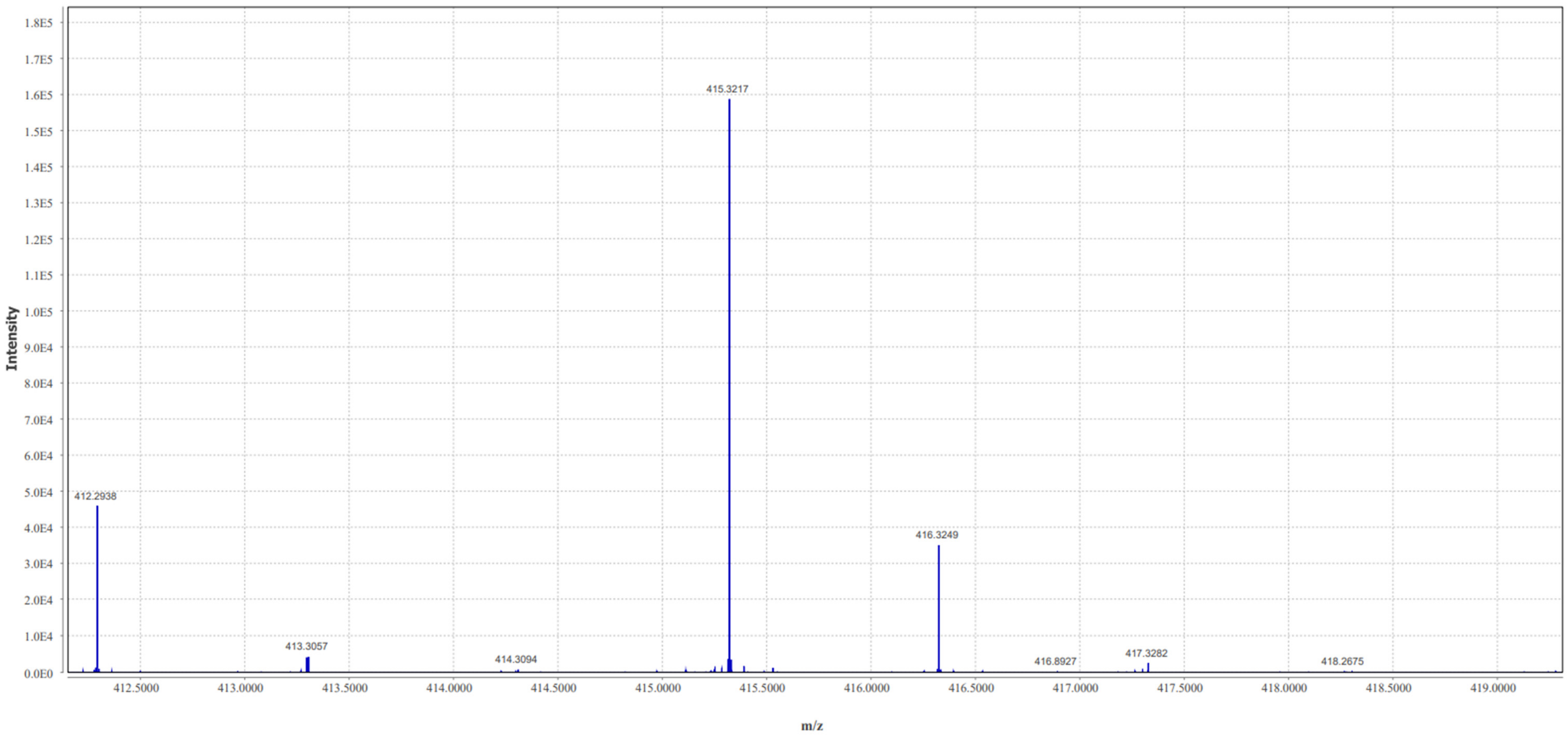

HRMS-ESI (*m/z*): $[M-H]^+$ calcd for $C_{27}H_{43}O_3$ 415,3212; found 415,3217

3β-Hydroxycholest-5-en-26-oic acid (**416**) (**VIII**)

Figure S22. **^{1}H-NMR spectrum**

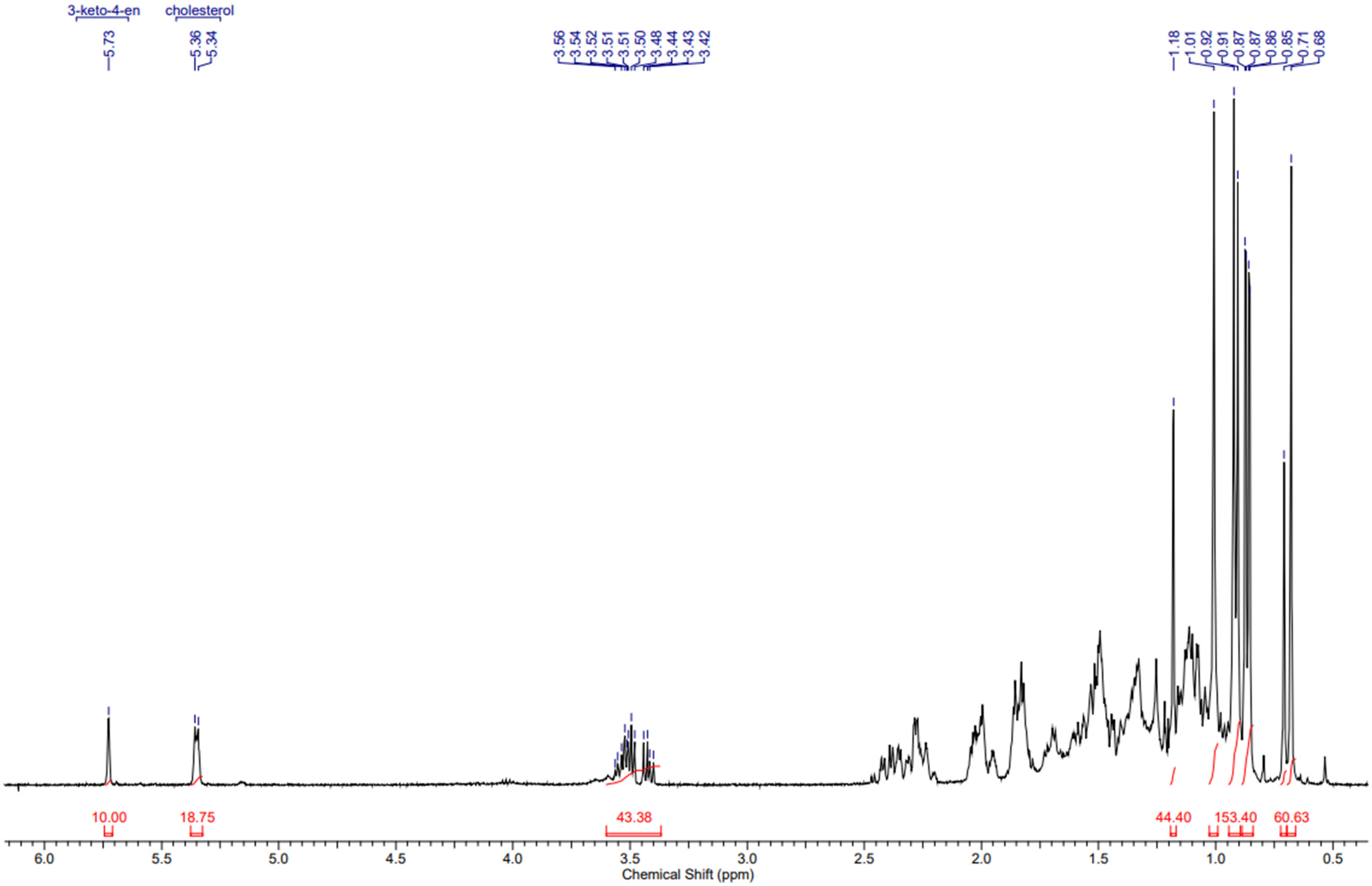

26-Hydroxycholest-4-en-3-one (**IV**)

Figure S23. ^{1}H-NMR spectrum

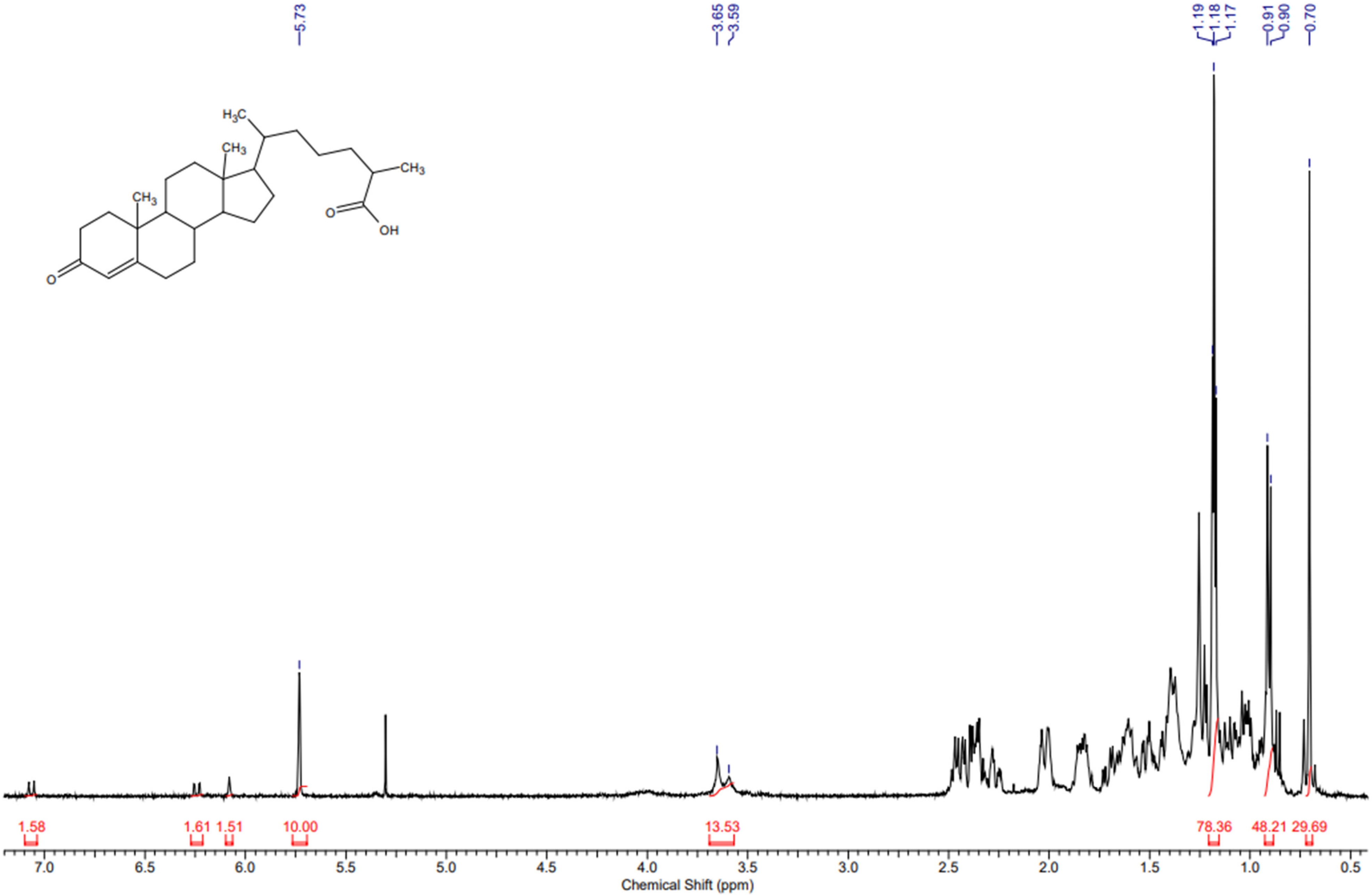

3-Oxo-cholest-4-en-26-oic acid (**V**)

Figure S24. ^{13}C-NMR spectrum

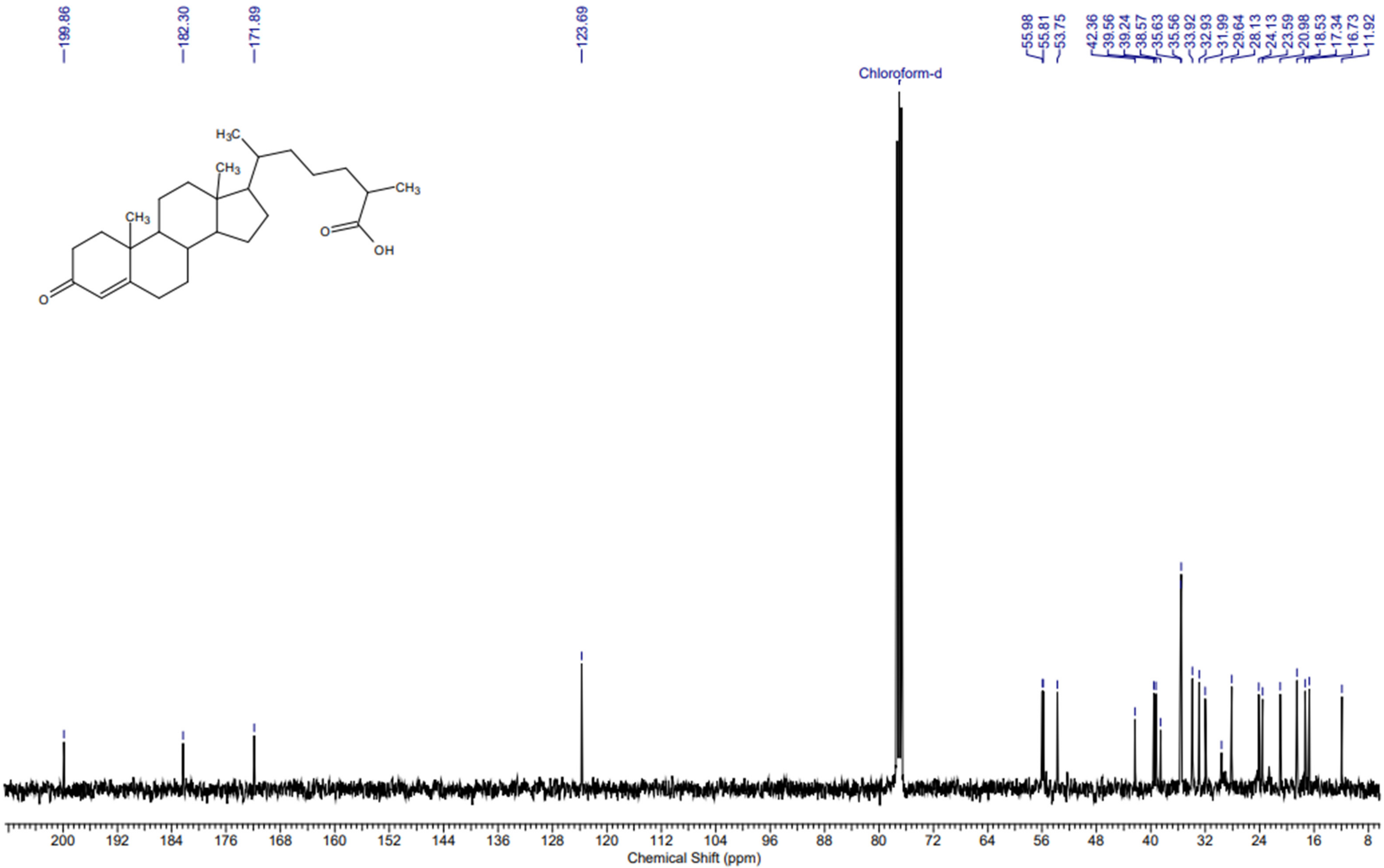

3-Oxo-cholest-4-en-26-oic acid (**V**)

Figure S25. ^{1}**H-NMR spectrum**

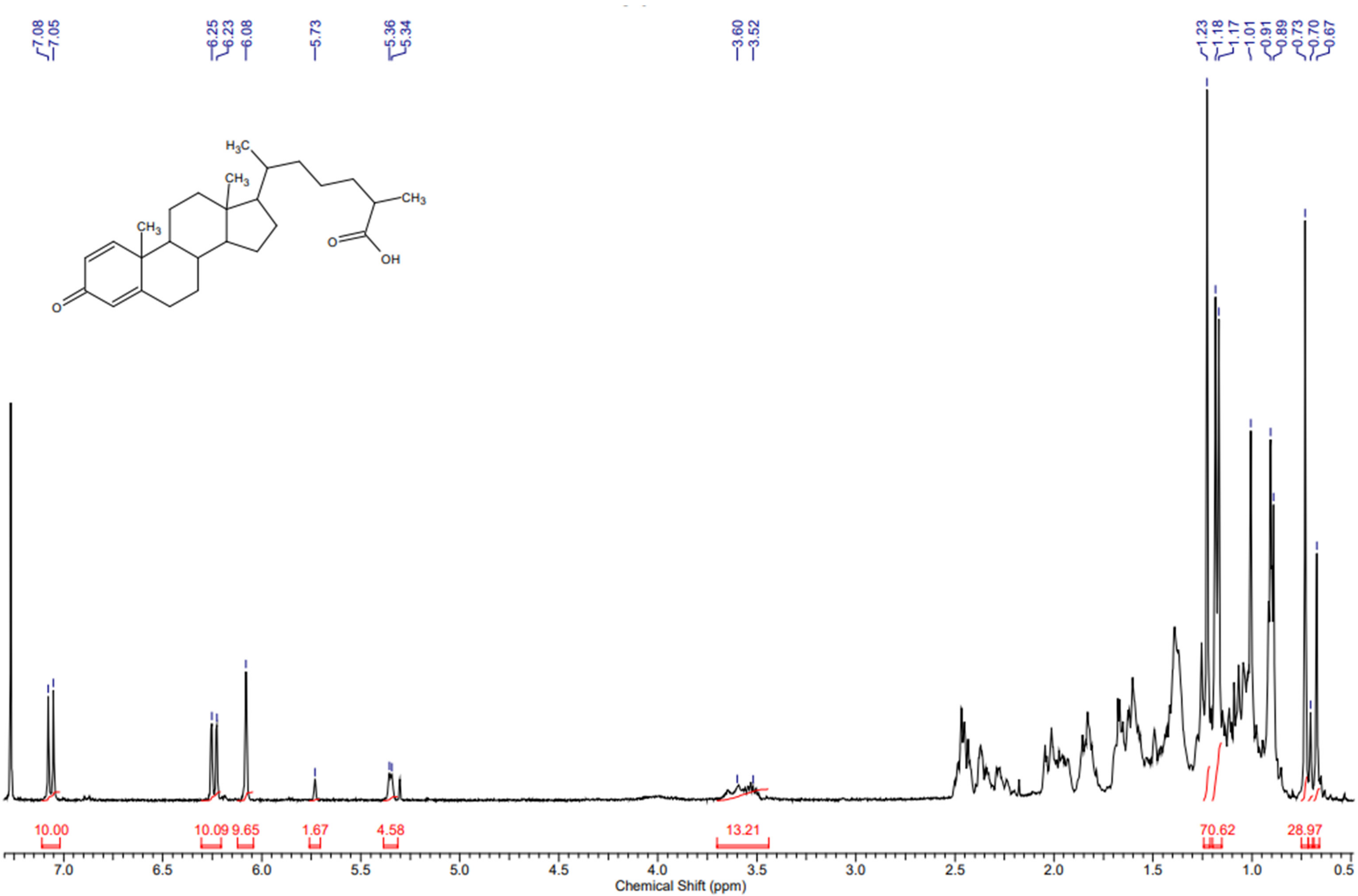

3-Oxo-cholesta-1,4-dien-26-oic acid (**VI**)

Figure S26. ^{13}C-NMR spectrum

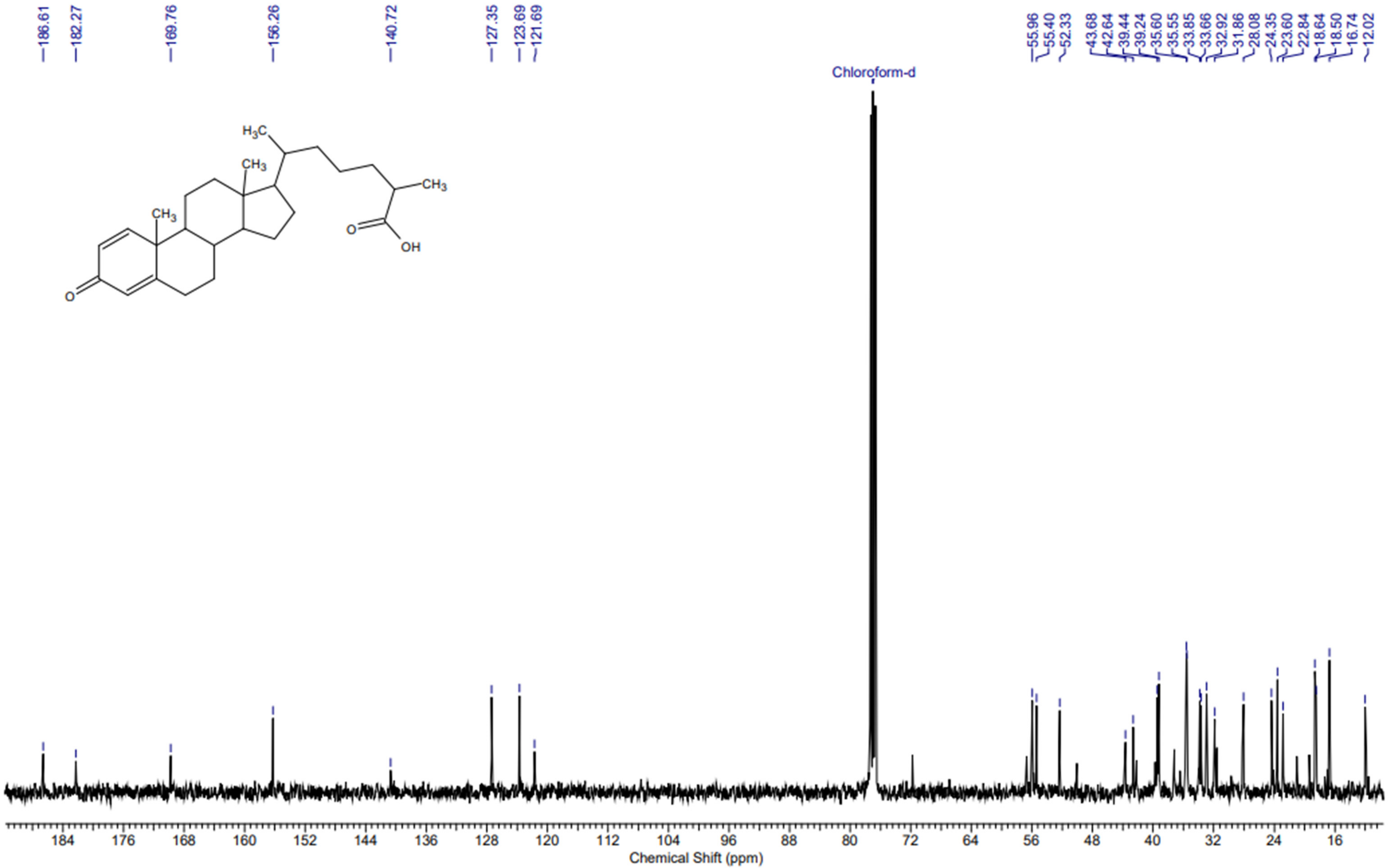

3-Oxo-cholesta-1,4-dien-26-oic acid (**VI**)

Figure S27. ^{1}H-NMR spectrum

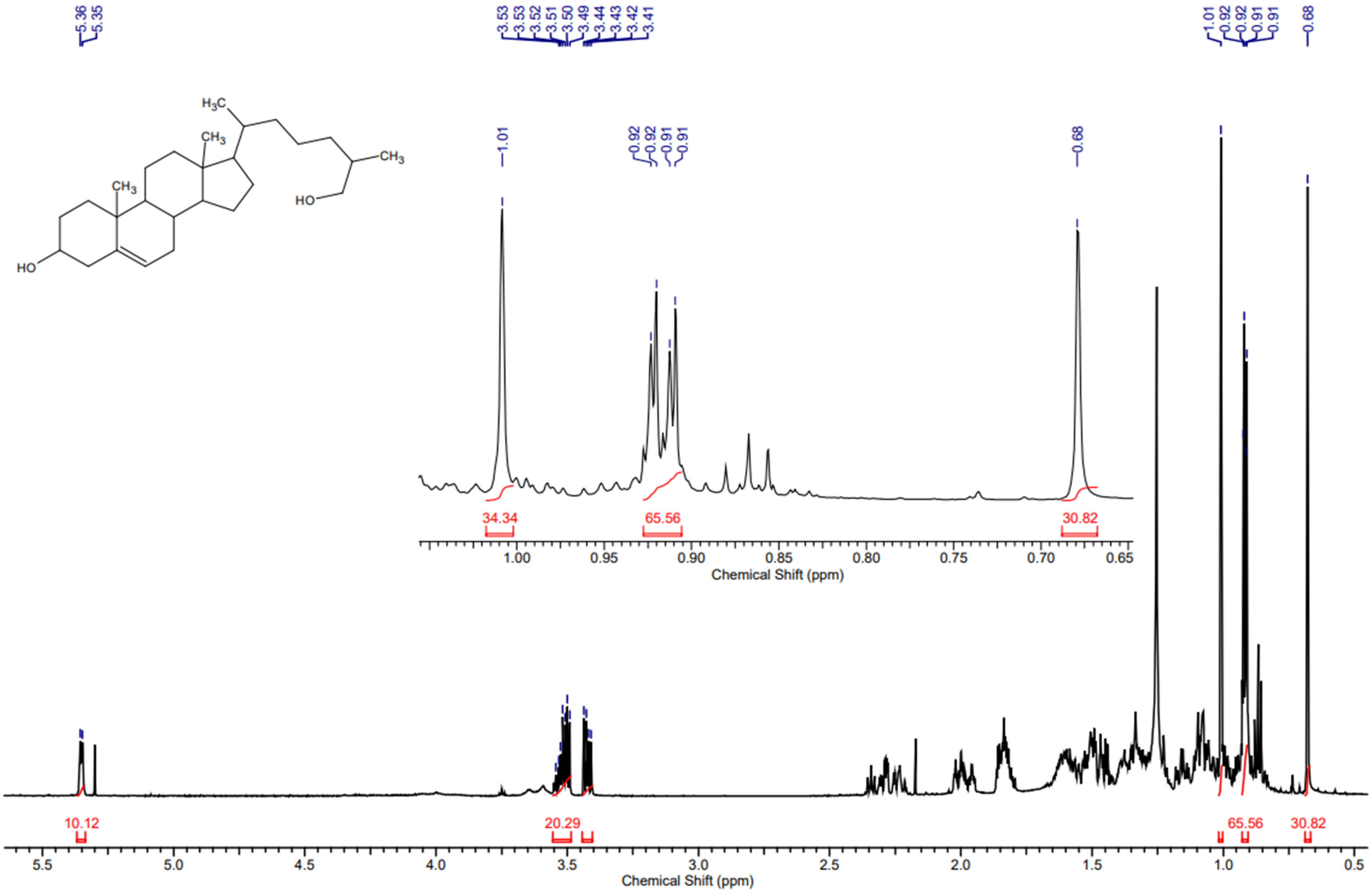

26-Hydroxycholesterol (**VII**)

Figure S28. ^{13}C-NMR spectrum

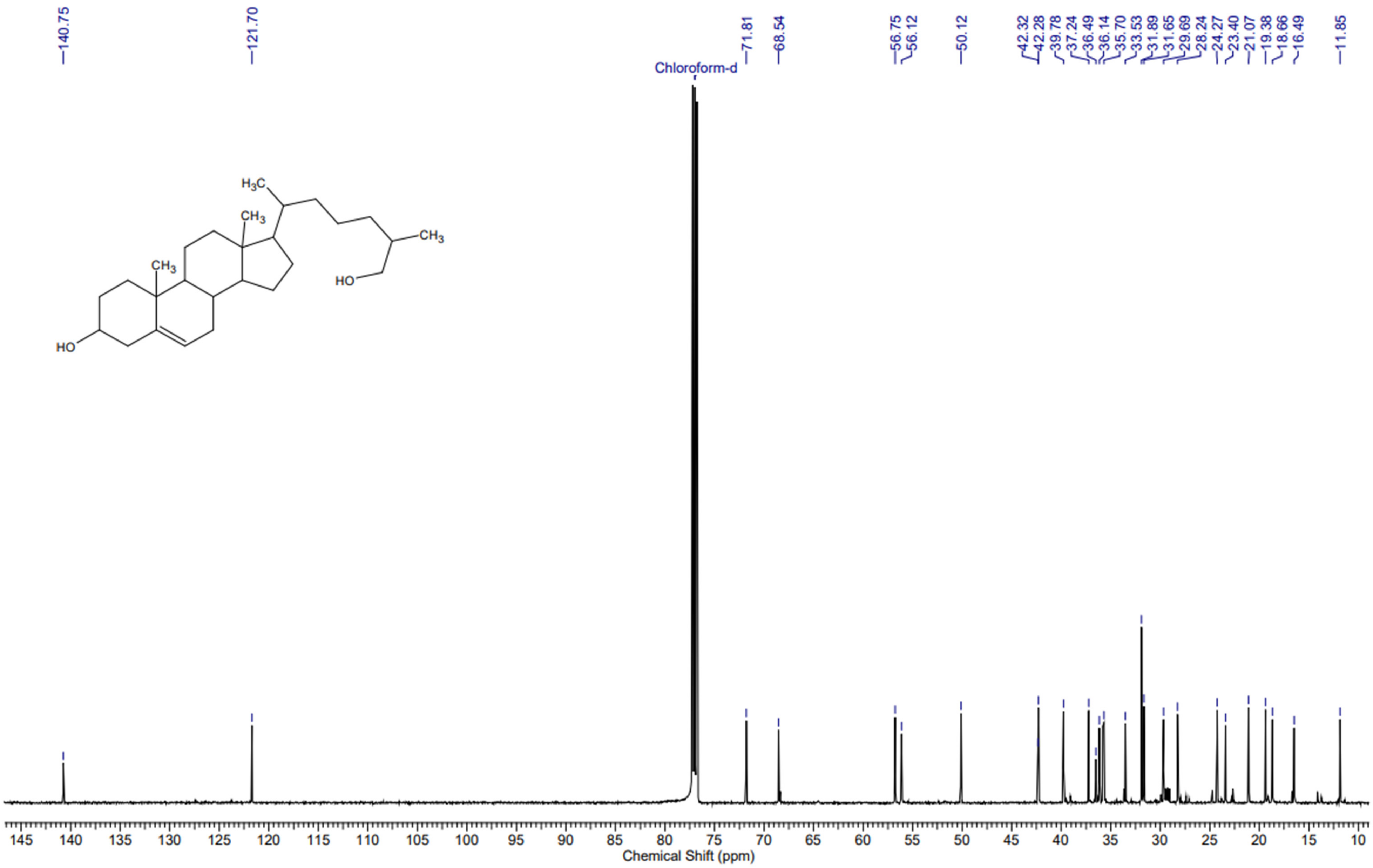

26-Hydroxycholesterol (**VII**)

Figure S29. ^{1}**H-NMR spectrum**

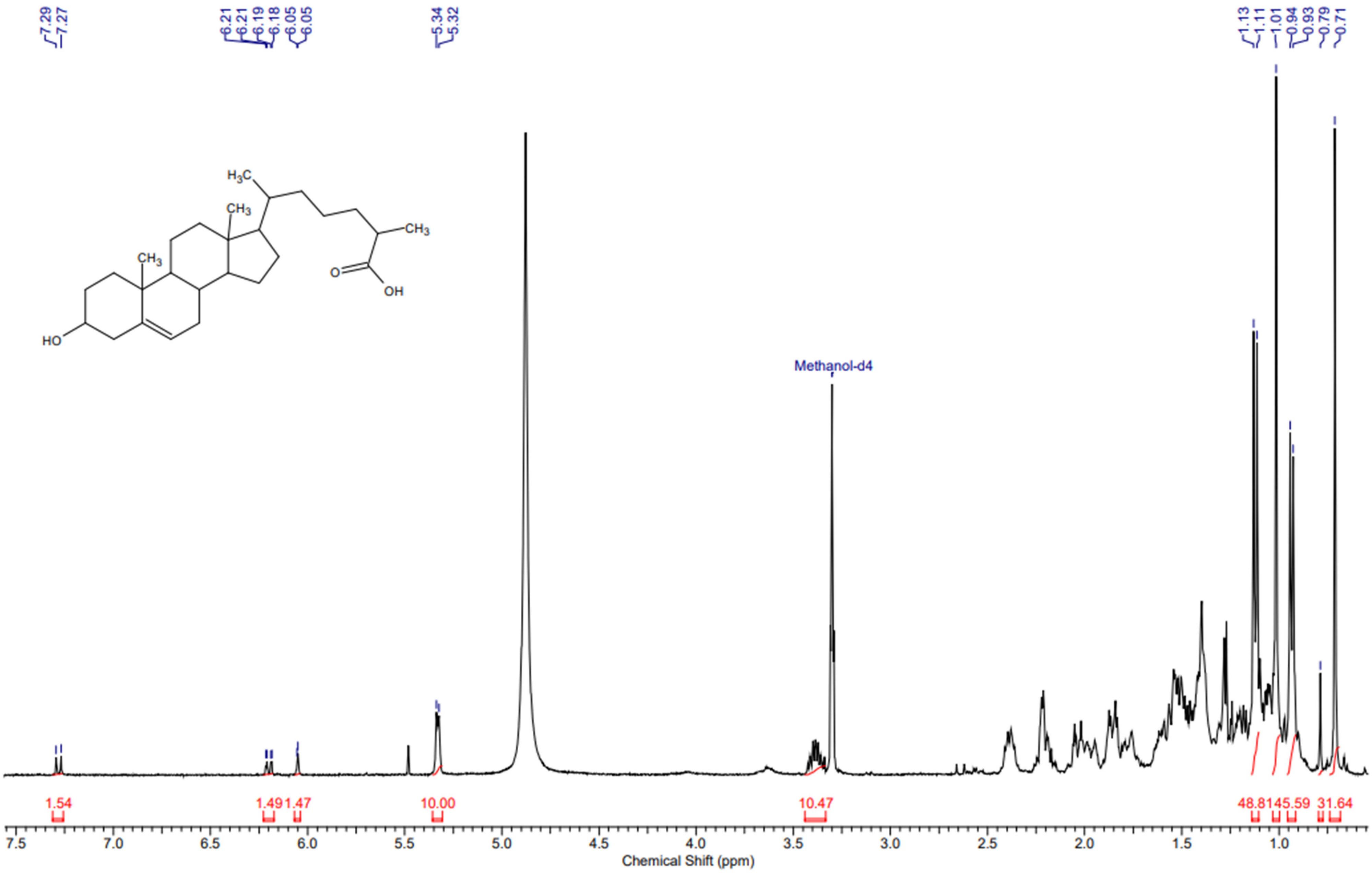

3β-Hydroxycholest-5-en-26-oic acid (**VIII**)

Figure S30. ^{13}**C-NMR spectrum**

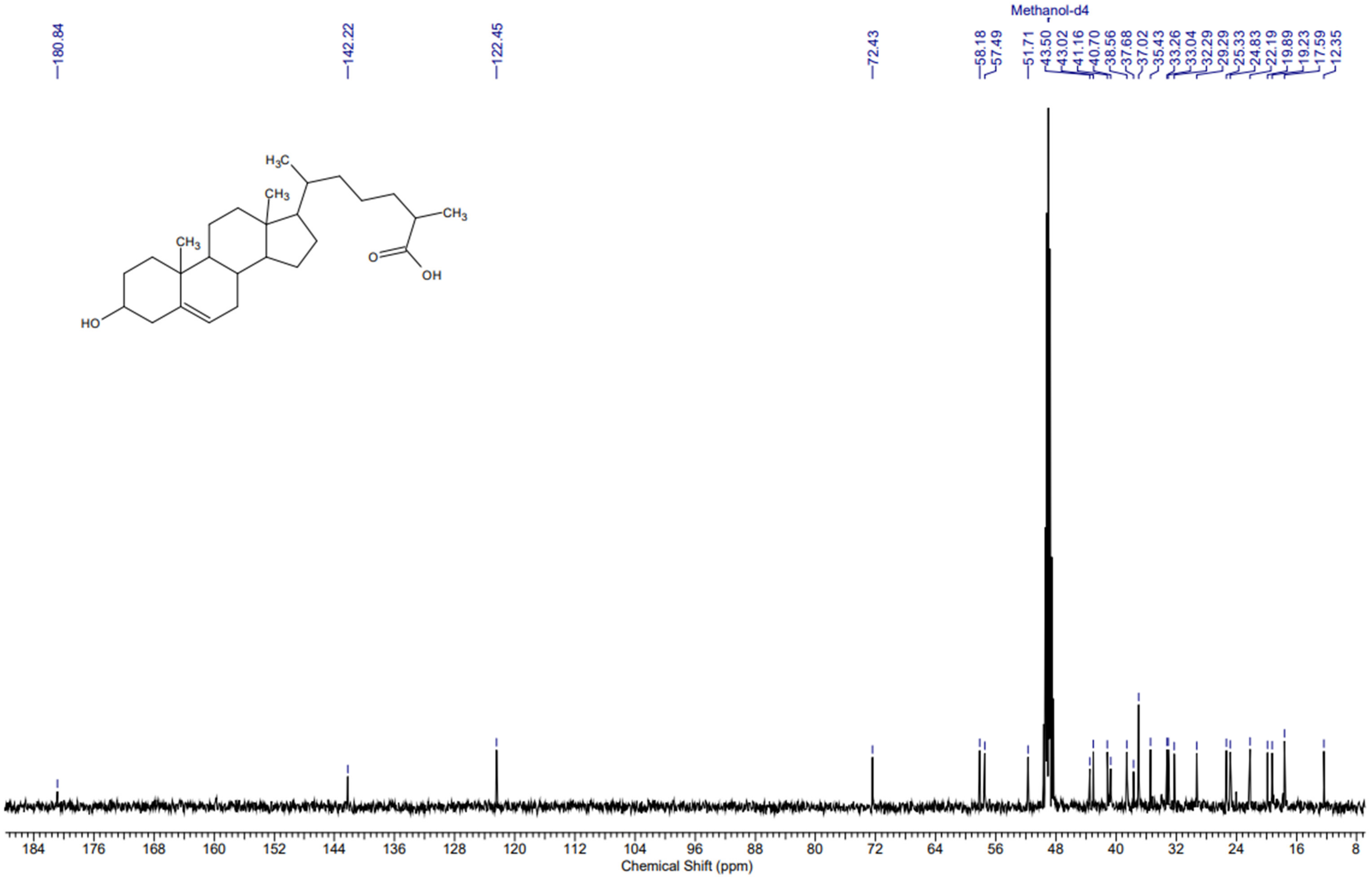

3β-Hydroxycholest-5-en-26-oic acid (**VIII**)

Figure S31. Bioconversion of lithocholic acid by *S.hirsuta* VKM Ac-666^T under the conditions used

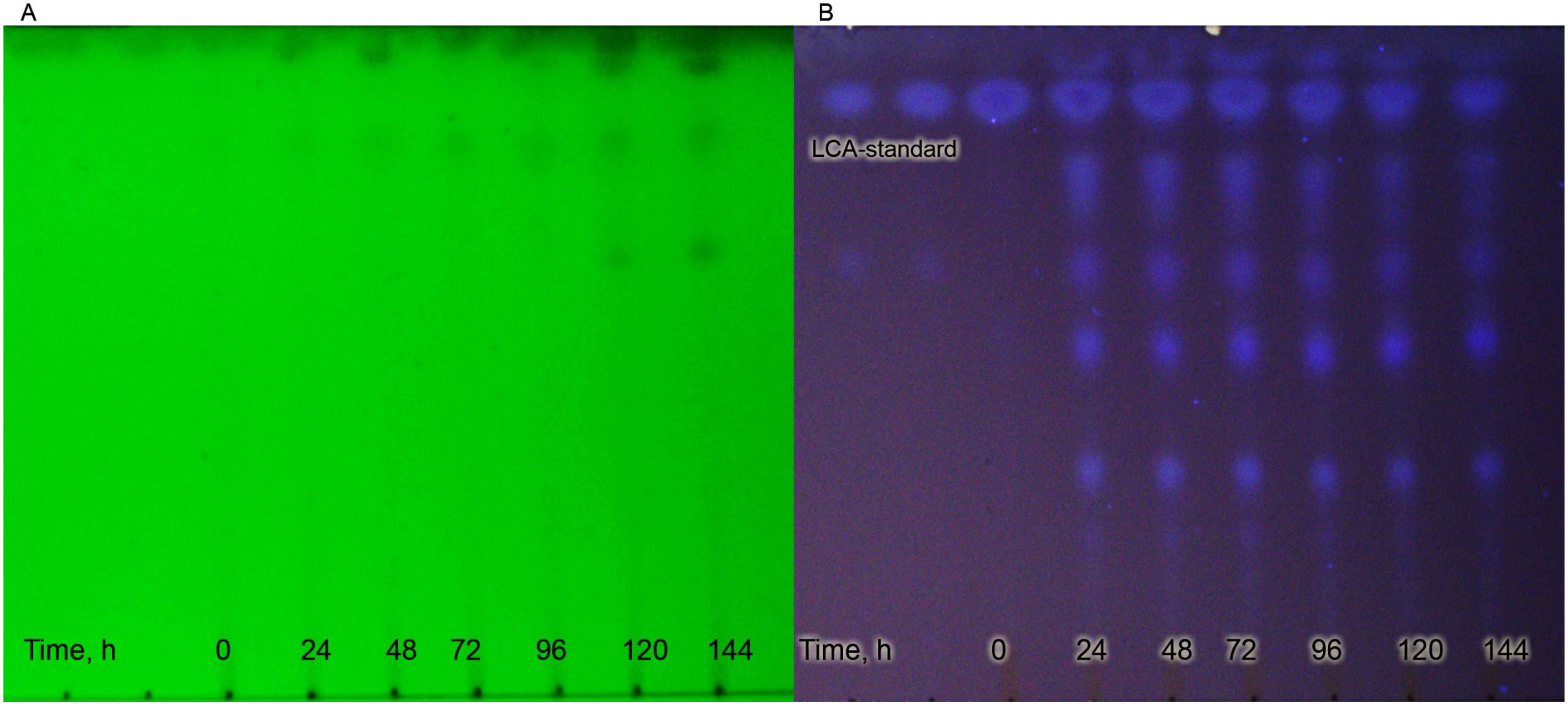

A. 3-Keto-4-ene steroids, system $CHCl_3$/acetone/CH_3COOH (50:50:0.5), visualization by UV light (254 nm)

B. Compounds with the unchanged A-ring structure, system $CHCl_3$/acetone/CH_3COOH (50:50:0.5); staining of TLC plates using $MnCl_2$ solution, visualization by UV light (365 nm), reference compound – lithocholic acid (LCA)
