# Supplementary material for: Steroid Metabolism in Thermophilic Actinobacterium Saccharopolyspora hirsuta VKM Ac-666T
Source: Microorganisms. 2021 Dec 10;9(12):2554. doi: 10.3390/microorganisms9122554 (PMC8708139; doi:10.3390/microorganisms9122554)
Supplement: Supplementary file 1 [file microorganisms-09-02554-s001.zip › Supplementary Table S1.pdf]

Supporting materials

article title: Steroid Metabolism in Thermophilic Actinobacteria *Saccharopolyspora hirsuta* VKM Ac-666^{T}

journal name: Microorganisms

author names: Tatyana Lobastova, Victoria Fokina, Sergey Tarlachkov, Andrey Shutov, Eugeny Bragin, Alexey Kazantsev, Marina Donova

***Correspondence:** 2vvfokina@gmail.com

Supplementary Table S1. List of thermophilic and thermotolerant bacteria to be screened for the key genes of the steroid catabolic 9,10-*seco*-pathway.

**No.**	**Strain**	**GenBank ID**
**1.**	*Acidimicrobium ferrooxidans* DSM 10331	CP001631.1
**2.**	*Acidithiobacillus thiooxidans* ATCC 19377	CP045571.1
**3.**	*Acidothermus cellulolyticus* 11B	CP000481.1
**4.**	*Alicyclobacillus acidocaldarius* subsp. *acidocaldarius* DSM 446	CP001727.1
**5.**	*Amycolatopsis granulosa* DSM 45669	JAANOV010000001.1
**6.**	*Amycolatopsis methanolica* 239	AQUL01000001.1-AQUL01000002.1
**7.**	*Amycolatopsis ruanii* 49.3e	PQHY01000001-PQHY01000170
**8.**	*Amycolatopsis thermalba* 50.9b	PQHX01000001-PQHX01000181
**9.**	*Bacillus circulans* strain FDAARGOS 783	CP053989.1
**10.**	*Bacillus coagulans* ATCC 7050	CP009709.1

**11.**	*Bacillus licheniformis* ATCC 14580	CP000002.3
**12.**	*Bacillus subtilis*_subsp. *subtilis* 168	AL009126.3
**13.**	*Bifidobacterium thermophilum* 1543B	PCGX01000001-<br>PCGX01000016
**14.**	*Brevibacillus laterosporus* DSM 25	CP017705.1
**15.**	*Chloroflexus* sp. Y-400-fl	CP001364.1
**16.**	*Desulfotomaculum nigrificans* DSM 574	AEVP02000001-<br>AEVP02000007
**17.**	*Geobacillus kaustophilus* HTA426	BA000043.1
**18.**	*Geobacillus thermocatenulatus* strain KCTC 3921	CP018058.1
**19.**	*Laceyella sacchari* DSM 43356	SMDD01000001-<br>SMDD01000021
**20.**	*Leptospira biflexa* 201601962	RQHA01000001-<br>RQHA01000012
**21.**	*Lysinibacillus sphaericus* DSM 28	CP019980.1
**22.**	*Methanothermobacter thermautotrophicus* Delta H	AE000666.1
**23.**	*Methylococcus capsulatus* Bath	AE017282.2
**24.**	*Microbispora rosea* subsp. *aerata* JCM 3076	BMPY01000001-<br>BMPY01000111
**25.**	*Microbispora siamensis* NBRC 104113	BOOF01000001.1-<br>BOOF01000148.1
**26.**	*Moorella thermoacetica strain* DSM 103284	CP017237.1
**27.**	*Parageobacillus thermoglucosidasius* strain NCIMB 11955	CP016622.1
**28.**	*Rubrobacter radiotolerans* strain RSPS-4	CP007514.1
**29.**	*Rubrobacter taiwanensis* Yellowstone	SKBU01000001-<br>SKBU01000057

**30.**	*Rubrobacter xylanophilus* DSM 9941	CP000386.1
**31.**	*Ruminiclostridium thermocellum* DSM 2360	CP016502.1
**32.**	*Saccharomonospora azurea* NA-128	CM001466.1
**33.**	*Saccharomonospora viridis* DSM 43017	CP001683.1
**34.**	*Saccharopolyspora rectivirgula* DSM 43113	JNVU01000001.1-JNVU01000050.1
**35.**	*Streptomyces calidiresistens* DSM 42108	VKHS01000001-VKHS01001643
**36.**	*Streptomyces thermoautotrophicus* UBT1	JYIJ01000001.1-JYIJ01000019.1
**37.**	*Streptomyces thermocarboxydus* BPSAC147	STGO01000001-STGO01000102
**38.**	*Streptomyces thermoviolaceus* subsp. *thermoviolaceus* JCM 4843	BMVZ01000001-BMVZ01000049
**39.**	*Streptomyces thermovulgaris* NRRL B-12375	MUME01000001-MUME01000595
**40.**	*Streptosporangium album* DSM 43023	JACHJU010000001-JACHJU010000014
**41.**	*Sulfolobus acidocaldarius* DG1	CP020364.1
**42.**	*Synechococcus lividus* PCC 6715	CP018092.1
**43.**	*Thermoactinomyces vulgaris* strain 2H	CP039710.1
**44.**	*Thermoanaerobacterium thermosaccharolyticum* DSM 571	CP002171.1
**45.**	*Thermobifida fusca* YX	CP000088.1
**46.**	*Thermobispora bispora* DSM 43833	CP001874.1
**47.**	*Thermocatellispora tengchongensis* DSM 45615	JACHGN010000001.1-JACHGN010000063.1

**48.**	*Thermodesulfobacterium thermophilum* DSM_1276	ATXI01000001-<br>ATXI01000046
**49.**	*Thermomonospora curvata* DSM 43183	CP001738.1
**50.**	*Thermoplasma acidophilum* DSM 1728	AL139299.1
**51.**	*Thermus aquaticus* Y51MC23	CP010822.1
**52.**	*Thermus thermophilus* HB8	AP008226.1
