# Supplementary material for: Steroid Metabolism in Thermophilic Actinobacterium Saccharopolyspora hirsuta VKM Ac-666T
Source: Microorganisms. 2021 Dec 10;9(12):2554. doi: 10.3390/microorganisms9122554 (PMC8708139; doi:10.3390/microorganisms9122554)
Supplement: Supplementary file 1 [file microorganisms-09-02554-s001.zip › Supplementary Table S2.pdf]

**Supporting materials**

**article title**: Steroid Metabolism in Thermophilic Actinobacteria *Saccharopolyspora hirsuta* VKM Ac-666$^T$

**journal name:** Microorganisms

**author names:** Tatyana Lobastova, Victoria Fokina, Sergey Tarlachkov, Andrey Shutov, Eugeny Bragin, Alexey Kazantsev, Marina Donova

***Correspondence:** 2vvfokina@gmail.com

**Supplementary Table S2.** List of enzymes of steroid catabolism for BLAST+ search.

| No. | Enzyme | Strain | Protein accession number |
|---|---|---|---|
| **1.** | ChoD | *Mycobacterium tuberculosis* H37Rv | CAB01014.1 |
| **2.** | Ltp4 | *Mycobacterium tuberculosis* UT205 | CCE38944.1 |
| **3.** | Ltp3 | *Mycobacterium tuberculosis* UT205 | CCE38945.1 |
| **4.** | Hsd4A | *Mycobacterium tuberculosis* H37Rv | NP_218019.1 |
| **5.** | FadE26 | *Mycobacterium tuberculosis* H37Rv | NP_218021.1 |
| **6.** | FadE27 | *Mycobacterium tuberculosis* H37Rv | NP_218022.1 |
| **7.** | FadD17 | *Mycobacterium tuberculosis* H37Rv | NP_218023.1 |
| **8.** | EchA19 | *Mycobacterium tuberculosis* H37Rv | NP_218033.1 |
| **9.** | KshA | *Mycobacterium tuberculosis* H37Rv | NP_218043.1 |
| **10.** | HsaF | *Mycobacterium tuberculosis* H37Rv | NP_218051.1 |
| **11.** | HsaE | *Mycobacterium tuberculosis* H37Rv | NP_218053.1 |
| **12.** | KstD | *Mycobacterium tuberculosis* H37Rv | NP_218054.1 |
| **13.** | FadE29 | *Mycobacterium tuberculosis* H37Rv | NP_218060.1 |
| **14.** | FadE28 | *Mycobacterium tuberculosis* H37Rv | NP_218061.1 |

| | | | |
|---|---|---|---|
| **15.** | Cyp125 | *Mycobacterium tuberculosis* H37Rv | NP_218062.1 |
| **16.** | FadA5 | *Mycobacterium tuberculosis* H37Rv | NP_218063.1 |
| **17.** | EchA20 | *Mycobacterium tuberculosis* H37Rv | NP_218067.1 |
| **18.** | FadE30 | *Mycobacterium tuberculosis* H37Rv | NP_218077.1 |
| **19.** | FadD3 | *Mycobacterium tuberculosis* H37Rv | NP_218078.1 |
| **20.** | HsaB | *Mycobacterium tuberculosis* H37Rv | NP_218084.1 |
| **21.** | HsaC | *Mycobacterium tuberculosis* H37Rv | NP_218085.1 |
| **22.** | HsaD | *Mycobacterium tuberculosis* H37Rv | NP_218086.1 |
| **23.** | HsaA | *Mycobacterium tuberculosis* H37Rv | NP_218087.1 |
| **24.** | KshB | *Mycobacterium tuberculosis* H37Rv | NP_218088.1 |
| **25.** | IpdB | *Mycobacterium tuberculosis* H37Rv | P9WPV9.1 |
| **26.** | ChsH1 | *Mycobacterium tuberculosis* | WP_003419290.1 |
| **27.** | ChsH2 | *Mycobacterium canettii* | WP_014001796.1 |
| **28.** | FadD19 | *Mycobacterium tuberculosis* H37Rv | YP_177983.1 |
| **29.** | Hsd4B | *Mycobacterium tuberculosis* H37Rv | YP_177986.1 |
