# Supplementary material for: Steroid Metabolism in Thermophilic Actinobacterium Saccharopolyspora hirsuta VKM Ac-666T
Source: Microorganisms. 2021 Dec 10;9(12):2554. doi: 10.3390/microorganisms9122554 (PMC8708139; doi:10.3390/microorganisms9122554)
Supplement: Supplementary file 1 [file microorganisms-09-02554-s001.zip › Supplementary Table S3.pdf]

Supporting materials

article title: Steroid Metabolism in Thermophilic Actinobacteria *Saccharopolyspora hirsuta* VKM Ac-666^{T}

journal name: Microorganisms

author names: Tatyana Lobastova, Victoria Fokina, Sergey Tarlachkov, Andrey Shutov, Eugeny Bragin, Alexey Kazantsev, Marina Donova

***Correspondence:** 2vvfokina@gmail.com

Supplementary Table S3. Candidate genes for steroids or bile acids degradation in *S. hirsuta* VKM Ac-666^{T}.

No.	Scaffold acc.	Locus tag	Protein acc.	Name/Function
Mce4 transport system				
1.	VWPH01000020.1	*F1721_32550*	KAA5825886.1	*mce4F*
2.	VWPH01000020.1	*F1721_32555*	KAA5825887.1	*mce4E*
3.	VWPH01000020.1	*F1721_32560*	KAA5825888.1	*mce4D*
4.	VWPH01000020.1	*F1721_32565*	KAA5825937.1	*mce4C*
5.	VWPH01000020.1	*F1721_32570*	KAA5825889.1	*mce4B*
6.	VWPH01000020.1	*F1721_32575*	KAA5825890.1	*mce4A*
7.	VWPH01000020.1	*F1721_32580*	KAA5825891.1	*yrbEb*
8.	VWPH01000020.1	*F1721_32585*	KAA5825892.1	*yrbEa*
Sterol side chain degradation				
9.	VWPH01000020.1	*F1721_32590*	KAA5825893.1	*fabG3*
10.	VWPH01000020.1	*F1721_32595*	KAA5825894.1	aldehyde dehydrogenase
11.	VWPH01000020.1	*F1721_32600*	KAA5825895.1	*hsd4A*
12.	VWPH01000020.1	*F1721_32605*	KAA5825896.1	*fadE26*

13.	VWPH01000020.1	*F1721_32610*	KAA5825897.1	*fadE27*
14.	VWPH01000020.1	*F1721_32615*	KAA5825898.1	*fadD17*
15.	VWPH01000020.1	*F1721_32620*	KAA5825899.1	alpha/beta hydrolase
16.	VWPH01000020.1	*F1721_32625*	KAA5825900.1	MerR family transcriptional regulator
17.	VWPH01000020.1	*F1721_32630*	KAA5825901.1	nitronate monooxygenase
18.	VWPH01000020.1	*F1721_32635*	KAA5825902.1	*fadD19*
19.	VWPH01000020.1	*F1721_32640*	KAA5825903.1	*echA19*
20.	VWPH01000020.1	*F1721_32645*	KAA5825904.1	acyl-CoA synthetase
21.	VWPH01000020.1	*F1721_32650*	KAA5825905.1	LLM class F420-dependent oxidoreductase
22.	VWPH01000020.1	*F1721_32655*	KAA5825906.1	DNA-binding protein
23.	VWPH01000020.1	*F1721_32660*	KAA5825907.1	*ltp4*
24.	VWPH01000020.1	*F1721_32665*	KAA5825908.1	*ltp3*
25.	VWPH01000020.1	*F1721_32670*	KAA5825909.1	F420-dependent LLM class oxidoreductase
26.	VWPH01000020.1	*F1721_32675*	KAA5825910.1	*ksdI*
27.	VWPH01000020.1	*F1721_32680*	KAA5825911.1	*cyp125*
28.	VWPH01000020.1	*F1721_32685*	KAA5825912.1	*fadA5*
29.	VWPH01000020.1	*F1721_32690*	KAA5825913.1	DUF4180 domain-containing protein
Ring A/B oxidation				
30.	VWPH01000020.1	*F1721_32695*	KAA5825938.1	helix-turn-helix domain-

				containing protein
31.	VWPH01000020.1	*F1721_32700*	KAA5825914.1	*hsaB*
32.	VWPH01000020.1	*F1721_32705*	KAA5825915.1	*hsaC*
33.	VWPH01000020.1	*F1721_32710*	KAA5825916.1	*hsaD*
34.	VWPH01000020.1	*F1721_32715*	KAA5825917.1	*hsaA*
35.	VWPH01000020.1	*F1721_32720*	KAA5825918.1	*hsaF*
36.	VWPH01000020.1	*F1721_32725*	KAA5825919.1	*hsaG*
37.	VWPH01000020.1	*F1721_32730*	KAA5825920.1	*hsaE*
38.	VWPH01000020.1	*F1721_32735*	KAA5825921.1	*kstD4*
39.	VWPH01000020.1	*F1721_32740*	KAA5825922.1	*kstD3*
40.	VWPH01000020.1	*F1721_32745*	KAA5825923.1	*kshA*
41.	VWPH01000020.1	*F1721_32750*	KAA5825924.1	hypothetical protein
42.	VWPH01000020.1	*F1721_32755*	KAA5825925.1	*kshB*
“*igr*-locus”				
43.	VWPH01000020.1	*F1721_32760*	KAA5825939.1	FAD-binding oxidoreductase
44.	VWPH01000020.1	*F1721_32765*	KAA5825926.1	WYL domain-containing protein
45.	VWPH01000020.1	*F1721_32770*	KAA5825927.1	*ltp2*
46.	VWPH01000020.1	*F1721_32775*	KAA5825928.1	*chsH1*
47.	VWPH01000020.1	*F1721_32780*	KAA5825929.1	*chsH2*
48.	VWPH01000020.1	*F1721_32785*	KAA5825930.1	*fadE29*
49.	VWPH01000024.1	*F1721_33645*	KAA5825036.1	*fadE28*
50.	VWPH01000024.1	*F1721_33650*	KAA5825037.1	class I SAM-dependent methyltransferase

51.	VWPH01000024.1	*F1721_33655*	KAA5825022.1	prenyltransferase
52.	VWPH01000024.1	*F1721_33660*	KAA5825023.1	methyltransferase domain-containing protein
53.	VWPH01000024.1	*F1721_33665*	KAA5825024.1	glycosyltransferase family 4 protein
Transcriptional regulator				
54.	VWPH01000024.1	*F1721_33670*	KAA5825025.1	*kstR*
Ring C/D degradation				
55.	VWPH01000024.1	*F1721_33675*	KAA5825026.1	MBL fold metallo-hydrolase
56.	VWPH01000024.1	*F1721_33680*	KAA5825027.1	SDR family oxidoreductase
57.	VWPH01000024.1	*F1721_33685*	KAA5825028.1	*echA20*
58.	VWPH01000024.1	*F1721_33690*	KAA5825029.1	*ipdA*
59.	VWPH01000024.1	*F1721_33695*	KAA5825030.1	*ipdB*
60.	VWPH01000024.1	*F1721_33700*	KAA5825038.1	*ipdC*
61.	VWPH01000024.1	*F1721_33705*	KAA5825039.1	*kstR2*
62.	VWPH01000024.1	*F1721_33710*	KAA5825031.1	*ipdF*
63.	VWPH01000024.1	*F1721_33715*	KAA5825032.1	*fadE30*
64.	VWPH01000024.1	*F1721_33720*	KAA5825033.1	*echA13*, enoyl-CoA hydratase
65.	VWPH01000024.1	*F1721_33725*	KAA5825040.1	*fadE31*
66.	VWPH01000024.1	*F1721_33730*	KAA5825034.1	*fadE32*
67.	VWPH01000024.1	*F1721_33735*	KAA5825035.1	*fadE33*

Cholate degradation pathway				
68.	VWPH01000001.1	*F1721_33645*	KAA5838019.1	*hsaE3*
69.	VWPH01000001.1	*F1721_00680*	KAA5838020.1	*hsaG3*
70.	VWPH01000001.1	*F1721_00685*	KAA5838021.1	*hsaF3*
71.	VWPH01000001.1	*F1721_00690*	KAA5838022.1	*casA*
72.	VWPH01000001.1	*F1721_00695*	KAA5838023.1	*hsaD3*
73.	VWPH01000001.1	*F1721_00700*	KAA5838439.1	*kstR3*
74.	VWPH01000001.1	*F1721_00705*	KAA5838024.1	SDR family NAD(P)-dependent oxidoreductase
75.	VWPH01000001.1	*F1721_00710*	KAA5838025.1	*kstD2*
76.	VWPH01000001.1	*F1721_00715*	KAA5838026.1	*kstD1*
77.	VWPH01000001.1	*F1721_00720*	KAA5838027.1	SDR family oxidoreductase
78.	VWPH01000001.1	*F1721_00725*	KAA5838028.1	*kshA*
79.	VWPH01000001.1	*F1721_00730*	KAA5838029.1	hypothetical protein
80.	VWPH01000001.1	*F1721_00735*	KAA5838030.1	*kshB*
81.	VWPH01000001.1	*F1721_00740*	KAA5838031.1	*ksdI*
82.	VWPH01000001.1	*F1721_00745*	KAA5838032.1	*hsaB3*
83.	VWPH01000001.1	*F1721_00750*	KAA5838440.1	SDR family oxidoreductase
84.	VWPH01000001.1	*F1721_00755*	KAA5838033.1	*hsaA3*
85.	VWPH01000001.1	*F1721_00760*	KAA5838034.1	*hsaC3*
HIP-CoA synthetase				
86.	VWPH01000019.1	*F1721_32060*	KAA5826116.1	*fadD3*

Thiolase				
87.	VWPH01000031.1	*F1721_34155*	KAA5824600.1	*fadA6*
Cholesterol oxidases				
88.	VWPH01000006.1	*F1721_14655*	KAA5833518.1	*choD*
89.	VWPH01000004.1	*F1721_09795*	KAA5835081.1	*choE*
Transcriptional regulator				
90.	VWPH01000015.1	*F1721_28735*	KAA5828487.1	*kstR3*
Cholate degradation pathway				
91.	VWPH01000015.1	*F1721_28740*	KAA5828429.1	class I SAM-dependent methyltransferase
92.	VWPH01000015.1	*F1721_28745*	KAA5828430.1	*casA*
93.	VWPH01000015.1	*F1721_28750*	KAA5828431.1	*casC/chsE3*
94.	VWPH01000015.1	*F1721_28755*	KAA5828432.1	*casE*
95.	VWPH01000015.1	*F1721_28760*	KAA5828433.1	*3α-hsd*
96.	VWPH01000015.1	*F1721_28765*	KAA5828434.1	*casH*
97.	VWPH01000015.1	*F1721_28770*	KAA5828435.1	*casI*
98.	VWPH01000001.1	*F1721_02365*	KAA5838304.1	*casE*
99.	VWPH01000001.1	*F1721_02405*	KAA5838310.1	*casG*
