# Supplementary material for: Steroid Metabolism in Thermophilic Actinobacterium Saccharopolyspora hirsuta VKM Ac-666T
Source: Microorganisms. 2021 Dec 10;9(12):2554. doi: 10.3390/microorganisms9122554 (PMC8708139; doi:10.3390/microorganisms9122554)
Supplement: Supplementary file 1 [file microorganisms-09-02554-s001.zip › Supplementary Table S4.pdf]

Supporting materials

article title: Steroid Metabolism in Thermophilic Actinobacteria *Saccharopolyspora hirsuta* VKM Ac-666^{T}

journal name: Microorganisms

author names: Tatyana Lobastova, Victoria Fokina, Sergey Tarlachkov, Andrey Shutov, Eugeny Bragin, Alexey Kazantsev, Marina Donova

***Correspondence:** 2vvfokina@gmail.com

Supplementary Table S4. List of homologous genes between *S. hirsuta* VKM Ac-666^{T}, *R. jostii* RHA1. *M. tuberculosis* H37Rv.

No.	*S. hirsuta* VKM Ac-666		*R. jostii* RHA1		*M. tuberculosis* H37Rv	
	Gene	Protein acc.	Protein acc.	Identity, % a.a.	Protein acc.	Identity, % a.a.
1.	*mce4F*	KAA5825886.1	WP_011597044.1	**34.1**	CCP46316.1	**29.9**
2.	*mce4E*	KAA5825887.1	WP_011597043.1	**37.1**	CCP46317.1	**33.2**
3.	*mce4D*	KAA5825888.1	WP_011597042.1	**34.1**	CCP46318.1	**33.3**
4.	*mce4C*	KAA5825937.1	WP_011597041.1	**34.0**	CCP46319.1	**32.2**
5.	*mce4B*	KAA5825889.1	WP_011597040.1	**45.9**	CCP46320.1	**31.4**
6.	*mce4A*	KAA5825890.1	WP_009477765.1	**29.3**	CCP46321.1	**19.8**
7.	*yrbEb*	KAA5825891.1	WP_011597039.1	**48.4**	CCP46322.1	**48.8**

8.	*yrbEa*	KAA5825892.1	WP_009477764.1	**47.9**	CCP46323.1	**47.9**
9.	*fabG3*	KAA5825893.1	WP_011597603.1	**50.0**	CCP44774.1	**50.0**
10.	*hsd4A*	KAA5825895.1	WP_011597038.1	**58.8**	CCP46324.1	**59.9**
11.	*fadE26*	KAA5825896.1	WP_009477760.1	**75.0**	CCP46326.1	**71.7**
12.	*fadE27*	KAA5825897.1	WP_011597036.1	**63.2**	CCP46327.1	**52.2**
13.	*fadD17*	KAA5825898.1	WP_011597035.1	**60.7**	CCP46328.1	**53.9**
14.	*fadD19*	KAA5825902.1	WP_009477756.1	**60.5**	CCP46337.1	**57.9**
15.	*echA19*	KAA5825903.1	WP_011597033.1	**77.2**	CCP46338.1	**71.9**
16.	*ltp4*	KAA5825907.1	WP_009477751.1	**69.3**	CCP46344.1	**67.3**
17.	*ltp3*	KAA5825908.1	WP_009477750.1	**80.8**	CCP46345.1	**78.8**
18.	*ksdI*	KAA5825910.1	WP_011597028.1	**50.3**	-	**-**
19.	*cyp125*	KAA5825911.1	WP_009477746.1	**73.3**	CCP46367.1	**66.2**
20.	*fadA5*	KAA5825912.1	WP_011597025.1	**74.6**	CCP46368.1	**71.7**
21.	*hsaB*	KAA5825914.1	WP_005244804.1	**56.2**	CCP46390.1	**51.9**
22.	*hsaC*	KAA5825915.1	WP_011596919.1	**80.6**	CCP46391.1	**81.6**
23.	*hsaD*	KAA5825916.1	WP_011596918.1	**73.7**	CCP46392.1	**65.3**

24.	*hsaA*	KAA5825917.1	WP_011596917.1	**68.7**	CCP46393.1	**64.6**
25.	*hsaF*	KAA5825918.1	WP_009477596.1	**82.1**	CCP46356.1	**79.2**
26.	*hsaG*	KAA5825919.1	WP_009477595.1	**71.4**	CCP46357.1	**68.5**
27.	*hsaE*	KAA5825920.1	WP_009477594.1	**65.3**	CCP46358.1	**64.1**
28.	*kstD4*	KAA5825921.1	WP_011597824.1	**31.1**	CCP44583.1	**33.1**
29.	*kstD3*	KAA5825922.1	WP_011596914.1	**65.8**	CCP46359.1	**62.2**
30.	*kshA*	KAA5825923.1	WP_016884082.1	**69.8**	CCP46348.1	**61.6**
31.	*kshB*	KAA5825925.1	WP_011598098.1	**50.9**	CCP46394.1	**54.6**
32.	*ltp2*	KAA5825927.1	WP_011596878.1	**76.2**	CCP46362.1	**74.9**
33.	*chsH1*	KAA5825928.1	WP_011596877.1	**53.2**	CCP46363.1	**53.9**
34.	*chsH2*	KAA5825929.1	WP_011596876.1	**54.9**	CCP46364.1	**52.4**
35.	*fadE29*	KAA5825930.1	WP_011596875.1	**71.8**	CCP46365.1	**68.5**
36.	*fadE28*	KAA5825036.1	WP_011596874.1	**52.9**	CCP46366.1	**45.1**
37.	*kstR*	KAA5825025.1	WP_009477547.1	**63.9**	CCP46397.1	**60.4**
38.	*echA20*	KAA5825028.1	WP_007300903.1	**62.7**	CCP46372.1	**60.7**
39.	*ipdA*	KAA5825029.1	WP_011597005.1	**63.2**	CCP46373.1	**59.5**

40.	*ipdB*	KAA5825030.1	WP_011597004.1	**54.5**	CCP46374.1	**58.5**
41.	*ipdC*	KAA5825038.1	WP_011597003.1	**63.0**	CCP46375.1	**65.3**
42.	*kstR2*	KAA5825039.1	WP_009477673.1	**48.3**	CCP46379.1	**43.4**
43.	*ipdF*	KAA5825031.1	WP_011596964.1	**63.0**	CCP46381.1	**62.6**
44.	*fadE30*	KAA5825032.1	WP_009477671.1	**68.3**	CCP46382.1	**65.2**
45.	*echA13*	KAA5825033.1	WP_005254492.1	**72.9**	CCP44702.1	**41.5**
46.	*fadE31*	KAA5825040.1	WP_011596961.1	**70.9**	CCP46384.1	**62.8**
47.	*fadE32*	KAA5825034.1	WP_011596960.1	**48.5**	CCP46385.1	**43.9**
48.	*fadE33*	KAA5825035.1	WP_011596959.1	**46.8**	CCP46386.1	**38.4**
49.	*hsaE3*	KAA5838019.1	WP_011597907.1	**58.8**	-	**-**
50.	*hsaG3*	KAA5838020.1	WP_011599026.1	**75.0**	-	**-**
51.	*hsaF3*	KAA5838021.1	WP_011599025.1	**82.4**	-	**-**
52.	*casA*	KAA5838022.1	WP_011597920.1	**44.1**	-	**-**
53.	*hsaD3*	KAA5838023.1	WP_009479043.1	**71.5**	-	**-**
54.	*kstR3*	KAA5838439.1	WP_011597904.1	**59.9**	-	**-**
55.	*kstD2*	KAA5838025.1	WP_011599760.1	**64.9**	-	**-**

56.	*kstD1*	KAA5838026.1	WP_011597919.1	**51.2**	-	**-**
57.	*kshA*	KAA5838028.1	WP_050787406.1	**68.3**	-	**-**
58.	*kshB*	KAA5838030.1	WP_011597937.1	**50.3**	-	**-**
59.	*ksdI*	KAA5838031.1	WP_011599721.1	**56.1**	-	**-**
60.	*hsaB3*	KAA5838032.1	WP_011597905.1	**60.8**	-	**-**
61.	*hsaA3*	KAA5838033.1	WP_009479048.1	**56.6**	-	**-**
62.	*hsaC3*	KAA5838034.1	WP_011597910.1	**60.5**	-	**-**
63.	*fadD3*	KAA5826116.1	WP_011596962.1	**58.9**	CCP46383.1	**57.1**
64.	*fadA6*	KAA5824600.1	WP_011596965.1	**71.2**	CCP46378.1	**68.1**
65.	*choD*	KAA5833518.1	WP_011598180.1	**62.8**	CCP46231.1	**65.4**
66.	*choE*	KAA5835081.1	WP_011596408.1	**33.0**	-	**-**
67.	*kstR3*	KAA5828487.1	WP_011597904.1	**62.9**	-	**-**
68.	*casA*	KAA5828430.1	WP_011597920.1	**49.8**	-	**-**
69.	*casC/chsE3*	KAA5828431.1	WP_011597921.1	**63.6**	CCP43000.1	**46.0**
70.	*casE*	KAA5828432.1	WP_011597922.1	**60.5**	-	**-**
71.	*3α-hsd*	KAA5828433.1	WP_011595206.1	**65.9**	-	**-**

72.	*casH*	KAA5828434.1	WP_011597925.1	**63.4**	CCP46180.1	**56.6**
73.	*casI*	KAA5828435.1	WP_011597926.1	**63.4**	-	**-**
74.	*casE*	KAA5838304.1	WP_011597922.1	**30.1**	-	**-**
75.	*casG*	KAA5838310.1	WP_011597924.1	**39.6**	-	**-**
