# Supplementary material for: Steroid Metabolism in Thermophilic Actinobacterium Saccharopolyspora hirsuta VKM Ac-666T
Source: Microorganisms. 2021 Dec 10;9(12):2554. doi: 10.3390/microorganisms9122554 (PMC8708139; doi:10.3390/microorganisms9122554)
Supplement: Supplementary file 1 [file microorganisms-09-02554-s001.zip › Supplementary Table S5.pdf]

Supporting materials

article title: Steroid Metabolism in Thermophilic Actinobacteria *Saccharopolyspora hirsuta* VKM Ac-666^T

journal name: Microorganisms

author names: Tatyana Lobastova, Victoria Fokina, Sergey Tarlachkov, Andrey Shutov, Eugeny Bragin, Alexey Kazantsev, Marina Donova

***Correspondence:** 2vvfokina@gmail.com

Supplementary Table S5. Actinobacterial strains with the similar proteins to the reference KshA, KshB, KstD of *M. tuberculosis* H37Rv.

No.	Strain	Protein_ID	Protein length	Query cover (%)	e-value	Number of identical amino acids	% of identical amino acids
**KshA**, NP_218043.1 *M. tuberculosis* H37Rv; protein length = **386 aa**							
**1**	*Thermomonospora curvata* DSM 43183	ACY99045.1	**386**	**97**	2.00E-146 (2e-165)	212	**54.9**
**2**	*Amycolatopsis granulosa* DSM 45669	NIH88125.1	**378**	**95**	1.00E-162 (0.0)	237	**62.7**

		NIH83855.1	**382**	**99**	7.00E-153 (1e-172)	225	**58.9**
**3**	*Thermocatellispora tengchongensis* DSM 45615	MBB5136868.1	**388**	**98**	2.00E-168 (0.0)	248	**63.9**
**4**	*Amycolatopsis methanolica* 239	WP_017983768.1	**377**	**95**	1.00E-163 (0.0)	239	**63.4**
**5**	*Amycolatopsis thermalba* strain 50.9b NODE_16	WP_094008249.1	**377**	**95**	3.00E-164 (0.0)	238	**63.1**
**6**	*Amycolatopsis ruanii* strain 49.3e NODE_6	WP_116109912.1	**377**	**95**	1.00E-162 (0.0)	238	**63.1**
**7**	*Microbispora siamensis* NBRC 104113	GIH66265.1	**371**	**93**	3.00E-150 (2e-167)	218	**58.8**
**KshB,** ANZ84337.1 *M. tuberculosis* H37Rv; protein length = **358 aa**							

**1**	*Thermomonospora curvata* DSM 43183	ACY99056.1	**351**	**92**	9.00E-106 (1e-134)	194	**55.2**
**2**	*Amycolatopsis granulosa* DSM 45669	NIH88123.1	**337**	**95**	1.00E-94 (8e-119)	183	**54.3**
		NIH83853.1	**363**	**94**	4.00E-88 (1e-113)	171	**47.1**
**3**	*Thermocatellispora tengchongensis* DSM 45615	MBB5136877.1	**344**	**92**	2.00E-102 (1e-127)	195	**56.7**
**4**	*Amycolatopsis methanolica* 239	WP_017983770.1	**337**	**95**	4.00E-95 (2e-125)	180	**53.4**
**5**	*Amycolatopsis thermalba* strain 50.9b NODE_16	WP_094008251.1	**337**	**95**	4.00E-97 (5e-128)	184	**54.6**
**6**	*Amycolatopsis ruanii* strain 49.3e NODE_6	WP_116109913.1	**337**	**95**	3.00E-96 (4e-127)	183	**54.3**

**7**	*Microbispora siamensis* NBRC 104113	GIH66241.1	**357**	**95**	5.00E-102 (7e-134)	197	**55.2**
**KstD,**<br>NP_218054.1 *M. tuberculosis* H37Rv; protein length = **563 aa**							
**1**	*Thermomonospora curvata* DSM 43183	ACY99057.1	**559**	**97**	6.00E-131 (5e-152)	266	**47.6**
**2**	*Amycolatopsis granulosa* DSM 45669	NIH88126.1	**555**	**98**	0.0	344	**62.0**
		NIH83847.1	**562**	**97**	8.00E-127 (2e-144)	245	**43.6**
**3**	*Thermocatellispora tengchongensis* DSM 45615	MBB5136872.1	**520**	**98**	2.00E-88 (3e-103)	216	**41.5**
**4**	*Amycolatopsis methanolica* 239	WP_017983767.1	**555**	**98**	0.0	348	**62.7**

**5**	*Amycolatopsis thermalba* strain 50.9b NODE_16	WP_094008248.1	**555**	**98**	0.0	349	**62.9**
**6**	*Amycolatopsis ruanii* strain 49.3e NODE_6	WP_116109911.1	**555**	**98**	0.0	349	**62.9**
**7**	*Microbispora siamensis* NBRC 104113	GIH66240.1	**557**	**97**	6.00E-132 (4e-154)	266	**47.8**
