# Supplementary material for: Steroid Metabolism in Thermophilic Actinobacterium Saccharopolyspora hirsuta VKM Ac-666T
Source: Microorganisms. 2021 Dec 10;9(12):2554. doi: 10.3390/microorganisms9122554 (PMC8708139; doi:10.3390/microorganisms9122554)
Supplement: Supplementary file 1 [file microorganisms-09-02554-s001.zip › Supplementary Table S6.pdf]

Supporting materials

article title: Steroid Metabolism in Thermophilic Actinobacteria *Saccharopolyspora hirsuta* VKM Ac-666[T]

journal name: Microorganisms

author names: Tatyana Lobastova, Victoria Fokina, Sergey Tarlachkov, Andrey Shutov, Eugeny Bragin, Alexey Kazantsev, Marina Donova

***Correspondence:** 2vvfokina@gmail.com

Supplementary Table S6. Result of BLAST search for the 29 protein sequences from the *M. tuberculosis* steroid catabolism enzymes in the *Geobacillus kaustophilus* HTA426 and *Parageobacillus thermoglucosidasius* DSM 2542 genomes.

No.	Protein	***Geobacillus kaustophilus* HTA426. BA000043.1**			***Parageobacillus thermoglucosidasius* DSM 2542. CP012712.1**		
		**Query cover (%)**	**Number of identical amino-acids**	**% of identical amino-acids**	**Query cover (%)**	**Number of identical amino-acids**	**% of identical amino-acids**
**1**	ChoD	6.7	16	**2.7**	5.7	16	**2.7**
**2**	Ltp4	6.5	12	**3.4**	9.6	12	**3.4**
**3**	Ltp3	22.1	24	**6.0**	18.5	24	**6.1**
**4**	Hsd4A	50.1	75	**23.6**	49.5	68	**21.4**

**5**	FadE26	73.0	101	**25.2**	73.0	95	**23.7**
**6**	FadE27	76.1	83	**22.2**	76.1	82	**22.0**
**7**	FadD17	87.8	125	**24.9**	70.3	106	**21.1**
**8**	EchA19	92.0	92	**35.0**	92.0	83	**31.6**
**9**	KshA	11.4	18	**4.7**	19.7	22	**5.7**
**10**	HsaF	25.1	27	**7.8**	93.9	167	**48.3**
**11**	HsaE	27.6	24	**9.2**	96.5	109	**41.8**
**12**	KstD	0	0	**0**	0	0	**0**
**13**	FadE29	85.2	113	**29.2**	89.1	111	**28.7**
**14**	FadE28	91.4	97	**28.6**	98.5	88	**26.0**
**15**	Cyp125	9.0	15	**3.4**	12.2	18	**4.2**
**16**	FadA5	95.9	184	**47.1**	96.2	177	**45.3**
**17**	EchA20	78.5	70	**28.3**	93.9	69	**27.9**
**18**	FadE30	83.6	105	**27.3**	85.7	103	**26.7**
**19**	FadD3	98.4	178	**35.1**	98.4	172	**33.9**
**20**	HsaB	80.7	55	**29.4**	78.6	52	**27.8**
**21**	HsaC	86.3	74	**24.7**	28.7	26	**8.7**
**22**	HsaD	86.6	71	**24.4**	92.8	91	**31.2**
**23**	HsaA	84.0	79	**20.0**	82.7	83	**21.1**

**24**	KshB	54.2	63	**17.6**	60.0	73	**20.4**
**25**	IpdB	6.0	32	**12.8**	15.2	14	**5.6**
**26**	ChsH1	12.4	46	**35.7**	25.6	15	**11.6**
**27**	ChsH2	31.5	28	**9.0**	21.9	25	**8.0**
**28**	FadD19	87.2	161	**29.3**	87.6	166	**30.2**
**29**	Hsd4B	31.8	27	**9.4**	31.8	26	**9.1**
